# Histone H3.3 subvariant H3mm15 is required for normal spermatogenesis by regulating gene expression

**DOI:** 10.1093/nar/gkag994

**Published:** 2026-11-09

**Authors:** Qianmei Wu, Akihito Harada, Cheng-Han Ho, Eisho Tanida, Kaori Tanaka, Kazumitsu Maehara, Bing Wang, Ryuki Shimada, Kei-ichiro Ishiguro, Haruhiko Ehara, Shun-ichi Sekine, Shinya Oki, Yuma Ito, Hitoshi Kurumizaka, Tatsuya Takemoto, Yasuyuki Ohkawa

**Affiliations:** Division of Transcriptomics, Medical Institute of Bioregulation, Kyushu University, 3-1-1 Maidashi, Higashi-ku, Fukuoka 812-0054, Japan; Division of Transcriptomics, Medical Institute of Bioregulation, Kyushu University, 3-1-1 Maidashi, Higashi-ku, Fukuoka 812-0054, Japan; Department of Multi-Omics, Graduate School of Medical Sciences, Kyushu University, 3-1-1 Maidashi, Higashi-ku, Fukuoka 812-0054, Japan; Laboratory of Chromatin Structure and Function, Institute for Quantitative Biosciences, The University of Tokyo, 1-1-1 Yayoi, Bunkyo-ku, Tokyo 113-0032, Japan; Laboratory of Chromatin Structure and Function, Institute for Quantitative Biosciences, The University of Tokyo, 1-1-1 Yayoi, Bunkyo-ku, Tokyo 113-0032, Japan; Division of Transcriptomics, Medical Institute of Bioregulation, Kyushu University, 3-1-1 Maidashi, Higashi-ku, Fukuoka 812-0054, Japan; Division of Transcriptomics, Medical Institute of Bioregulation, Kyushu University, 3-1-1 Maidashi, Higashi-ku, Fukuoka 812-0054, Japan; Department of Multi-Omics, Graduate School of Medical Sciences, Kyushu University, 3-1-1 Maidashi, Higashi-ku, Fukuoka 812-0054, Japan; Department of Multi-Omics, Graduate School of Medical Sciences, Kyushu University, 3-1-1 Maidashi, Higashi-ku, Fukuoka 812-0054, Japan; Department of Chromosome Biology, Institute of Molecular Embryology and Genetics (IMEG), Kumamoto University, 2-2-1 Honjo, Chuo-ku, Kumamoto 860-0811, Japan; Department of Biomedical Science, Graduate School of Medicine, Chiba University, 1-8-1 Inohana, Chuo-ku, Chiba 260-8670, Japan; Laboratory for Transcription Structural Biology, RIKEN Center for Integrative Medical Sciences, 1-7-22 Suehiro-cho, Tsurumi-ku, Yokohama 230-0045, Japan; Laboratory for Transcription Structural Biology, RIKEN Center for Integrative Medical Sciences, 1-7-22 Suehiro-cho, Tsurumi-ku, Yokohama 230-0045, Japan; Division of Functional Genomics, Institute of Resource Development and Analysis, Kumamoto University, 2-2-1 Honjo, Chuo-ku, Kumamoto 860-0811, Japan; High-Depth Omics Initiatives for Innovation, Joint Research Department, Medical Institute of Bioregulation, Kyushu University, 3-1-1 Maidashi, Higashi-ku, Fukuoka 812-0054, Japan; Laboratory of Chromatin Structure and Function, Institute for Quantitative Biosciences, The University of Tokyo, 1-1-1 Yayoi, Bunkyo-ku, Tokyo 113-0032, Japan; Laboratory for Embryology, Institute for Advanced Medical Sciences, Tokushima University, Tokushima 770-8503, Japan; Division of Transcriptomics, Medical Institute of Bioregulation, Kyushu University, 3-1-1 Maidashi, Higashi-ku, Fukuoka 812-0054, Japan

## Abstract

Histone H3 variants play a fundamental role in chromatin regulation, contributing to gene expression. While the functional diversity of H3.3 subvariants remains largely unexplored, we identified H3mm15 as a testis-expressed H3.3 subvariant that regulates meiotic transcription. Using RNA sequencing and variant-specific reverse-transcription quantitative PCR, we demonstrate here that H3mm15 is expressed predominantly in the testis. Loss of H3mm15 leads to seminiferous tubule disorganization and altered expression of spermatogenesis-related genes. Chromatin profiling reveals that H3mm15 is relatively enriched at promoter-proximal regions compared with H3.3, despite its lower overall chromatin incorporation and lower nucleosomal stability. Functional assays show that H3mm15 has a more moderate impact on transcriptional responses to stimuli than H3.3. Although H3mm15 forms a less stable nucleosome, its reduced stability does not confer enhanced transcription by RNAPII *in vitro*. Our findings suggest the existence of an H3.3 sub-barcode mechanism, in which structurally distinct H3.3 subvariants differentially regulate chromatin states and contribute to gene regulation during spermatogenesis.

## Graphical Abstract

**Graphical Abstract:**
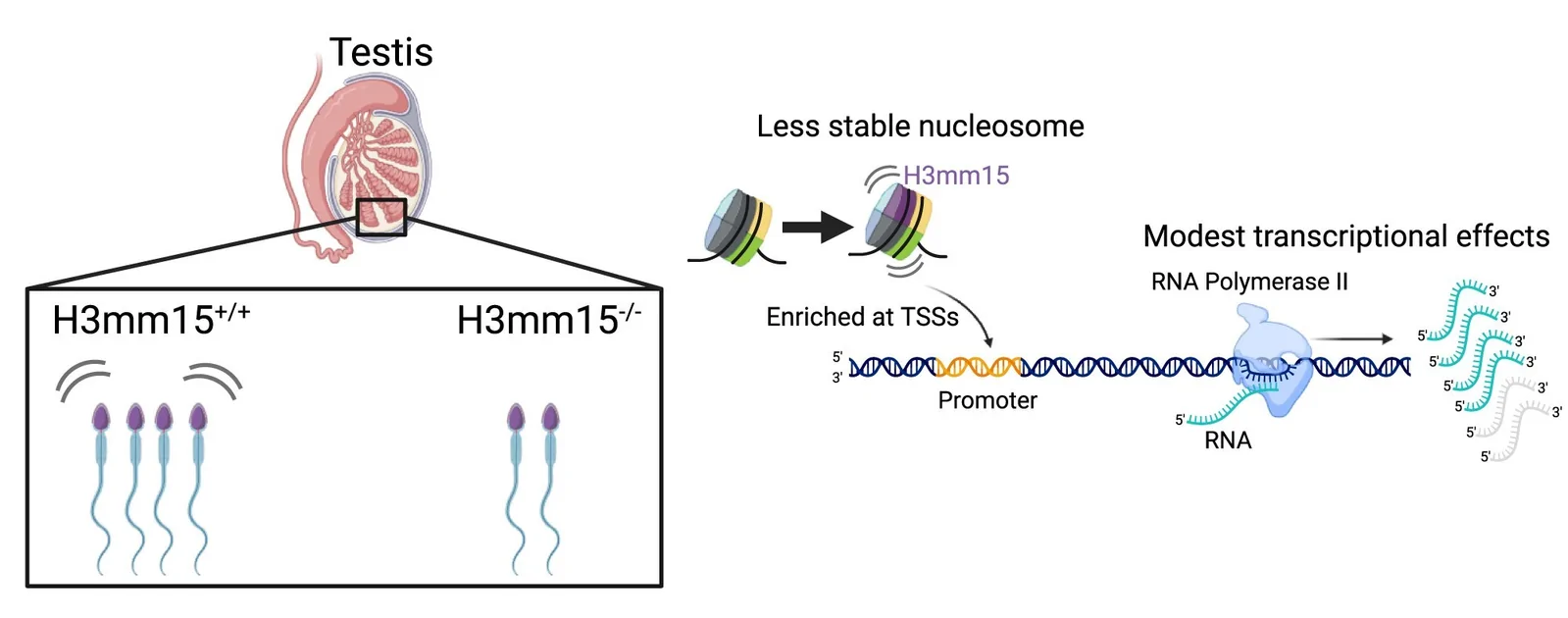

## Introduction

Histones are basic components of chromatin, where they assemble with 146 base pairs (bp) of DNA to form nucleosomes [1]. The canonical histones that form nucleosomes are H2A, H2B, H3, and H4. Histone variants also exist, with different amino acid sequences than the canonical histones. Histone variants can replace canonical histones within nucleosomes, altering chromatin structure and function. This enables histone variants to regulate key biological processes, including gene expression, DNA damage repair, chromatin remodeling, and cell differentiation [2–4]. Higher eukaryotes possess diverse histone variants, and novel variants are still being reported. Given their functional significance, comprehensive analysis of these histone variants is essential to uncover the fundamental principles of chromatin-mediated gene regulation.

Among the histone H3 variants, canonical H3.1/2 is deposited in a replication-dependent manner and contributes to chromatin maintenance, whereas H3.3 is incorporated independently of the cell cycle, marking transcriptionally active regions and telomeres [5–7]. In our previous work, we identified 13 novel histone H3.3 subvariants in the mouse genome, designated H3mm6–18 [8]. Functional characterization using fluorescence recovery after photobleaching (FRAP) analysis revealed that these variants differ in their chromatin incorporation properties. Some variants, such as H3mm7, show strong chromatin incorporation and promote skeletal muscle differentiation, while others, such as H3mm18, are diffusely localized in the nucleus and suppress differentiation [9, 10]. These findings suggest the existence of an “H3.3 sub-barcode” system, in which different H3.3 subvariants regulate chromatin through distinct incorporation patterns. However, the biological significance of this system remains largely unexplored.

Various histone variants are associated with spermatogenesis, with essential roles in chromatin remodeling. The replacement of canonical histones with testis-specific variants, such as H3t during meiosis and Th2a, Th2b, and H2a.l.2 during histone-to-protamine transition, is essential for germ cell development [11–13]. The importance of testis-specific histone variants is highlighted by knockout studies, where loss of H3t or H2a.l.2 results in male infertility [11–13]. While H2a.b-knockout mice exhibit milder transcriptional and spermatogenic defects, they display reduced fertility and increased offspring mortality [14, 15]. These studies suggest that testis-specific histone variants fine-tune the chromatin structure during spermatogenesis.

In this study, we investigate the function of H3mm15, which is predominantly expressed in the testis and plays a role in spermatogenesis. Loss of H3mm15 leads to a meiotic-disorganized transcriptional state in the testis. Furthermore, we show that H3mm15 differs from H3.3, showing promoter-driven genomic localization and structural instability, which explains the features of this mouse model. Our findings suggest the existence of an H3.3 sub-barcode mechanism, by which H3.3 subvariants with different chromatin stabilities are incorporated into specific genomic regions in place of H3.3, thereby altering the chromatin structure and regulating gene expression.

## Materials and methods

### Methodological overview

This study aimed to elucidate the functional role of the testis-expressed histone H3.3 subvariant H3mm15 in spermatogenesis. To achieve this, we employed both *in vivo* and *in vitro* systems to investigate H3mm15 expression, chromatin incorporation, transcriptional impact, and nucleosome stability.

We first assessed H3mm15 tissue specificity and transcript abundance using publicly available RNA-seq datasets and a custom variant-specific RT-qPCR system designed to discriminate H3mm15 from highly similar H3f3a transcripts. To determine the physiological role of H3mm15, we generated H3mm15-knockout (*H3mm15*^−/−^) mice using CRISPR–Cas9 and evaluated testis morphology, histology, sperm motility, and gene expression using bulk and spatial transcriptomics.

To explore the molecular mechanism of H3mm15 function, we used a Tet-On inducible NIH3T3 cell model expressing EGFP-tagged H3mm15 or H3.3. We compared their transcriptional responses to environmental stress (serum starvation and oxidative stress), chromatin incorporation levels, genomic localization via ChIL-seq, and chromatin accessibility by ATAC-seq. Nucleosome stability was further examined by *in vitro* reconstitution of H3mm15-containing nucleosomes and evaluation using thermal stability and MNase digestion assays. The cryo-EM structure of H3mm15 nucleosomes was solved to atomic resolution to identify a structural basis for nucleosome instability. We further examined potential transcriptional compensation in H3mm15-deficient testes, the histone-chaperone dependency of H3mm15 deposition, its genomic distribution in developing transgenic testes, the contribution of individual H3mm15-specific residues to chromatin dynamics, and the effect of H3mm15-containing nucleosomes on RNAPII transcription *in vitro*.

Together, this multi-level design integrates genetic, transcriptomic, biochemical, and structural approaches to define the contribution of H3mm15 to chromatin organization and gene regulation during spermatogenesis.

### Cells

NIH/3T3 Tet-On 3G (TaKaRa) cells were grown in Dulbecco’s modified Eagle’s medium (DMEM) supplemented with 10% Tet system-approved fetal bovine serum (FBS, TaKaRa).

### Generation of GFP-fused histone H3 variant cell lines

H3f3a and H3mm15 cDNAs were used for the expression of H3.3 and H3mm15 as previously reported [8, 9]. cDNAs were ligated into the Bidirectional Tet Expression Vector pT2A-TRETIBI (modified Clontech Tet-On system), which contains TolII transposon elements and an EGFP cDNA located downstream of the cDNA sequence (H3-EGFP: C-terminus). To examine the contribution of the three H3mm15-specific amino acid residues to its chromatin properties, H3mm15 single-reversion mutants were generated by replacing each H3mm15-specific residue with the corresponding residue in H3.3. The resulting mutants were designated Q100L, V112I, and C128R. Mutations were introduced by site-directed mutagenesis using KOD One^®^ PCR Master Mix (TOYOBO) and confirmed by Sanger sequencing. The mutant cDNAs were cloned into the same pT2A-TRETIBI vector used for H3mm15-EGFP. pT2A-TRETIBI/H3-EGFP variant transfection was performed with a Neon transfection system (Thermo Fisher Scientific). NIH3T3 cells were transfected with an expression vector and pCAGGS-TP encoding transposase (0.25 µg each of plasmid DNA per well, provided by Dr. Kawakami) and incubated for 24 h. To create cell lines with stable expression of H3-EGFP variants, the transfected cells were cultured for 7–21 days in the presence of 1 µg/ml puromycin. Doxycycline was added at a final concentration of 1 µg/ml for 24 h to induce the expression of H3-EGFP before use. EGFP-positive cells were selected using FACS.

### siRNA-mediated depletion of histone chaperones

NIH3T3 cells were cultured overnight in antibiotic-free medium and subjected to forward transfection according to the manufacturer’s instructions. Hira was depleted using ON-TARGETplus Mouse Hira (15 260) siRNA SMARTpool (L-043074–01-0005; Horizon Discovery) at a final concentration of 25 nM with DharmaFECT 3 Transfection Reagent (T-2003-02; Horizon Discovery). Daxx was depleted using ON-TARGETplus Mouse Daxx (13 163) siRNA SMARTpool (L-044803-00-0005; Horizon Discovery) at a final concentration of 50 nM with DharmaFECT 1 Transfection Reagent (T-2001-02; Horizon Discovery). The ON-TARGETplus Non-targeting Pool (D-001810-10-05; Horizon Discovery), used at the corresponding concentration and with the corresponding transfection reagent, served as the negative control. Doxycycline was added 6 h after transfection at a final concentration of 1 µg/ml. Cells were harvested 24 h after transfection for hydroxyapatite purification, immunoblotting, and GFP ChIL-seq.

### Fluorescence recovery after photobleaching analysis

Collagen Type I, rat tail (354 236; Corning), or Cellmatrix Type I-A collagen solution (type I collagen, 3 mg/ml, pH 3.0; Nitta Gelatin) was diluted tenfold with 1 mM HCl. Two milliliters of the diluted collagen solution was added to each 35-mm glass-bottom dish containing a No. 1.5 coverslip with a 14-mm glass diameter (P35G-1.5-14-C; MatTek). The dishes were incubated for 30 min at room temperature and washed twice with high-glucose DMEM without glutamine or phenol red (31 053 028; Gibco). Cells were seeded at a density of 1–3 × 10⁴ cells per dish and cultured in DMEM supplemented with 10% Tet-Approved FBS (631 101; Clontech). FRAP measurements were performed 1–3 days after seeding. FRAP measurements were performed using an AX R confocal microscope (Nikon) equipped with a 488-nm laser, a 60× Plan Apo λD objective lens (NA = 1.42), and a heated stage with a CO₂-control system (STXF-WSKMX-SET; Tokai Hit). Images were acquired at 256 × 256 pixels with a zoom factor of 11, a scan speed of 2 µs per pixel, a pinhole diameter of 153.3 µm, 0.1% laser power, an EM gain of 4, and a 503–514-nm linear variable emission filter. Eleven pre-bleach images were acquired at 500-ms intervals. A circular region with a diameter of 2 µm within a homogeneous region of the nucleoplasm was then photobleached using the 488-nm laser at 50% intensity, followed by acquisition of 90 post-bleach images at 500-ms intervals. Fluorescence intensities in the bleached region, the entire nucleus, and a background region were measured using NIS-Elements version 6.10.00. Background intensity was subtracted from both the bleached-region and whole-nucleus fluorescence intensities. Each value was normalized to its corresponding mean pre-bleach intensity, and the ratio of the normalized bleached-region intensity to the normalized whole-nucleus intensity was calculated to correct for fluorescence loss during image acquisition. Recovery curves were generated from individual cells and are presented as the mean ± S.D. The numbers of analyzed cells were as follows: H3.3-EGFP, *n* = 59; H3mm15-EGFP, *n* = 60; H3mm15 Q100L-EGFP, *n* = 90; H3mm15 V112I-EGFP, *n* = 30; and H3mm15 C128R-EGFP, *n* = 30. Cells were analyzed in three independent experiments performed on different culture days.

### Immunoprecipitation using hydroxyapatite chromatography

Analysis of nucleosome levels was performed using samples purified by hydroxyapatite after the nuclear-soluble fractions were removed, as described previously [16, 17]. Cells were washed twice with phosphate-buffered saline (PBS) and then lysed in ice-cold physiological buffer [PB (pH 7.4); 100 mM CH_3_COOK, 30 mM KCl, 10 mM Na_2_HPO_4_, 1 mM DTT, 1 mM MgCl_2_, 1 mM ATP, 0.1% Triton X-100, and protease inhibitor cocktail; Nacalai Tesque, Inc.]. The lysate was incubated for 10 min on ice and then centrifuged at 1000 × *g* for 5 min. The pellets were resuspended in BL buffer (5 mM HEPES, 200 mM KCl, 1 mM CaCl_2_, 1.5 mM MgCl_2_, 5% sucrose, and protease inhibitor cocktail; Nacalai Tesque, Inc.). The samples were sonicated and digested with micrococcal nuclease (2000 gel unit; New England Biolabs) and Ribonuclease A (10 µg; Nacalai Tesque, Inc.) at 37°C for 30 min. To stop the digestion reaction, ethylenediaminetetraacetic acid (EDTA) was added to the samples at a final concentration of 5 mM. NaCl was then added to achieve a concentration of 0.5 M, and the samples were transferred to a spin column including CHT Ceramic Hydroxyapatite Type I (80 µm; 100 mg; BIO-RAD) pre-washed with hydroxyapatite buffer 1 (HAPB1; 5 mM NaPO_4_, 600 mM KCl, and 1 mM EDTA). The chromatin/hydroxyapatite mixture was incubated on a rotator for 30 min at 4°C and washed twice with HAPB1 and three times with hydroxyapatite buffer 2 (HAPB2; 5 mM NaPO_4_, 100 mM KCl, and 1 mM EDTA). The purified nucleosomes were eluted with hydroxyapatite elution buffer (500 mM NaPO_4_, 100 mM KCl, and 1 mM EDTA). The nucleosomes were diluted 100-fold with BL buffer (with 0.5% NP-40 added). Then, 6× SDS sample buffer (Nacalai Tesque, Inc.) was added to a portion of the HAP-eluted samples and collected as HAP-IP input samples. HAP-eluted samples were incubated overnight at 4°C with 4 µg of rat monoclonal antibody against GFP (Bio Academia Co. Ltd., 1A5), which bound rabbit anti-rat IgG (Jackson ImmunoResearch Laboratories, West Grove, PA, USA) and Dynabeads M-280 Sheep anti-Rabbit IgG magnetic beads (Invitrogen). The beads were washed three times in BL buffer (with 0.5% NP-40 added) and eluted with 0.1 M glycine (pH 2.5). The eluted samples were neutralized with Tris–HCl (pH 8.0) and then precipitated with trichloroacetic acid (TCA). The precipitated samples were washed with acetone and then eluted with 2× SDS sample buffer.

### Immunoblotting

The samples were separated by SDS–PAGE (Extra PAGE One Precast Gel, 15%; Nacalai Tesque, Inc.) and transferred to a polyvinylidene fluoride membrane using a Trans-Blot Turbo Transfer System (Bio-Rad). The membranes were blocked for 1 h in 5% (w/v) skim milk in Tris-buffered saline containing 0.05% (v/v) Tween 20 (TBST), then incubated with primary antibodies diluted in Hikari Solution A (Nacalai Tesque, Inc.). After washing, the membranes were incubated with horseradish peroxidase (HRP)-conjugated secondary antibodies diluted in 5% (w/v) skim milk in TBST or Hikari Solution B (Nacalai Tesque, Inc.), as appropriate. Chemiluminescent signals were detected using Chemi-Lumi One Ultra or Chemi-Lumi One L (Nacalai Tesque, Inc.). The primary antibodies used were rabbit anti-H2B (NB100-56347, Novus Biologicals, 1:1000), goat anti-GFP (ab6673, Abcam, 1:1000), rat anti-H3-C (a gift from H. Kimura, 1:1000), mouse anti-HIRA (04-1488; Merck Millipore; 1:500), rabbit anti-DAXX (sc-7152; Santa Cruz Biotechnology; 1:250), rat anti-HSP90 (380-00 046; KISHIDA; 1:2500), and rabbit anti-H3K4me3 (07-473; Millipore; 1:1000). The secondary antibodies used were HRP-conjugated anti-rabbit IgG (ab6721, Abcam, 1:5000), anti-rat IgG (ab97057, Abcam, 1:20000), anti-goat IgG (ab6885, Abcam, 1:20000), anti-mouse IgG (ab97046, Abcam, 1:20000) and anti-rat IgM µ-chain-specific antibody (ab97180, Abcam, 1:20000).

### Chromatin integration labeling (ChIL)

ChIL-seq was performed as described previously [18]. Cells were prepared with 15 cm culture plates. Doxycycline was added at a final concentration of 1 µg/ml, 24 h in advance. Cells were collected with 0.1% bovine serum albumin (BSA) in PBS, and the EGFP-positive cells and negative cells were selected by FACS (SH800S, SONY). Then, 2000 selected cells/well were cultured in 96-well ibidi plates. Doxycycline was added at a final concentration of 1 µg/ml for EGFP-positive samples and incubated for 24 h. Library synthesis and amplification were performed using a SMART-Seq v4 Ultra Low Input RNA Kit (TaKaRa) following the manufacturer’s protocol with ChIL Read2 primers and Ad1 and Ad2 primers, as previously described [18]. The amplified DNA was purified using AMPure XP beads (Beckman Coulter). The quality of the resulting cDNA was assessed using a TapeStation 4200 system (Agilent Technologies) and quantitative PCR (qPCR). Sequencing was carried out using a NovaSeq 6000 SP Reagent Kit v1.5 (100 cycles) in an Illumina NovaSeq 6000 system with the following cycles: 51 cycles for read 1 and 51 cycles for read 2.

Adaptor sequences and low-quality reads were removed using Trim Galore, and reads shorter than 20 bp were discarded. The quality of raw and processed reads was assessed using FastQC. The filtered reads were aligned to the mm10 reference genome using Bowtie2. Only uniquely mapped reads were retained for further analysis. BAM files were sorted, indexed, and duplicate reads were removed using SAMtools. To generate genome-wide coverage tracks, deepTools was used to create BigWig files. To visualize ChIL-seq signals, deepTools was used to generate aggregation plots and heatmaps from BigWig files. Aggregation plots provided average signal profiles around genomic regions of interest, while heatmaps showed signal distribution across individual regions. For comparison with RNA expression levels, BRB-seq read counts for purified RNA were obtained, and normalized values were plotted using deepTools, aligning RNA expression patterns with ChIL-seq signal profiles. The read count distribution on the genome (e.g. promoters, exon, and intergenic regions) was evaluated using ChIPseeker (version 1.42.1).

For analysis of chaperone-dependent redistribution, peaks were called from the pooled ChIL-seq samples. For each histone variant–chaperone combination, siRNA-responsive regions were ranked according to the log2 fold change in ChIL-seq signal between the siRNA-treated and corresponding control samples. The top 1000 and bottom 1000 regions were defined as the relatively increased (UP) and relatively decreased (DOWN) groups, respectively. Genomic features were annotated using ChIPseeker, and the feature distributions of the UP and DOWN groups were compared using Fisher’s exact test for each feature category.

### Tissue-section ChIL-seq of H3mm15-EGFP transgenic testes

Testes were dissected from H3mm15-EGFP transgenic mice and non-transgenic littermate controls at postnatal day 14. The testes were snap-frozen in isopentane chilled with liquid nitrogen without prior fixation and stored at −80°C. Cryosections were prepared at a thickness of 10 µm using a CryoStar NX70 cryostat (Thermo Fisher Scientific) and mounted on Ibidi plates. Tissue-section ChIL (tsChIL) was performed as previously described [19], with modification of the fixation conditions. Sections were briefly washed with PBS and fixed with 2% paraformaldehyde containing 0.3% Triton X-100 in PBS for 5 min at room temperature. The sections were subsequently permeabilized with 0.3% Triton X-100 in PBS and blocked with Blocking One (Nacalai Tesque, Inc.). Sections were incubated overnight at 4°C with an in-house-purified rat monoclonal anti-GFP antibody (clone 1A5; 1 µg/ml). Sections were then incubated overnight at 4°C with a ChIL probe containing a T7 promoter and adaptor sequence at a final concentration of 5 µg/ml. Next, in-house Tn5 transposase was reacted with the sections at room temperature for 2 h. Fragment gaps were filled and ligated using T4 DNA polymerase and T4 DNA ligase. *In situ* transcription was performed using T7 RNA polymerase at 37°C for 3 h to amplify genomic sequences adjacent to the antibody-bound sites. The resulting amplified RNA was purified using RNAClean XP beads (Beckman Coulter). Sequencing libraries were prepared using a customized SMART-Seq v4-based ChIL library-construction protocol with ChIL-specific primers, as previously described [19]. Briefly, reverse transcription and cDNA amplification were performed using reagents from the SMART-Seq v4 Ultra Low Input RNA Kit (TaKaRa), with ChIL Read 2 and Ad1/Ad2 primers used for library amplification. Libraries were amplified for 15 PCR cycles, followed by column and bead purification to recover fragments ranging from 200 to 700 bp. The purified libraries were sequenced using an Illumina NextSeq 2000 system.

Adaptor sequences and low-quality bases were removed using Trim Galore, and reads shorter than 20 bp were discarded. The processed reads were aligned to the mm10 mouse reference genome using Bowtie2. Only uniquely mapped reads were retained for downstream analysis. Genome-wide coverage tracks were generated as BigWig files using the bamCoverage function in deepTools. BAM files from biological replicates were merged separately for the P14 testis tsChIL-seq and NIH3T3 ChIL-seq datasets before peak calling. Peaks were identified using MACS2 with the parameters -g mm –nomodel –nolambda -q 0.05, resulting in 2161 peaks for the testis dataset and 15 474 peaks for the NIH3T3 dataset. Peaks were assigned to genes when they overlapped regions extending ±2 kb from a TSS. Peaks that did not overlap any TSS-proximal region were classified as unannotated. When a peak overlapped the TSS-proximal regions of multiple genes, all corresponding genes were assigned. Genes assigned to at least one peak were defined as H3mm15-EGFP-associated genes. Common, testis-restricted, and NIH3T3-restricted gene sets were obtained by calculating the intersection and dataset-specific differences between the annotated testis and NIH3T3 gene sets. Gene Ontology Biological Process enrichment analysis was performed for the three gene sets using the compareCluster function in clusterProfiler (version 4.14.6) with enrichGO and the org.Mm.eg.db annotation database (version 3.20.0). No custom background gene set was specified, and the default background implemented by enrichGO was used. Enrichment results were visualized as dot plots. For gene-expression comparisons, DESeq2-normalized RNA-seq counts with non-zero values were log2-transformed. Within each dataset, the transformed expression values were standardized as *Z-*scores across all expressed genes and visualized as violin plots using ggplot2. Differences between gene groups were assessed using two-sided Wilcoxon rank-sum tests.

### Bulk RNA barcoding and sequencing (BRB-seq) for purified RNA

Cells were prepared with 15 cm culture plates. Doxycycline was added at a final concentration of 1 µg/ml, 24 h beforehand. Cells were collected with 0.1% BSA in PBS, and EGFP-positive cells or negative cells were selected by FACS (SH800S, SONY). Then, 50 000 of the selected cells were sorted into TRIzol^™^ Reagent (Ambion) and purified with Direct-zol RNA Microprep Kits (ZYMO RESEARCH). The quality of the total RNA was assessed using a TapeStation 4200 system (Agilent Technologies), and the concentration was measured using the Qubit^™^ RNA High Sensitivity (HS) Assay Kit (Invitrogen). BRB-seq was performed with 40 ng total RNA per sample. BRB-seq [20] was carried out as described previously, except that the cDNA was synthesized with reverse transcription (RT) primers for CEL-Seq2 [21] instead of those described in the original BRB-seq protocol. The quality of the resulting cDNA was assessed using a TapeStation 4200 system (Agilent Technologies) and qPCR. Sequencing was carried out using a MiniSeq High Output Kit (75 Cycles) in an Illumina MiniSeq system with the following cycles: 19 cycles for read 1 and 51 cycles for read 2.

### Bulk RNA barcoding and sequencing (BRB-seq) for cell-directed samples

Cells were prepared with 15 cm culture plates. Doxycycline was added at a final concentration of 1 µg/ml, 24 h beforehand. Cells were collected with 0.1% BSA in PBS, and EGFP-positive or negative cells were selected by FACS (SH800S, SONY). Then, 4000 selected cells/well were cultured in 96-well ibidi plates. Doxycycline was added at a final concentration of 1 µg/ml for EGFP-positive samples. After incubation for 24 h, the medium was changed to 10% FBS for control samples, 0% FBS for serum starvation treatment samples, or 250 µM for H_2_O_2_ treatment samples. Doxycycline was added at a final concentration of 1 µg/ml for EGFP-positive samples. Control samples and serum starvation treatment samples were incubated for 24 h. H_2_O_2_ treatment samples were incubated for 30 min. After incubation, cells were lysed with 150 µl/well of 1 × lysis buffer (TaKaRa) containing 80 U/ml Recombinant RNase Inhibitor (TaKaRa). Then, 30 µl lysate per sample was used to perform BRB-seq [20] as described previously, except that the cDNA was synthesized with RT primers for CEL-Seq2 [21] instead of those described in the original BRB-seq protocol. The quality of the resulting cDNA was assessed using a TapeStation 4200 system (Agilent Technologies) and qPCR. Sequencing was carried out using a NovaSeq 6000 SP Reagent Kit v1.5 (100 cycles) in an Illumina NovaSeq 6000 system with the following cycles: 19 cycles for read 1 and 81 cycles for read 2.

To evaluate the independent effects of experimental conditions on gene expression, the following linear model was considered using DESeq2: (Gene expression) = *β*_s_ × (starvation) + *β*_c _× (cell type). The categorical variables were (starvation): FBS+ or FBS− conditions, and (cell type): WT, H3.3-expressing, or H3mm15-expressing cells. The genes with FDR < 0.1 were considered differentially expressed genes (DEGs). The ashr package was also applied to reduce the variation of the estimated log fold-changes of genes with low counts.

### BRB-seq of H3mm15-EGFP transgenic testes

Testis cryosections were prepared from H3mm15-EGFP transgenic mice and non-transgenic littermate controls at postnatal day 14. Tissue sections with a combined thickness of 30 µm were lysed directly in TRIzol Reagent (Thermo Fisher Scientific), and total RNA was purified using the Direct-zol RNA Microprep Kit (Zymo Research). BRB-seq libraries were prepared as described above. Briefly, individual RNA samples were reverse-transcribed using barcoded oligo(dT) primers, after which the barcoded cDNAs were pooled, tagmented, and amplified by PCR. Read 1 contained a 10-nucleotide UMI followed by a 9-nucleotide sample barcode, and Read 2 contained an 81-nucleotide cDNA insert.

UMI and barcode sequences were extracted from Read 1 and assigned to the corresponding Read 2 using UMI-tools with the pattern NNNNNNNNNNCCCCCCCCC. Adaptor sequences and low-quality bases were removed using Trim Galore, and reads shorter than 20 bp were discarded. Reads were aligned to the GRCm38 reference genome using HISAT2. UMI-defined duplicates were removed using UMI-tools, and gene-level counts were generated using featureCounts.

### ATAC-seq

Cells were prepared with 15 cm culture plates. Doxycycline was added at a final concentration of 1 µg/ml, 24 h beforehand. Cells were collected with 0.1% BSA in PBS, and EGFP-positive or negative cells were selected by FACS (SH800S, SONY). Then, 50 000 selected cells/well were cultured in six-well plates. Doxycycline was added at a final concentration of 1 µg/ml for EGFP-positive samples. After 24 h of incubation, the medium was changed to 10% FBS for control samples or 0% FBS for serum starvation treatment samples, doxycycline was added at a final concentration of 1 µg/ml for EGFP-positive samples, and all samples were incubated for 24 h. After incubation, cells were collected and washed with cold PBS. Then, 50 000 cells were lysed using cold lysis buffer (1× NP40, 1× Tween 20, and 1× Digitonin). After 3 min incubation on ice, samples were spun at 500 × *g* for 10 min using a refrigerated centrifuge. Next, the pellet was resuspended in the transposase reaction mix (1× TAPS + DMF buffer, 100 nM transposase, 0.35× PBS, 1× Digitonin, and 1× Tween 20 in nuclease-free water). All samples were incubated for 30 min at 37°C and immediately placed on ice, followed by purification using a ZYMO DNA Clean & Concentrator Kit-5 kit (D4014, Zymo Research). Following purification, the library fragments were amplified using custom primers for a total of 15 cycles. Following PCR amplification, the libraries were purified using a ZYMO DNA Clean & Concentrator Kit-5 kit (D4014, Zymo Research). Sequencing was carried out using the NovaSeq 6000 SP Reagent Kit v1.5 (100 cycles) in an Illumina NovaSeq 6000 system with the following cycles: 51 cycles for read 1 and 51 cycles for read 2. The table of ATAC read counts falling within 500 bp upstream to 1500 bp downstream of the TSS of all genes was generated using *gtftools* (version 0.9.0) and *deeptools* (version 3.5.5). The read count table was normalized as gene-wise *Z*-scores after variance stabilizing transformation (“vsd” function in DESeq2). To quantify the degree of starvation in terms of the chromatin accessibility at genes, partial least squares discriminant analysis (PLS-DA; using the pls R package) was performed to determine the best section (2D plane) to discriminate the label of starvation (normal versus FBS− conditions). The cells with the Dox+ condition were excluded from the training of the model to avoid overfitting. Fisher linear discriminant analysis (LDA) was then performed on the 2D plane, and the degree of starvation was defined by the projection on the LD1 axis. The loading vector of PLS-DA was also used for gene set enrichment analysis (using the fgsea R package). The differential accessible genes were determined using DESeq2 with the option: fitType = “local.”

### Statistical inference of H3mm15 gene expression

A publicly available RNA-seq dataset from ENCODE/CSHL (NCBI SRA: SRR453077-SRR453175, SRR567478-SRR567503), containing 25 tissue types from embryonic and adult mice, was obtained. The transcript annotations of histone subvariants (H3mm genes) with a putative 3′-UTR of an additional 500 bp were manually added to the GENCODE transcript reference (release M20). To estimate the expression levels of *H3mm15* together with the read assignment ambiguity, we used Salmon (version 0.12.0) [22]. The quantification of gene expression using the Gibbs sampling from the posterior distribution of read counts: “salmon quant –validateMappings -l A –numGibbsSamples 1000,” was performed.

### Ethics statement

Experiments using mice were conducted in accordance with the Guidelines for the Care and Use of Laboratory Animals and were approved by the Institutional Animal Care and Use Committees (IACUCs) of Kyushu University and Tokushima University.

### Variant-specific RT-qPCR using a blocking oligonucleotide

Variant-specific RT-qPCR for *H3mm15* detection in tissue were performed as previously described [23, 24], with modifications for specificity validation. Total RNAs were extracted from 8-week-old C57BL/6 male mouse tissue, including testis, liver, brain, and skeletal muscle, with Sepasol-RNA I Super G (Nacalai Tesque, Inc.), and then treated with DNaseI (TaKaRa), followed by purification with an RNeasy MinElute Cleanup Kit (QIAGEN). The quality of the total RNA was assessed using a Bioanalyzer 2100 (Agilent Technologies), and the concentration was measured using the Qubit^™^ RNA High Sensitivity (HS) Assay Kit (Invitrogen). Total RNA was reversed-transcribed using PrimeScript II Reverse Transcriptase (TaKaRa) and an oligo(dT) primer, as previously described [8, 25]. qPCR was conducted using Thunderbird qPCR Mix (TOYOBO) with the following primers:

H3mm15-specific forward primer: 5′-GATACCAATCTGTGTGCTG-3′;

Universal-reverse primer: 5′-AGCACGTTCTCCGCGTATG-3′;

Pan-H3f3a-type primer (non-discriminatory, for total amplification [A]: 5′-CCTTTTTGAAGATACCAATCTGTGTGCTA-3′);

blocking oligonucleotide for *H3mm15* detection: 5′-ATCTGTGTGCTATCCATGCCAAA-3′ (3′-phosphorylated to prevent extension).

The reaction conditions were as follows: 95°C for 30 s, followed by 45 cycles of 95°C for 5 s, 55°C for 30 s, and 72°C for 30 s.

To validate the specificity of the variant-specific RT-qPCR assay, a titration experiment was performed using plasmids containing the CDSs of H3f3a and H3mm15. A fixed amount of H3f3a plasmid (10 ng) was mixed with serially diluted H3mm15 plasmid (10, 1, and 0.1 pg) and used as a template for variant-specific RT-qPCR. Under the [B] condition (H3mm15-specific primer with blocker), H3mm15 was detected in a concentration-dependent manner. In contrast, under the [A] condition (pan-H3f3a primer without blocker), amplification levels remained constant across conditions, confirming that [A] represents total H3f3a-type variant expression.

For tissue-derived samples, H3mm15 variant-specific RT-qPCR results were normalized to the corresponding signal obtained without the blocker (i.e. total H3f3a-type expression). The results are presented as the mean ± standard deviation of three independent experiments. The relationship between the *C*t values and histone concentration was analyzed using linear regression: *Ct* = *a* + *b* log(*concentration*). Statistical significance of the slope coefficient (*b*) was assessed using a two-sided *t*-test (H₀: *b* = 0).

### Quantitative RT-PCR

For *H3f3a* in mouse tissues, total RNAs were extracted from 8-week-old C57BL/6 male mouse tissues, including testis, liver, brain, and skeletal muscle, with Sepasol-RNA I Super G (Nacalai Tesque, Inc.), and then treated with DNaseI (TaKaRa), followed by purification with an RNeasy MinElute Cleanup Kit (QIAGEN). The quality of the total RNA was assessed using a Bioanalyzer 2100 (Agilent Technologies), and the concentration was measured using the Qubit^™^ RNA High Sensitivity (HS) Assay Kit (Invitrogen). Total RNA was reverse transcribed using PrimeScript II Reverse Transcriptase (TaKaRa) and an oligo(dT) primer, as previously described [8, 25]. *H3f3a* qPCR was performed using Thunderbird qPCR Mix (TOYOBO). *H3f3a* primer sequences were as follows: H3f3a-Fw: 5′-CCTTTTTGAAGATACCAATCTGTGTGCTA-3′; H3f3a-Rv: 5′-AGCACGTTCTCCGCGTATG-3′. qPCR data were normalized to *Gapdh* expression levels and presented as the mean ± standard deviation (S.D.) of three independent experiments. *Gapdh* primer sequences were as follows: Gapdh-Fw: 5′-GACTTCAACAGCAACTCCCACTCT-3′; Gapdh-Rv: 5′-GGTTTCTTACTCCTTGGAGGCCAT-3′.

For *Egfp* in EGFP-tagged H3.3 or H3mm15, cells were sorted, treated and lysed under the same conditions as the BRB-seq cell-directed samples. Lysed cells underwent reverse transcription using SuperScript IV Reverse Transcriptase (Invitrogen) and Oligo(dT)15 Primer (TaKaRa). qPCR was performed using Thunderbird qPCR Mix (TOYOBO). The *Egfp* primer sequences were as follows: Egfp-Fw: 5′-TGAACTTCAAGATCCGCCACAACA-3′; Egfp-Rv: 5′-GAACTCCAGCAGGACCATGTGATC-3′. qPCR data were normalized to *18S rRNA* expression levels and presented as the mean ± S.D. of three independent experiments. The *18S rRNA* primer sequences were as follows: 18SrRNA-Fw: 5′-GTAACCCGTTGAACCCCATT-3′; 18SrRNA-Rv: 5′-CCATCCAATCGGTAGTAGCG-3′.

### Generation of H3mm15 KO mice

*H3mm15* KO mice were generated with a genome editing technique as described previously, with several modifications [9, 44]. Briefly, *in vitro* fertilized zygotes (C57BL/6N × C57BL/6N) were electroporated with 100 ng/µl Cas9 protein, 100 ng/µl crRNAs, and 100 ng/µl tracrRNA. Two crRNAs targeting upstream and downstream of the *H3mm15* gene were used. We designed the target sequences of the two crRNAs as follows: H3mm15 crRNA1 (5′-GTTTCAGTTTGCTCGGGAGC(TGG)-3′); H3mm15 crRNA2 (5′-TGATCCCAGCGCACTCCTCT(AGG)-3′). Electroporated zygotes were transferred into the oviducts of pseudo-pregnant female mice, and the mutant mice were born on E19. As the founder mice were already a pure C57BL/6N background, no backcrossing was performed or required for the experiments.

### Generation of H3mm15-EGFP transgenic mice

A mouse H3mm15 cDNA was fused in frame to EGFP at its C terminus and inserted downstream of the CAG promoter and upstream of the SV40 polyadenylation signal in a piggyBac transposon vector. The transposon construct and piggyBac transposase mRNA were co-microinjected into fertilized mouse zygotes as previously described [26]. Male transgenic mice and wild-type littermates were analyzed at postnatal day 14.

### Hematoxylin and eosin (HE) staining

Testes were collected from 15-week-old mice. To prepare paraffin wax sections, testes were fixed in 4% paraformaldehyde and embedded using standard methods. Sections were prepared on slides (MATSUNAMI). The slides were dehydrated and stained with hematoxylin and eosin solutions. All images of the HE-stained sections were obtained with an all-in-one fluorescence microscope (BZ-X800, KEYENCE) using a Plan Apochromat 40× objective (NA0.95, BZ-PA40, KEYENCE).

### Johnsen’s score analysis

Johnsen’s score analysis was performed with HE-stained images. Each seminiferous tubule was evaluated and scored according to Johnsen’s scoring system [27, 28], which ranges from 1 to 10, with 10 indicating complete spermatogenesis and 1 indicating no germ cells and only Sertoli cells. For each biological replicate, a minimum of 50 tubular cross-sections were scored to obtain a representative average Johnsen’s score. Welch’s *t*-test analysis was conducted to compare the Johnsen’s scores across the *H3mm15*^+/+^ and *H3mm15^−^*^/^*^−^* groups. Statistical significance was set at a *P*-value <0.05.

### Computer-aided sperm analysis

This study employed the SMAS Animal system (DITEC Co., Ltd., Japan) for computer-aided sperm analysis (CASA) to assess the sperm motility and kinematic parameters. Briefly, sperm samples were collected from wild-type and *H3mm15*-deletion mice following standard procedures. The collected semen was immediately diluted in pre-warmed HTF medium and maintained at 37°C to preserve motility. A 10 µl aliquot of the sperm suspension was placed on a warmed glass slide, and a coverslip was gently positioned to create a chamber of controlled depth. The slide was then transferred to the SMAS Animal system for automated sperm tracking and analysis.

### Tissue preparation and immunostaining

Testes were collected from 15-week-old *H3mm15*^+/+^ and *H3mm15*^−/−^ mice for the analysis of adult testes. Frozen tissue samples stored at −80°C were used for the immunostaining procedures. Tissue sections were cut to 10 µm thick using a Cryostar NX70 (Thermo Fisher Scientific) and placed on microscope slides. Sections were fixed in 4% paraformaldehyde (PFA) in PBS at room temperature for 5 min, permeabilized with 0.3% Triton X-100 in PBS for 10 min at room temperature, and blocked using Blocking One (Nacalai Tesque, Inc.) for 20 min at room temperature. Primary antibody incubation was preceded by a mouse on mouse (M.O.M.) blocking with M.O.M.^®^ Immunodetection Kit (BMK-2202, VectorLabs), which involved the application of Mouse Ig blocking reagent in PBS for 1 h at room temperature, to reduce non-specific binding. After blocking, the sections were incubated with primary antibodies diluted in M.O.M.^®^ protein concentrate in PBS overnight at 4°C. After primary antibody application, sections were washed and incubated with M.O.M.^®^ protein concentrate in PBS containing both corresponding secondary antibodies conjugated with fluorophores (1/1000), and Nuclear Violet DCS1 dye (17 549, AAT Bioquest; 1/1000). Secondary antibodies were applied for 1 h at room temperature. All images were obtained with an all-in-one fluorescence microscope (BZ-X800, KEYENCE) using a Plan Apochromat 40 × objective (NA0.95, BZ-PA40, KEYENCE). Red fluorescence was detected using a TRITC filter (ex: 545/25 nm, em: 605/70 nm, dichroic: 565 nm, OP-87764, KEYENCE). Green fluorescence was detected using a GFP filter (ex: 470/40 nm, em: 525/50 nm, dichroic: 495 nm, OP-87763, KEYENCE). Blue fluorescence was detected using a DAPI filter (ex: 360/40 nm, em: 460/50 nm, dichroic: 400 nm, OP-87762, KEYENCE). The primary antibodies used included mouse anti-PLZF (2A9, ACTIVE MOTIF, 1:500), rabbit anti-Stra8 (ab49602, Abcam, 1:10 000), mouse anti-H3.1/2 [13] (1:1000), rat anti-H3.3 [13] (1:1000), mouse anti-gH2AX (JBW301, Merck Millipore, 1:500), rabbit anti-SCP3 (ab15093, Abcam, 1:1000), and rat anti-GCNA1 (ab82527, Abcam, 1:300). The secondary antibodies used included Alexa 555-conjugated goat anti-mouse (A32727, Invitrogen, 1:1000), Alexa 488-conjugated goat anti-rat (A21212, Invitrogen, 1:1000), and Alexa 488-conjugated goat anti-rabbit (A32731, Invitrogen, 1:1000).

For analysis of H3mm15-EGFP expression in developing testes, testes were collected from H3mm15-EGFP transgenic mice and wild-type littermates at postnatal day 14. Frozen sections were prepared, fixed, permeabilized, and blocked as described above. Intrinsic EGFP fluorescence was detected using rat anti-GFP followed by an Alexa 488-conjugated goat anti-rat secondary antibody (A21212, Invitrogen, 1:1000). STRA8 was detected using rabbit anti-STRA8 antibody (ab49602, Abcam; 1:10000) followed by an Alexa Fluor 555-conjugated goat anti-rabbit secondary antibody (A32732, Invitrogen; 1:1000). Nuclear DNA was visualized using Nuclear Violet DCS1 dye.

### Quantification of cell population observed in seminiferous tubules

Sertoli cells, spermatogonia (PLZF-positive), preleptotene (STRA8-positive), pachytene and diplotene spermatocytes (SYCP3-positive and sex-body-specific-gH2AX-positive), H3.1/3.2-positive germ cells, and H3.3-positive germ cells were measured from images of seminiferous tubules using the cell counter feature in the free Fiji ImageJ software (https://imagej.net). Seminiferous tubules had been divided into three groups, as previously described [29]. Considering that seminiferous tubule size is an issue, we calculated the “target cell amounts per Sertoli cell” for each tubule (Sertoli-related count). Multiple pairwise comparisons were performed using two-sample *t*-tests to evaluate the differences between groups. Given the potential for type I errors due to multiple testing, the false discovery rate (FDR) was controlled using the two-stage step-up method of Benjamini, Krieger, and Yekutieli, specifically designed to maintain control at a strict FDR of 1%.

### Flow cytometry and cell sorting of spermatogenic cells from adult testes

Spermatogenic cells were clustered with FACS, as in a previous report [30]. Testes were surgically removed from 15-week-old mice and immediately processed to strip off the tunica albuginea, exposing the seminiferous tubules. Each testis was then incubated in 6 ml of Gey’s Balanced Salt Solution (GBSS) (G9779, Sigma–Aldrich) containing 200 U/ml collagenase Type I (031-17601, Wako) and 5 µg/ml DNase I (04536282001, ROCHE) at room temperature for 15 min with intermittent pipetting to disaggregate the tubules. The tubules were then further digested by resuspending in 5 ml of GBSS with 250 µl of 2.5% trypsin (15090-046, Thermo Fisher Scientific) and 2.5 µl of DNase I, and incubated at 32°C for 15 min with gentle agitation. The enzymatic action was halted by the addition of FBS at a final concentration of 0.1%. The cell suspension was then filtered through a 40 µm mesh to remove undigested debris. Cells were washed and stained with 10 µg/ml Hoechst 33342 (H3570, Thermo Fisher Scientific). Stained cells were filtered through a 4 ml sorting filter and analyzed using a SONY SH800 sorter. Cells were directly sorted into 1.5 ml tubes containing Sepasol-RNA I Super G (Nacalai Tesque, Inc.), and then treated with DNaseI (TaKaRa), followed by purification with an RNeasy MinElute Cleanup Kit (QIAGEN). The quality of the total RNA was assessed using a Bioanalyzer 2100 (Agilent Technologies), and the concentration was measured using the Qubit^™^ RNA High Sensitivity (HS) Assay Kit (Invitrogen).

### Spermatogenic cell RNA-seq and data analysis

cDNA library preparation was performed using the SMART-seq Stranded Kit (TaKaRa) according to the manufacturer’s instructions. The quality of the resulting cDNA was assessed using a Bioanalyzer 2100 (Agilent Technologies) and qPCR. Sequencing was carried out using a NovaSeq 6000 SP Reagent Kit v1.5 (100 cycles) in an Illumina NovaSeq 6000 system with the following cycles: 51 cycles for read 1 and 51 cycles for read 2.

Adaptor sequences and low-quality reads were removed using Trim Galore, and reads <20 bp were discarded. The filtered reads were aligned to the GRCm38 reference genome using HISAT2, and the count matrix was obtained using featureCounts. Between-sample normalization was applied to the count matrix using the median of the ratios of geometric means. DEGs were extracted using DESeq2 with padj < 0.1 as a threshold. Gene-level expression counts were obtained using featureCounts from the Subread package, which efficiently counts mapped reads for genomic features. Normalized counts were used for downstream analysis. Principal component analysis (PCA) was performed using the “prcomp” function in R, identifying major sources of variation in the expression profiles. Clustering of expression profiles was performed using the “kmeans” function in R, grouping genes with similar expression patterns. Gene Ontology (GO) analysis was conducted using “clusterProfiler” in R, which identified enriched biological processes, molecular functions, and cellular components.

### Whole-testis RNA extraction and mRNA sequencing

Testes were collected from 10- and 13-week-old H3mm15 heterozygous (*H3mm15*^+/−^, *n* = 4) and homozygous knockout (*H3mm15*^−/−^, *n* = 3) mice derived from two independent litters and immediately frozen in liquid nitrogen. Frozen testes were pulverized using an SK mill (SK-200, Tokken, Inc.), and Sepasol-RNA I Super G (09379-84, Nacalai Tesque) was added to the pulverized tissue. Total RNA was purified from the resulting lysates using the Direct-zol RNA Microprep Kit (Zymo Research) according to the manufacturer’s instructions. RNA quality was assessed using a TapeStation 4200 system (Agilent Technologies), and RNA concentration was measured using the Qubit RNA Broad Range Assay Kit (Q10210, Invitrogen). For mRNA sequencing, 4 µg of total RNA from each sample was used for library preparation. Polyadenylated RNA was enriched using the KAPA mRNA Capture Kit (KK8440, Kapa Biosystems), and strand-specific cDNA libraries were prepared using the KAPA Stranded RNA-Seq Kit (KK8400, Kapa Biosystems) with NEBNext Adaptors for Illumina (E7337A, New England Biolabs). Library size distributions were assessed using a TapeStation 4200 system, and library concentrations were determined by quantitative PCR. Libraries were sequenced on an Illumina NovaSeq 6000 system using a NovaSeq 6000 SP Reagent Kit v1.5 (300 cycles). Paired-end sequencing was performed with 151 cycles for Read 1, 8 cycles for Index 1, 8 cycles for Index 2, and 151 cycles for Read 2. Adaptor sequences and low-quality reads were removed using Trim Galore, and reads <20 bp were discarded. The filtered reads were aligned to the GRCm38 reference genome using HISAT2, and gene-level counts were obtained using featureCounts. Differential expression analysis was performed using DESeq2, with *H3mm15*^+/−^ testes used as the reference group. Estimated log2 fold changes and 95% confidence intervals were calculated for *H3f3a, H3f3b, H3t*, and *H3mm6*–*H3mm18*.

### Photo-isolation chemistry for STRA8-positive cells

Photo-isolation chemistry (PIC) was performed as previously reported [31, 32]. Tissue samples were collected from both wild-type (WT) and knockout (KO) 15-week-old H3mm15 mice, with three biological replicates per group. Frozen sections were fixed in 4% PFA in PBS and were permeabilized with 5% Triton-X100 in 1× PBS. After being washed with PBS, samples were treated with 0.1 N HCl and neutralized with 1 M Tris–HCl (pH 8.0). After a reverse transcription (RT) reaction with PIC-customized primers, tissue samples were blocked using Blocking One (Nacalai Tesque, Inc.) at room temperature for 20 min and stained overnight at 4°C with rabbit anti-STRA8 antibody (ab49602, Abcam, 1:10000) in M.O.M.^®^ protein concentrate in PBS. This was followed by incubation with Alexa 488-conjugated goat anti-rabbit (A32731, Invitrogen, 1:1000) and Nuclear Violet DCS1 dye (17 549, AAT Bioquest, 1:1000) in M.O.M.^®^ protein concentrate in PBS at room temperature for 1 h. After washing with 1× PBS, slides were mounted. Three hundred STRA8-positive cells per sample were exposed using a multi-patterned illumination system using digital mirror devices (DMDs) as previously described [31] for 1 min per area. Following library preparation, amplification was performed as per the original protocol [31]. The quality of the resulting cDNA was assessed using a TapeStation 4200 system (Agilent Technologies) and qPCR. Sequencing was carried out using a NovaSeq 6000 SP Reagent Kit v1.5 (100 cycles) in an Illumina NovaSeq 6000 system with the following cycles: 19 cycles for read 1 and 81 cycles for read 2.

Adaptor sequences and low-quality reads were removed using Trim Galore, and reads shorter than 20 bp were discarded. The filtered reads were aligned to the GRCm38 reference genome using HISAT2, and the count matrix was obtained using featureCounts. Between-sample normalization was applied to the count matrix using the median of the ratios of geometric means. DEGs were extracted using DESeq2 with |log_2_FC| > 1 and padj < 0.1 as thresholds. Gene-level expression counts were obtained using featureCounts from the Subread package, which efficiently counts mapped reads for genomic features. Normalized counts were used for downstream analysis. Gene Ontology (GO) analyses were conducted using “clusterProfiler” in R, which identified enriched biological processes, molecular functions, and cellular components.

### Reconstitution of nucleosomes

Mouse histones H2A, H2B, H3.3, and H4 were purified as recombinant proteins from *Escherichia coli* by previously reported methods [33]. For H3mm15, the coding region was cloned into a pHCE vector, between the NdeI site and the BamHI site. The pHCE vector was also designed so that H3mm15 was N-terminally hexa-histidine tagged, with a PreScission site between the tag and the protein. H3mm15 was then purified similarly to other histones, except for using PreScission protease to cleave the hexa-histidine tags instead of thrombin protease, which was used in the purification of other histones.

To prepare the H3mm15 nucleosome, an H3mm15 octamer was first prepared, containing mouse H2A, H2B, H3mm15, and H4 histones. Then, the H3mm15 nucleosome was reconstituted by the salt dialysis method, in which Widom 601 DNA was mixed with the H3mm15 histone octamer. To prepare the H3.3 nucleosome, mouse histone H2A–H2B dimers and mouse H3.3–H4 tetramers were first prepared. Next, the H3.3 nucleosome was reconstituted using the salt dialysis method, in which Widom 601 DNA was mixed with the H2A–H2B dimers and H3.3–H4 tetramers. After reconstitution, the nucleosomes were purified separately with a Bio-Rad Prep Cell Apparatus to remove naked DNA and excessive histone octamers. Finally, the purified nucleosomes were dialyzed against 5% TCS buffer [20 mM Tris–HCl (pH 7.5), 1 mM DTT, and 5% glycerol] and flash frozen in liquid nitrogen. The nucleosomes were stored at −80°C before use.

### Sample preparation for cryo-EM

For cryo-EM analysis, PL2-6, a single-chain antibody fragment (scFv) that can bind to the nucleosome acidic patch and protect the nucleosome from the air–water interface during plunge-freezing [10, 34, 35], was added to the H3mm15 nucleosome. Then, 7.2 µl of 2.62 µM H3mm15 nucleosome was mixed with 1.8 µl of 41.92 µM PL2-6 (molar ratio of H3mm15 nucleosome:PL2-6 = 1:4), in a mixture of 26 mM Tris–HCl (pH 7.5), 0.8 mM DTT, 4% glycerol, 30 mM NaCl, and 0.2 mM EDTA. The mixture was then incubated at room temperature for 30 min.

Subsequently, 2.5 µL of the mixture was then applied to a Quantifoil R1.2/1.3 200 mesh copper grid, glow discharged for 1 min by a PIB-10 Bombarder (Vacuum Device Inc.) beforehand, inside the chamber of a Vitrobot Mark IV (Thermo Fisher Scientific) that was set to 4°C and 100% humidity. The grids were then blotted for 6 s with a blot force of 0. After that, the grids were plunge-frozen in liquid ethane and stored in liquid nitrogen before observation and data collection.

### Cryo-EM data collection

Data collection was performed by a Krios G4 cryo-TEM (Thermo Fisher Scientific), using the EPU software (Thermo Fisher Scientific). The microscope was operating at 300 kV at a nominal magnification of 81 000×, which corresponds to a pixel size of 1.06 Å/px on a K3 BioQuantum direct detection camera (Gatan). The defocus ranged from −1.25 to −2.25 µm.

An energy filter with a 20 eV slit was also applied. The total exposure time was 4.5 s, which was fractionated into 40 frames and corresponded to a total electron dose of 60.50 electrons per Å^2^. A total of 7510 micrographs were collected.

### Image processing

The collected images were processed in cryoSPARC [36]. The complete workflow can be found in Supplementary Fig. S19. Briefly, patch motion correction and patch CTF estimation were first performed. Micrographs of bad quality were removed manually. Subsequently, particles were picked by blob picker, extracted, and subjected to 2D classification. Junk particles were discarded, and a subset of the particles was used to generate an *ab initio* model. Using this model as a reference, selected particles after 2D classification were subjected to one round of heterogeneous refinement. The best class of particles was selected and further processed by reference-based motion correction, global CTF refinement, and local CTF refinement. A final non-uniform refinement job yielded a map with a resolution of 2.39 Å, which was estimated by the gold standard Fourier Shell Correlation (FSC) at FSC = 0.143 (Supplementary Fig. S20). For model building and visualization in figures, the map was further sharpened by DeepEMhancer [37]. The local resolution maps were calculated in cryoSPARC and visualized using UCSF ChimeraX [38]. More information on the processing statistics can be found in Table 1.

**Table 1.** Cryo–EM data collection and model statistics for the H3mm15 nucleosome–PL2–6 scFv

Sample	H3mm15 nucleosome with PL2-6
	EMD-63 765
	PDB: 9MB2
Data collection	
Electron microscope	Krios G4
Camera	K3 BioQuantum
Pixel size (Å/pixel)	1.06
Defocus range (µm)	−1.25 to −2.25
Total dose (e/Å^2^)	60.5
Movie frames (no.)	40
Total micrographs (no.)	7, 510
Reconstruction	
Software	CryoSPARC
Particles in the final map (no.)	1 973 565
Symmetry	C1
Final resolution (Å)	2.39
FSC threshold	0.143
Map sharpening	DeepEMhancer
Model building	
Software	Coot
Refinement	
Software	Phenix
Model composition	
Protein	755
Nucleotide	290
Validation	
MolProbity score	1.3
Clash score	4.61
R.m.s deviations	
Bond lengths (Å)	0.005
Bond angles (°)	0.588
Ramachandran plot	
Favored (%)	97.7
Allowed (%)	2.17
Outliers (%)	0.14

### Model building

The atomic model of the H3mm15 NCP was built based on the crystal structure of the *Xenopus laevis* NCP, which was reconstituted with the Widom 601 positioning sequence (PDB ID: 3LZ0) [39]. First, the 3LZ0 atomic model was fitted into the DeepEMhancer [37] map using UCSF ChimeraX [38]. Second, the atomic model was refined in Phenix [40], and edited manually in COOT [41, 42], to fix *X. laevis* histones into mouse histones and to fix outliers. The PL2-6 regions, although visible in the final cryo-EM map, were not included in the atomic model of the H3mm15 NCP. Structural figures were prepared with UCSF ChimeraX. Specifically, to remove PL2-6 density from the rest of the NCP, the atomic model was fitted into the map, and the “split map” function was used. More information on the modeling results can be found in Table 1.

### Thermal stability assay

The thermal stability assay was performed by first preparing 0.478 µg of NCP samples in 20 µl of reaction solution [19 mM Tris–HCl (pH 7.5), 0.95 mM DTT, 4.75% glycerol, 100 mM NaCl, and 1× SYPRO Orange (Sigma–Aldrich)] on a 96-well plate. Then, the plate was loaded into a QuantStudio 5 Real-Time PCR instrument (Thermo Fisher Scientific), and the fluorescent signal of SYPRO Orange was detected over a 25°C to 95°C temperature gradient, in steps of 1°C/min. Normalization of the signal was performed with the following formula: *F*(*T*)_normalized_ = [*F*(*T*) – *F*(25)]/[*F*(95) – *F*(25)], in which *F*(*T*) is the fluorescence signal intensity at a particular temperature.

### MNase assay

For the samples in which the MNase digestion time was larger than 0 min, 0.96 µg of NCP was mixed with 480 mU of MNase (TaKaRa) in 48 µl of reaction mixture [10 mM Tris–HCl (pH 7.5), 40 mM Tris–HCl (pH 8.0), 1.9 mM DTT, 2.5% glycerol, 25 mM NaCl, and 2.5 mM CaCl_2_]. The reaction was carried out at 37°C, and at the 3, 6, 9, and 12 min time points, a 10 µl aliquot was taken from the reaction mixture and mixed with 5 µl reaction stop solution [20 mM Tris–HCl (pH 8.0), 20 mM EDTA, 0.25% SDS, and 0.5 mg/ml Proteinase K (Sigma–Aldrich)]. The 0 min sample was prepared by first mixing the NCP (0.2 µg) with the reaction stop solution, before immediately adding MNase (100 mU). The resulting DNA fragments were analyzed by native-polyacrylamide gel electrophoresis (native-PAGE), using a 6% 0.2 × TBE gel. DNA fragments were visualized by ethidium bromide (EtBr) staining, and the gel images were acquired using an Amersham imager (GE Healthcare). Replicates of the assay can be found in Supplementary Fig. S21.

### *In vitro* nucleosome transcription assay

Nucleosomes containing either H2A, H2B, H3.3, and H4 or H2A, H2B, H3mm15, and H4 were reconstituted on a DNA fragment comprising a 153-bp modified Widom 601 sequence and a 45-bp linker containing a 9-bp mismatched region, as previously described [43]. RNA polymerase II (RNAPII) and TFIIS from *Komagataella pastoris* were purified as previously described [43]. Nucleosomes (0.1 µM) were transcribed with RNAPII (0.1 µM) at 30°C for 0, 5, 10, or 30 min in the presence of TFIIS (0.1 µM) and a fluorescently labeled RNA primer (0.2 µM; 5′-DY647-AUAAUUAGCUC-3′; Dharmacon). The transcription buffer contained 24 mM HEPES–KOH (pH 7.5), 50 mM potassium acetate, 0.2 µM zinc acetate, 20 µM tris(2-carboxyethyl)phosphine, 1.0% glycerol, 5 mM MgCl_2_, and 400 µM each of ATP, CTP, GTP, and UTP. At each time point, the reaction was terminated by addition of a deproteinization solution containing 100 mM Tris–HCl (pH 8.0), 150 mM EDTA, 4 M urea, and 1 µg/µl proteinase K (Roche), followed by incubation at room temperature for 10 min. Samples were mixed with Hi-Di formamide, heated at 95°C for 5 min, and analyzed by 10% denaturing polyacrylamide gel electrophoresis in 1 × TBE buffer. DY647-labeled RNA products were detected using an Amersham Typhoon biomolecular imager (Cytiva). Transcript band intensities were quantified using ImageJ version 1.54g (National Institutes of Health). Local background signals were subtracted from the measured band intensities. Run-off transcript intensities were analyzed separately. To compare RNA-product distributions, the intensity of each SHL(−5)-stalled, SHL(−1)-stalled, or run-off product was divided by the combined intensity of the three products at the corresponding time point. Three independent transcription assays were performed. Statistical significance between the H3.3 and H3mm15 nucleosomal templates at each time point was assessed using a two-sided unpaired Student’s *t*-test. Data are presented as the mean ± S.D.

### Statistical analysis

All statistical analyses were performed using R (version 4.1.2) or GraphPad Prism (version 9). Differential gene expression analysis from RNA-seq and BRB-seq data was conducted using DESeq2 with a false discovery rate (FDR) threshold of < 0.1 unless otherwise stated. The following linear model was applied to control for experimental variables: $Gene\ \textit{expression} = \beta \_s \times ( {\textit{starvation}} ) + \beta \_c \times ( {\textit{cell}\ \textit{type}} )$, where “starvation” is a categorical variable denoting FBS + or FBS − treatment, and “cell type” includes WT, H3.3-, or H3mm15-overexpressing cells. The ashr package was used to shrink the log_2_(fold change) estimates of low-expression genes.

Chromatin accessibility data from ATAC-seq were analyzed using DESeq2 (fitType = “local”), and the degree of chromatin remodeling under starvation was assessed by partial least squares discriminant analysis (PLS-DA) followed by LDA. Gene-wise accessibility scores were derived from *Z*-score normalized read counts spanning −500 to +1500 bp around the transcription start site (TSS).

For imaging-based quantification (e.g. Johnsen score, immunofluorescence, and sperm motility), unpaired Welch’s *t*-tests were used. For multigroup comparisons of spermatogenic cell populations, multiple pairwise *t*-tests were adjusted using the Benjamini–Krieger–Yekutieli method to control the false discovery rate at 1%. All tests were two-tailed, and significance was defined as *P* < 0.05 unless otherwise specified. Data are presented as mean ± standard deviation (S.D.) or standard error of the mean (S.E.M.), as indicated. Sample sizes and replicates are provided in the figure legends.

Statistical modeling for tissue-specific expression of H3mm15 was performed using Salmon (v0.12.0) with 1000 Gibbs sampling iterations to estimate posterior distributions of transcript abundances while accounting for ambiguity in read mapping due to high sequence similarity with H3f3a.

## Results

### H3mm15 is preferentially expressed in the testis

In our previous survey of mouse H3.3 subvariants, several variants, including *H3mm7, H3mm13*, and *H3mm15*, showed detectable expression in mouse tissues [8]. H3mm7 was subsequently shown to regulate skeletal muscle differentiation and regeneration, whereas the physiological function of H3mm15 remained unknown. The previous RNA sequencing (RNA-seq) data from ENCODE/Cold Spring Harbor Laboratory (CSHL) (GSE36025) survey also suggested *H3mm15* expression in the testis. Moreover, H3mm15 showed only partial localization to mitotic chromosomes in ectopic-expression assays, unlike H3mm7 and H3mm13, which showed more stable chromosomal localization. We therefore selected H3mm15 as an uncharacterized H3.3 subvariant with potentially distinct tissue expression and chromatin-association properties. Because the high sequence similarity between *H3mm15* and *H3f3a* complicates the assignment of short RNA-seq reads, we reanalyzed the ENCODE/CSHL dataset using an updated transcript annotation and probabilistic read assignment (Fig. 1A and Supplementary Fig. S1A). This analysis compared the expression levels of *H3mm15* across 25 different tissues. To assess the relative expression of *H3mm15* across the different tissues, transcripts per million (TPM) values were calculated and normalized as Z-scores. *H3mm15* showed low TPM values in all tissues, likely because of high sequence similarity with *H3f3a*, making it difficult to uniquely assign the sequencing reads (Supplementary Fig. S1A). Despite this limitation, the relative expression of *H3mm15* was highest in the testis (Fig. 1A), suggesting testis-enriched expression.

**Figure 1.**
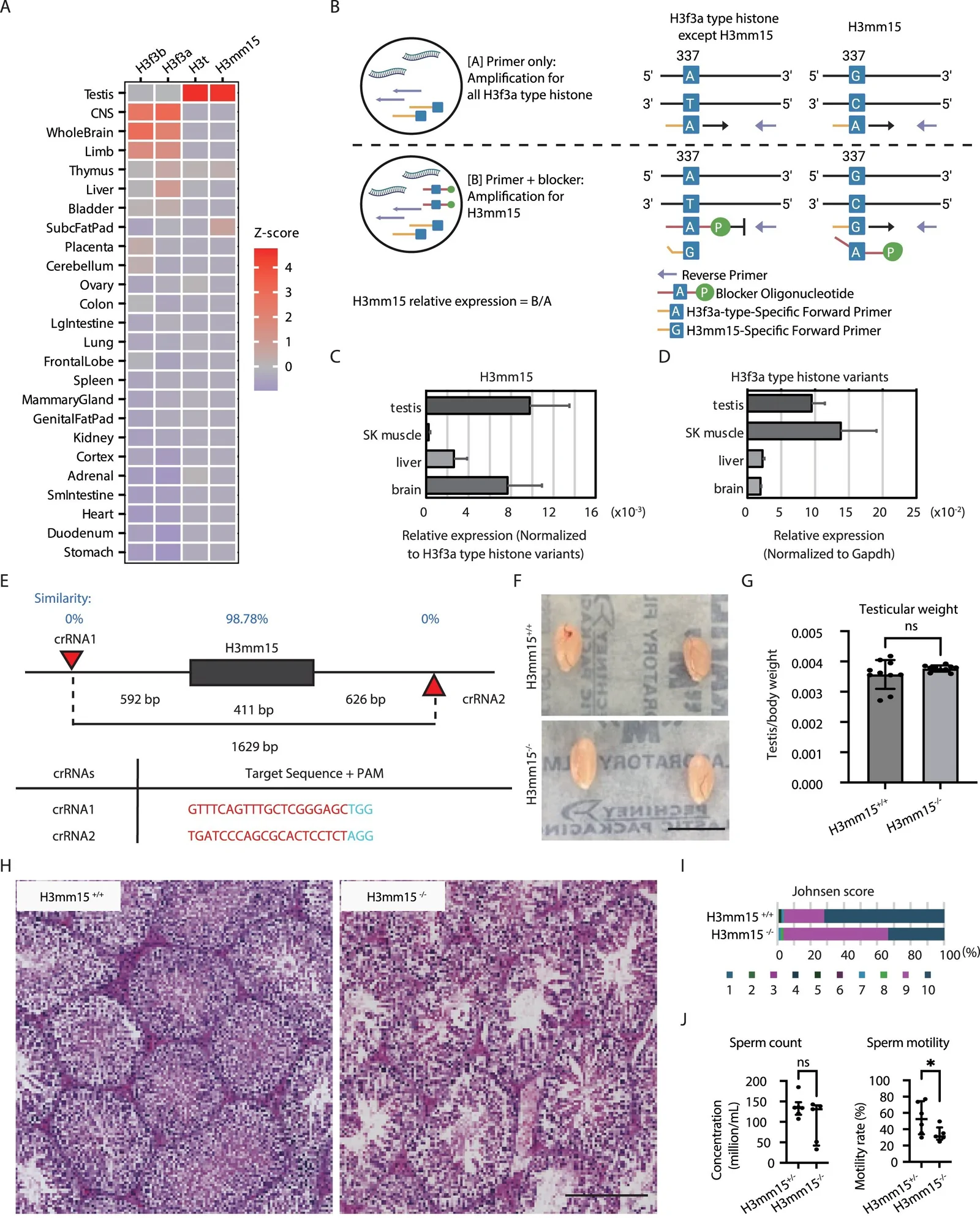
*H3mm15* deletion induced germinal epithelium disorganization in seminiferous tubules. (**A**) Expression levels of *H3mm15, H3f3b, H3f3a*, and *H3t* across 25 different tissues. Analysis of mouse *H3mm15* gene expression levels using Cold Spring Harbor Laboratory (CSHL) long RNA sequencing data (short-read) quantified using transcripts per million (TPM). The *Z*-score normalization was applied to the TPM values for each histone variant. (**B**) Overview of variant-specific RT-qPCR for specific detection of *H3mm15. H3mm15* differs from other *H3f3a*-type histone variants at position 337. A forward primer was designed to specifically target the G337 variant of *H3mm15*, while a 3′ phosphorylated blocking oligonucleotide was used to inhibit amplification of other *H3f3a*-type variants carrying the A337 base. [A] represents the total expression level of all *H3f3a*-type histone variants, including *H3mm15*, measured using a non-discriminatory primer without a blocker. [B] represents the specific expression of *H3mm15* using the variant-specific primer and blocker. The B/A ratio provides a quantitative estimate of the proportion of *H3mm15* expression relative to the total *H3f3a*-type histones. Created in BioRender. Harada, A. (2026) https://BioRender.com/hgndc6x. (**C**) Expression of *H3mm15* in various tissues based on variant-specific RT-qPCR. *H3mm15* expression was normalized to the expression of *H3f3a*-type histones measured without the blocker (condition [A]). Data represent the mean ± standard deviation (S.D.) from three independent experiments (*n* = 3). (**D**) Quantitative PCR (qPCR) results for *H3f3a*-type histone variants in different tissues. The expression levels of *H3f3a*-type histones were normalized to *Gapdh* expression (*n* = 3). The error bars indicate ± S.D. (**E**) The deletion of *H3mm15* by CRISPR. The sequence similarity of crRNA1, crRNA2, and the *H3mm15* CDS to other regions of the whole genome. A 0% similarity of crRNA1 and crRNA2 indicates that these crRNAs are specific to the target sequence used for *H3mm15* knockout. (**F**) *H3mm15*^−/−^ testes were similar in size to *H3mm15*^+/+^ testes. Images are representative of ten mice per genotype; scale bar: 1 cm. (**G**) *H3mm15*^−/−^ testes had a similar weight to *H3mm15*^+/+^ testes. The vertical axis represents testis/body weight (*n* = 10). (**H**) HE staining of seminiferous tubules in *H3mm15*^+/+^ and *H3mm15*^−/−^ testis sections; scale bar: 200 µm. (**I**) Johnsen score analysis of seminiferous tubules in *H3mm15*^+/+^ (*n* = 9) and *H3mm15*^−/−^ (*n* = 11) testis sections. The percentages of seminiferous tubules with each score are shown in a bar chart. (**J**) CASA of *H3mm15*^+/+^ (*n* = 6) and *H3mm15*^−/−^ (*n* = 5) mice. Unpaired Welch’s *t*-tests were performed to evaluate the differences between groups; *P < 0.1.

To confirm the preferential expression of *H3mm15* in the testis, we performed variant-specific RT-qPCR using a primer targeting position G337 of *H3mm15* and a blocking oligonucleotide to prevent amplification of other *H3f3a*-type variants (Fig. 1B). This variant-specific assay showed that *H3mm15* is expressed predominantly in the testis (Fig. 1C), while total *H3f3a*-type variants exhibited a broader expression profile, with high levels in skeletal muscle (Fig. 1D). To evaluate the specificity and degree of quantitation of the variant-specific RT-qPCR system, we conducted a titration assay using plasmids containing the coding sequences (CDSs) of H3f3a-type variants. A fixed amount of H3f3a-containing plasmid (10 ng) was mixed with serial dilutions of H3mm15 plasmid (10, 1, and 0.1 pg). Under the [B] condition (G337-targeting primer with blocking oligo), H3mm15 was detected in a dose-dependent manner (Supplementary Fig. S1B and C). In contrast, under the [A] condition, using a non-discriminatory primer that amplifies all H3f3a-type transcripts, amplification levels remained unchanged regardless of the H3mm15 concentration (Supplementary Fig. S1B and C). These results confirm that the [A] condition reliably reflects the total expression of H3f3a-type histones, and support the quantitative interpretation of the B/A ratio as a measure of H3mm15 expression. These findings, together with the RNA-seq data, indicate that *H3mm15* is particularly significant in the testis, suggesting a potential specialized role in this tissue.

### Deletion of H3mm15 leads to disorganization of the seminiferous tubules

To investigate *H3mm15* function in the testis, we generated *H3mm15*^−/−^ mice by a clustered regularly interspaced short palindromic repeat (CRISPR) method, as in previous reports [9, 44] (Fig. 1E). Specific targeting of the *H3mm15* gene was challenging because of the high sequence similarity between *H3mm15* and the CDS of *H3f3a* (97.78%). To overcome this, we designed two CRISPR RNAs (crRNAs) flanking the *H3mm15* coding region, which is specific to *H3mm15*. This resulted in a deletion of 1629 bp, from 592 bp upstream to 626 bp downstream of the *H3mm15* gene.

Loss of *H3mm15* did not affect fertility, and the testis size and the testis/body weight of *H3mm15*^−/−^ mice were indistinguishable from *H3mm15*^+/+^ mice (Fig. 1F,G). However, histological analysis with hematoxylin and eosin (HE) staining revealed disorganized seminiferous tubules in *H3mm15*^−/−^ testes, despite the presence of spermatogenic cells and spermatozoa (Fig. 1H). To determine the quality of the seminiferous tubules, we used the Johnsen Scoring System [27, 28], which scores the quality of the seminiferous tubules from 10 to 1 (Fig. 1I). This showed that the proportion of seminiferous tubules with a score of 10 was highest in *H3mm15*^+/+^ testes, indicating that complete spermatogenesis occurred in most of the seminiferous tubules. By contrast, *H3mm15*^−/−^ testes had the highest proportion of seminiferous tubules, with a score of 9, defined as “many spermatozoa present but germinal epithelium disorganized with marked sloughing or obliteration of lumen.” These results indicate that *H3mm15* deletion results in histopathological alterations of the seminiferous tubules.

Reduced Johnsen scores correlate with impaired sperm quality [45], and so we assessed sperm motility using CASA. While *H3mm15*^−/−^ mice showed only a mild reduction in sperm concentration, sperm motility was notably decreased compared with controls (Fig. 1J). These results suggest that, while H3mm15 deficiency does not affect fertility, it compromises sperm quality by impairing sperm motility.

### Deletion of H3mm15 leads to dysregulation of spermatogenesis-related gene expression

We next examined whether loss of *H3mm15* affects germ cell populations. No apparent differences were observed in the numbers of GCNA-positive spermatogenic cells between *H3mm15*^−/−^ and *H3mm15*^+/+^ testes (Supplementary Fig. S2A), indicating that the germ cell compartment was preserved. Similarly, the numbers of PLZF-positive spermatogonia, STRA8-positive preleptotene spermatocytes, and gH2AX/SYCP3-positive pachytene spermatocytes remained unchanged (Supplementary Fig. S2B), suggesting that the major spermatogenic cell populations were maintained in *H3mm15*^−/−^ testes. To assess whether H3mm15 loss affected the distribution of other histone variants, we performed immunofluorescence staining for canonical H3.1/2 and H3.3. No overt changes in the abundance or localization of these histones were observed in H3mm15^−/−^ testes (Supplementary Fig. S2C). However, the anti-H3.3 antibody used in this study recognizes H3f3a/b as well as H3mm15; therefore, the lack of a detectable reduction in H3.3 signal upon H3mm15 loss implies that H3mm15 comprises only a minor fraction of total H3.3-type histones *in vivo*. This is consistent with our variant-specific PCR and transcriptome data (Supplementary Fig. S1B and C), which demonstrate that H3mm15 is expressed at substantially lower levels than H3f3a or H3f3b in testicular tissue. These results collectively suggest that H3mm15 contributes modestly to the total H3.3 signal, and that H3f3a/b account for most of the H3.3 signal detected in H3mm15^−/−^ testes.

To determine whether loss of *H3mm15* induces compensatory expression of other H3.3-type variants, we performed an independent whole-testis mRNA-seq analysis using *H3mm15*^+/−^ littermate controls (*n* = 4) and *H3mm15*^−/−^ mice (*n* = 3) derived from two independent litters at 10 and 13 weeks of age. Variant-specific RT-qPCR detected H3mm15 transcripts in *H3mm15*^+/−^ testes, whereas H3mm15 expression was absent or nearly undetectable in *H3mm15*^−/−^ testes (Supplementary Fig. S3A). Consistently, whole-testis mRNA-seq showed a marked reduction in *H3mm15* expression in the knockout testes. No significant upregulation of *H3f3a, H3f3b, H3t*, or the other reliably quantified *H3mm* variants was detected in *H3mm15*^−/−^ testes (Supplementary Fig. S3B). These results provide no evidence for robust transcriptional compensation by other H3.3-type variants at the whole-testis level following *H3mm15* deletion.

While these data indicate that the cellular composition and global histone variant expression patterns remain largely intact in *H3mm15*^−/−^ testes, they do not rule out functional impairments at the molecular level. Notably, *H3mm15*^−/−^ mice exhibited a significant reduction in sperm motility despite only a mild reduction in sperm concentration (Fig. 1J), suggesting potential defects in the differentiation or functionality of spermatogenic cells. This prompted us to investigate whether gene expression programs underlying spermatogenesis were altered in the absence of H3mm15.

Given that no changes in cell types were observed, we investigated the gene expression changes during spermatogenesis. We performed RNA-seq on spermatogenic cells sorted by fluorescence-activated cell sorter (FACS) into the spermatogonia (SG), preleptotene (preL), leptotene/zygotene (LZ), pachytene/diplotene (PD), meiosis II (MII), and round spermatid (RS) stages (Fig. 2A and Supplementary Fig. S4). Notably, H3mm15 transcripts could not be detected in the sorted populations by RNA-seq, likely due to its low expression level and high sequence similarity with H3f3a. This technical limitation hindered direct quantification of H3mm15 across the spermatogenic stages in this dataset. PCA revealed distinct clustering of the early (SG, preL), middle (LZ, PD), and late (MII, RS) stages, with differences between *H3mm15*^−/−^ and *H3mm15*^+/+^ in each group (Fig. 2B). We investigated the DEGs in each group (Fig. 2C). Based on the number of DEGs, *H3mm15* deletion caused global gene expression changes, most pronounced at the preleptotene and subsequent stages (Fig. 2C). *K*-means clustering identified four expression patterns, revealing that *H3mm15*^−/−^ showed altered gene expression before and after meiosis that eventually returned to similar levels as in *H3mm15*^+/+^, except for cluster 2 (Fig. 2D). These results indicate that *H3mm15* deletion results in expression changes at the preleptotene stage in all four clusters, regardless of the gene expression patterns during spermatogenesis.

**Figure 2.**
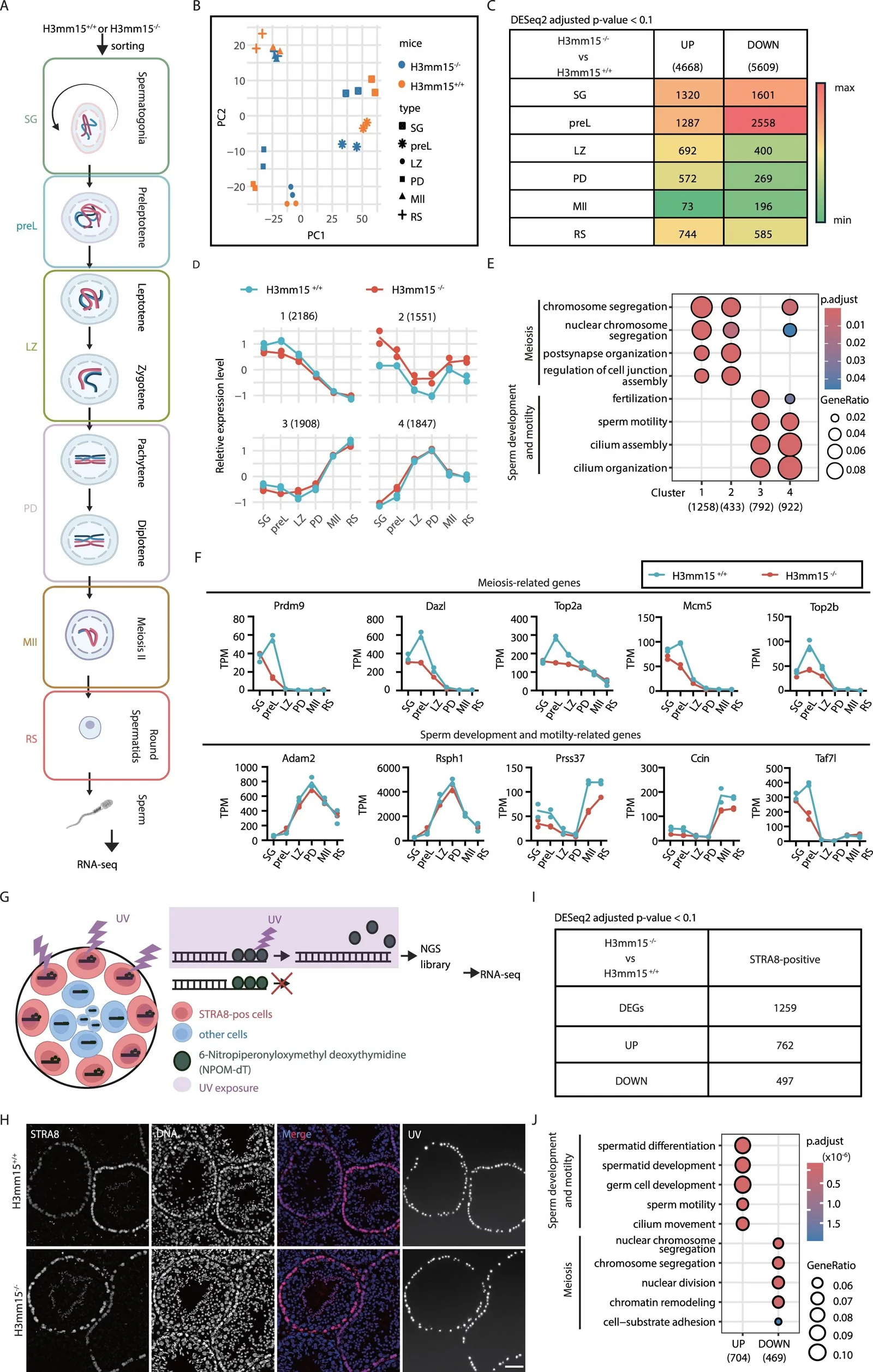
Deletion of H3mm15 resulted in disordered gene expression during spermatogenesis. (**A**) Overview of *H3mm15*^+/+^ and *H3mm15*^−/−^ spermatogenic cells grouped by FACS. Spermatogenic cells from *H3mm15*^+/+^ and *H3mm15*^−/−^ testes were divided into six groups depending on their stage of the spermatogenesis process. SG, spermatogonia; preL, preleptotene; LZ, leptotene/zygotene; PD, pachytene/diplotene; MII, meiosis II; RS, round spermatids. Two independent experiments were performed. Created in BioRender. Harada, A. (2026) https://BioRender.com/2827jba. (**B**) PCA of the six groups from *H3mm15*^+/+^ and *H3mm15*^−/−^ spermatogenic cells. (**C**) DEG numbers for *H3mm15*^−/−^ in each group. The numbers of DEGs are indicated by a color bar on the right. (**D**) The DEGs were divided into four clusters by *k*-means based on the gene expression patterns through spermatogenesis. The vertical axis represents the relative expression levels, and the horizontal axis represents the cell types. (**E**) Gene Ontology (GO) analysis of the genes in each *k*-means cluster shown in (D) that were differentially expressed between *H3mm15*^+/+^ and *H3mm15*^−/−^ cells at the preleptotene stage. Representative enriched biological process terms are shown. Circle size represents the gene ratio, and color indicates the adjusted *P-*value (Benjamini–Hochberg false discovery rate). The numbers in parentheses below each cluster indicate the numbers of preleptotene-stage DEGs assigned to the respective clusters. The top five GO terms for the upregulated and downregulated DEGs with *P* < 0.1 are listed in order of gene count. (**F**) The kinetics of meiosis-related and sperm development and motility-related DEGs. The vertical axis represents transcripts per million (TPM), and the horizontal axis represents the stage of spermatogenesis. (**G**) Overview of PIC for STRA8-positive cells. Transcripts in STRA8-positive cells were specifically amplified into next-generation sequencing (NGS) libraries and analyzed by RNA-seq. Three independent experiments were performed. Created in BioRender. Harada, A. (2026) https://BioRender.com/61f399s. (**H**) STRA8-positive cells were photo-irradiated by UV during PIC. STRA8 immunofluorescence was performed on *H3mm15*^−/−^ and *H3mm15*^+/+^ testis sections, and STRA8-positive cells were exposed to UV for a PIC reaction. Images are representative of three biologically independent mice per genotype; scale bar: 50 µm. (**I**) The number of DEGs for *H3mm15*^−/−^ STRA8-positive cells. (**J**) GO analysis of the DEGs in *H3mm15*^−/−^ STRA8-positive cells. The top five GO terms for the upregulated and downregulated DEGs satisfying *P* < 0.1 are listed in order of gene count.

To determine the biological functions associated with each temporal pattern, we performed Gene Ontology (GO) analysis separately for the genes in each cluster that were differentially expressed between *H3mm15^+/+^* and *H3mm15^−/−^* cells at the preleptotene stage, where the largest transcriptomic changes were observed (Fig. 2E). Clusters 1 and 2 were predominantly enriched for meiosis-related processes, including chromosome segregation and nuclear chromosome segregation. By contrast, clusters 3 and 4 were enriched for sperm development- and motility-related processes, including fertilization, sperm motility, cilium assembly, and cilium organization (Fig. 2E). Cluster 4 also showed enrichment for chromosome segregation-related terms. These results indicate that H3mm15 deletion perturbs multiple functionally distinct transcriptional programs at the preleptotene stage, including gene programs associated with meiosis and subsequent sperm development. Several genes involved in meiotic progression and chromosome regulation, including *Prdm9, Dazl, Top2a, Mcm5*, and *Top2b* [46–50], were among the downregulated genes (Fig. 2F), raising the possibility that *H3mm15* deficiency may affect the transcriptional program associated with early meiosis. In addition to these meiosis-related genes, genes associated with sperm development and motility were also found among the preleptotene DEGs (Fig. 2E). For instance, genes typically expressed at later primary spermatogenic stages, such as *Adam2* and *Rsph1*, showed precocious upregulation at the preleptotene stage, whereas *Prss37* and *Ccin* exhibited reduced expression across the preL, MII, and RS stages. These genes play essential roles in sperm maturation and function [51–54]. Among the downregulated genes, *Taf7l*, a transcription factor essential for the expression of sperm motility-related genes [55, 56], showed an expression pattern similar to those of meiosis-related genes. Although we cannot establish causality, the downregulation of *Taf7l* may reflect broader transcriptional misregulation during spermatogenesis and could potentially contribute to the observed sperm motility defects in *H3mm15*^−/−^ testes.

To validate the preleptotene gene expression changes in *H3mm15*^−/−^ testes, we used PIC, a spatial transcriptomic method that enables region-specific gene profiling [31] under non-enzymatic and histologically preserved conditions (Fig. 2G). While FACS-based sorting enables the enrichment of cell populations based on DNA content and Hoechst dye profiles, it is susceptible to potential contamination between adjacent spermatogenic stages because of partial overlaps in nuclear morphology. In contrast, PIC allows direct photo-isolation and transcriptomic capture from immunostained STRA8-positive preleptotene cells in tissue sections [57] (Fig. 2H), thereby minimizing stage contamination. PIC analysis identified 1259 DEGs (762 upregulated, 497 downregulated) in *H3mm15*^−/−^ STRA8-positive cells (Fig. 2I). GO terms such as “chromosome segregation” and “chromatin remodeling” (downregulated) and “sperm development” and “motility” (upregulated) overlapped substantially with those found in the FACS-sorted RNA-seq analysis (Fig. 2E,J), supporting the reproducibility of both approaches. These results demonstrate that PIC serves as a complementary method to FACS, providing spatial validation and increased specificity for transcriptomic changes associated with the mitosis-to-meiosis transition during spermatogenesis.

### H3mm15 regulates gene expression in response to perturbation more moderately than H3.3

To investigate the functional properties of H3mm15 in gene regulation and chromatin dynamics, we used an NIH3T3 cell-based overexpression model. This approach was chosen because of the lack of a specific antibody for H3mm15 and the technical challenges associated with isolating sufficient testis-derived cells for chromatin-based assays. While NIH3T3 fibroblasts do not fully recapitulate the chromatin environment of germ cells, they provide a controllable and reproducible system to evaluate fundamental properties such as chromatin incorporation, nucleosome stability, and transcriptional responsiveness to external stimuli. Variant-specific RT-qPCR showed that H3mm15 transcripts were endogenously detected in NIH3T3 cells, although their relative abundance was lower than that in whole testis, corresponding to approximately 45% of the whole-testis level under the same assay conditions (Supplementary Fig. S5).

Using this system, we generated enhanced green fluorescent protein (EGFP)-tagged, H3mm15-overexpressing NIH3T3 cells (H3mm15-EGFP) and EGFP-tagged, H3.3-overexpressing NIH3T3 cells (H3.3-EGFP) as controls, under the regulation of a Tet-On 3G inducible expression system (Fig. 3A). Both H3mm15-EGFP and H3.3-EGFP localized to the nucleus, indicating that the tagged proteins associate with chromatin (Fig. 3B). Under steady-state conditions, H3mm15 overexpression did not induce notable changes in gene expression (Supplementary Fig. S6). By contrast, in the testis, deletion of H3mm15 led to marked transcriptional dysregulation during the mitosis-to-meiosis transition, a period associated with major chromatin remodeling and double-strand breaks [58, 59]. This contrast suggests that the transcriptional effects of H3mm15 may become apparent in response to specific physiological stimuli.

**Figure 3.**
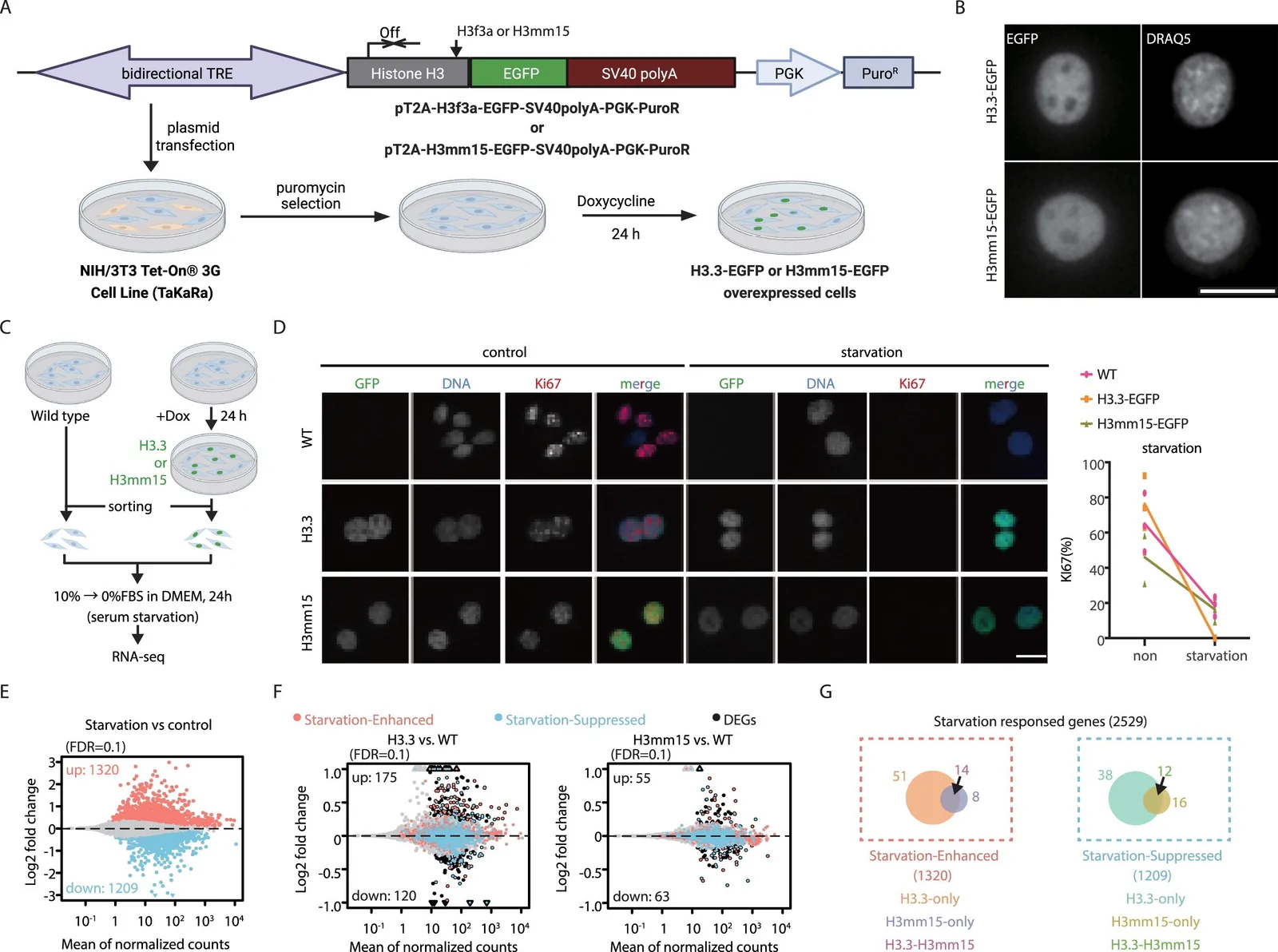
H3mm15-overexpressing cells showed more modest changes in gene expression in response to serum starvation than H3.3-overexpressing cells. (**A**) Overview of the generation of H3mm15-EGFP- or H3.3-EGFP-overexpressing cells. Created in BioRender. Harada, A. (2026) https://BioRender.com/5yas4xc. (**B**) Images of GFP-tagged histone H3 variants showing their localization in the nucleus. Representative fluorescence images from three independent cell preparations are shown; scale bar: 20 µm. (**C**) Overview of the serum starvation treatment experiment with wild-type (WT), H3.3-EGFP-overexpressing, and H3mm15-EGFP-overexpressing cells. Three independent experiments were performed. Created in BioRender. Harada, A. (2026) https://BioRender.com/qucx0et. (**D**) Serum starvation treatment resulted in cell cycle arrest in all three cell types. Immunostaining for a cell cycle marker (Ki67), rescued histone variants (GFP), and the nucleus (DRAQ5) are shown. Ki67-positive cells were counted before and after serum starvation treatment and the results are shown on the right. Representative images from three independent experiments are shown. Ki67-positive cells were quantified in three independent experiments, and each point represents one independent experiment; scale bar: 20 µm. (**E**) MA plot showing transcriptome profiles based on serum starvation treatment. (**F**) MA plot showing the transcriptome profiles based on H3.3 or H3mm15 expression in starvation-treated and untreated samples. (**G**) Venn charts showing the genes induced by histone expression among the starvation-related genes.

To test this hypothesis *in vitro*, we subjected the cells to serum starvation, a well-established method for inducing cell cycle arrest [60–62] (Fig. 3C). Serum starvation resulted in reduced Ki67 positivity in all three cell lines, confirming cell cycle arrest (Fig. 3D). RNA-seq analysis showed that starvation induced substantial transcriptional changes, with 1320 genes upregulated and 1209 genes downregulated [false discovery rate (FDR) = 0.1] (Fig. 3E). Upon expression of H3 variants, H3.3 had a greater transcriptional impact (175 upregulated and 120 downregulated genes) than H3mm15 (55 upregulated and 63 downregulated genes), indicating that H3mm15 exerts a more moderate influence on gene expression in this context (Fig. 3F). When the starvation-responsive genes were classified according to whether their changes were enhanced or suppressed by histone-variant expression, approximately half of the H3mm15-associated genes overlapped with H3.3-associated genes, including 14 starvation-enhanced and 12 starvation-suppressed genes (Fig. 3G). These results suggest a partially shared but attenuated transcriptional effect of H3mm15 relative to H3.3.

We further examined transcriptional responses under DNA damage conditions by treating the cells with hydrogen peroxide (H_2_O_2_), which induces oxidative stress and DNA strand breaks [63, 64] (Supplementary Fig. S7A). Following H_2_O_2_ treatment, gH2AX staining confirmed DNA damage in all three cell types (Supplementary Fig. S7B). Transcriptome changes were milder than those observed with serum starvation, likely reflecting the shorter treatment duration. Using a relaxed threshold (FDR = 0.3), we identified 120 upregulated and 199 downregulated genes (Supplementary Fig. S7C). H3.3 expression again induced a greater number of DEGs (1141 upregulated and 805 downregulated genes) than H3mm15 expression (105 upregulated and 79 downregulated genes) (Supplementary Fig. S7D). A substantial proportion of the H3mm15-associated genes overlapped with the corresponding H3.3-associated gene sets, consistent with the serum-starvation results (Supplementary Fig. S7E).

We next examined whether the effects associated with H3.3-EGFP and H3mm15-EGFP were conserved across the two stress conditions. Ten genes were consistently upregulated by both variants under serum-starvation and H_2_O_2_-treatment conditions, whereas no genes were consistently downregulated by both variants (Supplementary Fig. S7F,G). Thus, although most transcriptional effects were stress-dependent, a limited but reproducible shared component was observed among the upregulated genes. Together, these results indicate that H3mm15 elicits a more restricted transcriptional response than H3.3, with partial overlap that is biased toward transcriptional induction.

### H3mm15 is incorporated into chromatin at lower levels than H3.3

H3mm15 showed a more moderate gene regulatory function than H3.3 when cells were stimulated. Therefore, we next determined whether H3mm15 functions through chromatin incorporation. To investigate H3mm15 incorporation into chromatin, we purified nucleosomes from H3 variant-expressing cells (H3.3 Dox+ and H3mm15 Dox+) by hydroxyapatite chromatography (HAP) and immunopurification by anti-GFP antibodies (HAP-IP) (Fig. 4A). We performed the same experiments on wild-type (WT) cells and doxycycline-untreated cells (H3.3 Dox− and H3mm15 Dox−) as controls. Histones were detected by Coomassie Brilliant Blue (CBB) staining of the HAP-purified samples (Supplementary Fig. S8A), indicating that nucleosomes were purified by HAP. Hydroxyapatite purification was performed before immunoprecipitation because this step enriches nucleosomes while removing soluble and non-nucleosomal histones, as established previously [16, 17]. In addition, histones were detected in samples of H3.3- and H3mm15-expressing cells immunopurified by anti-GFP antibodies (Supplementary Fig. S8B), indicating that both H3.3-EGFP- and H3mm15-EGFP-containing nucleosomes were purified by the HAP-IP. We did not detect any EGFP-tagged histones on CBB staining (Supplementary Fig. S8B). This may be because the amount was below the detectable range of CBB. Thus, we performed immunoblotting of the HAP-purified and HAP-IP nucleosomes with anti-H2B or anti-GFP antibodies (Fig. 4B). This detected H2B signals in the HAP samples of the five cells, and H3.3-EGFP and H3mm15-EGFP signals with a size of 44 kDa in the HAP-purified and HAP-IP nucleosomes of H3.3 Dox+ and H3mm15 Dox+ cells, respectively (Fig. 4B). These results indicate that both H3.3-EGFP and H3mm15-EGFP were recovered in HAP-purified nucleosomal fractions and were associated with H2B, supporting their incorporation into nucleosome particles. However, these experiments did not determine whether the tagged histones formed homotypic nucleosomes or heterotypic nucleosomes containing endogenous H3.

**Figure 4.**
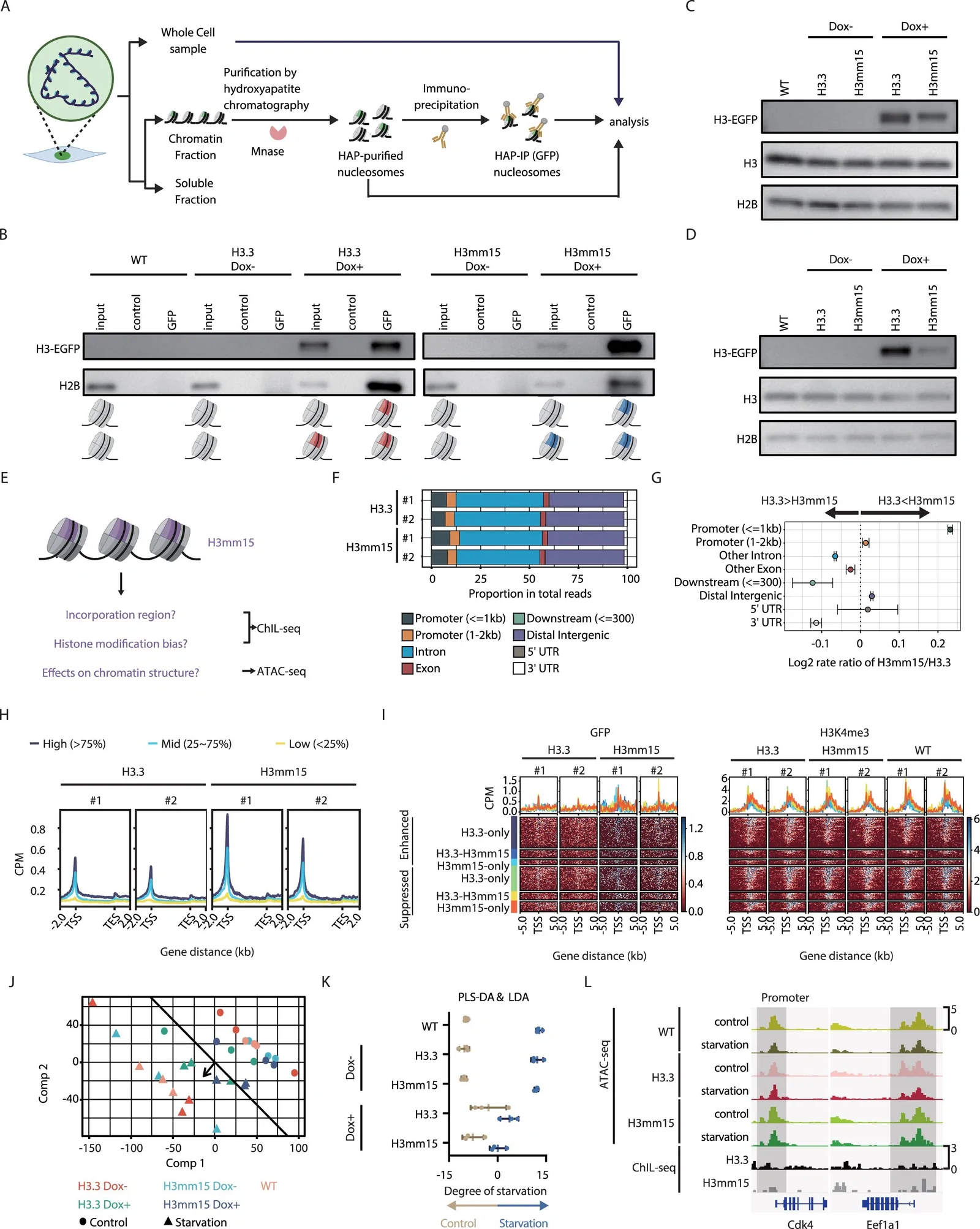
H3mm15 is incorporated into promoter regions at a low level and moderately regulates chromatin accessibility. (**A**) Overview of immunoprecipitation using hydroxyapatite chromatography (HAP-IP) of GFP-tagged histone variants with anti-GFP. Created in BioRender. Harada, A. (2026) https://BioRender.com/lnkgu6x. (**B**) Immunoblot results of HAP and HAP-IP samples for WT, H3.3 Dox−, H3.3 Dox+, H3mm15 Dox−, and H3mm15 Dox + NIH3T3 cells. Red, H3.3-type nucleosomes; blue, H3mm15-type nucleosomes. Anti-H2B and anti-GFP were used to detect histones in all samples. Representative results from two independent experiments are shown. Created in BioRender. Harada, A. (2026) https://BioRender.com/uose86t. (**C** and **D**) Immunoblot results of whole cell samples (C) and HAP samples (D) for the five cell types. Anti-GFP, anti-H3, and anti-H2B antibodies were used to detect histones. Representative results from two independent experiments are shown. (**E**) Experimental workflow for investigating the epigenetic function of H3mm15. Two independent ChIL-seq and three independent ATAC-seq experiments were performed. Created in BioRender. Harada, A. (2026) https://BioRender.com/27uni20. (**F**) Graphical representation of the distribution of overexpressed H3mm15 and H3.3 in the mouse genome; 5′-UTR, 5′-untranslated region; 3′-UTR, 3′-untranslated region. (**G**) Log_2_ rate ratio of H3mm15-EGFP/H3.3-EGFP read proportions (each point) across genome annotations, with 95% confidence intervals (error bars). Estimates were obtained using Poisson regression with offset log(M) to adjust for varying total read counts (M). Data were obtained from two independent ChIL-seq experiments. (**H**) Both H3.3-EGFP and H3mm15-EGFP were enriched around the TSS and related to the gene expression level. The vertical axis represents counts per million mapped reads (CPM), and the horizontal axis represents the region of the gene body. (**I**) Comparison of the incorporation level of EGFP-tagged histone variants and the H3K4me3 levels of the genes in Fig. 3G. (**J**) PLS-DA and LDA of the chromatin accessibility profiles in the five cell types under both control and starvation conditions. Samples are categorized by cell type and treatment condition. The dividing line represents the distinction between the control and starvation conditions based on LDA. The arrow indicates the transition from control to starvation conditions. (**K**) PLS-DA and LDA of the chromatin accessibility profiles in the five cell types under control and starvation conditions. This analysis is based on the data from Fig. 4J. The *x*-axis represents the degree of starvation, with 0 indicating the dividing line between control and starvation conditions, as shown in Fig. 4J. The error bars indicate ± S.D. of three independent ATAC-seq experiments. (**L**) An IGV snapshot of the ATAC-seq data showing the chromatin accessibility changes of a cell cycle-related gene (*Cdk4*) upon serum starvation treatment and histone variant overexpression at the promoter region.

Although the whole-cell mRNA and protein expression levels of H3.3-EGFP and H3mm15-EGFP were comparable (Supplementary Fig. S9 and Fig. 4C), substantially less H3mm15-EGFP was recovered in the HAP-purified nucleosomal fraction (Fig. 4C,D). After normalization to total histone and nucleosomal contents, the relative incorporation of H3mm15-EGFP was approximately 19.2% of that of H3.3-EGFP. These results indicate that H3mm15-EGFP is incorporated into nucleosomes less efficiently or is less stably retained in the nucleosomal fraction than H3.3-EGFP.

### H3mm15-EGFP shows promoter-proximal enrichment in NIH3T3 cells and transgenic testis

To further characterize the epigenetic properties of H3mm15 incorporation, we performed Chromatin Integration Labeling followed by sequencing (ChIL-seq) [18] to determine its genomic distribution and its association with promoter-related histone modifications (Fig. 4E). Because of the lack of an H3mm15-specific antibody, we performed GFP-ChIL-seq on H3mm15-EGFP- and H3.3-EGFP-expressing cells. This revealed that both H3.3 and H3mm15 were enriched at promoter regions (Fig. 4F and Supplementary Fig. S10). Interestingly, H3mm15-EGFP showed relative promoter-proximal enrichment compared with H3.3-EGFP (Fig. 4G and Supplementary Fig. S10).

We next examined the relationship between gene expression and histone-variant incorporation. We divided the genes into three groups based on their expression levels in H3.3-EGFP- or H3mm15-EGFP-expressing cells [high- (>75%), middle- (25%–75%), and low-expressed (<25%) genes; excluding those with DEseq2-normalized counts = 0], and investigated the incorporation pattern of H3mm15 and H3.3 based on the gene expression levels (Fig. 4H). Both H3.3-EGFP and H3mm15-EGFP showed expression-dependent enrichment around transcription start sites (TSSs), with stronger signals associated with more highly expressed genes. Thus, despite its lower overall incorporation, H3mm15-EGFP was relatively enriched in promoter-proximal regions of transcriptionally active genes.

We next examined the chaperone dependency of H3mm15-EGFP deposition by siRNA-mediated depletion of the major replication-independent H3.3 chaperones HIRA and DAXX. Immunoblotting confirmed efficient and specific depletion of each chaperone (Supplementary Fig. S11A). Analysis of hydroxyapatite-purified nucleosome fractions revealed distinct chaperone dependencies: HIRA depletion reduced the chromatin incorporation of H3.3-EGFP but had little effect on H3mm15-EGFP, whereas DAXX depletion reduced H3mm15-EGFP incorporation but not H3.3-EGFP incorporation (Supplementary Fig. S11B). These results indicate that H3mm15-EGFP incorporation was more sensitive to DAXX depletion than to HIRA depletion under these conditions, suggesting that DAXX contributes to H3mm15 deposition. At the individual-region level, GFP ChIL-seq did not detect statistically significant changes following either knockdown. However, genomic annotation analysis revealed a significant redistribution specifically after DAXX depletion in H3mm15-EGFP-expressing cells: regions showing increased relative signal were enriched for promoter-proximal annotations and depleted for distal intergenic annotations (Supplementary Fig. S11C). This pattern suggests that DAXX preferentially contributes to H3mm15-EGFP deposition at distal and gene-body regions, whereas its promoter-proximal enrichment is relatively preserved after partial DAXX depletion.

Having characterized the chaperone dependency and genomic distribution of H3mm15-EGFP in NIH3T3 cells, we next examined whether its promoter-proximal distribution was also observed in testicular chromatin *in vivo*. We generated CAG-driven H3mm15-EGFP transgenic mice using the piggyBac system (Supplementary Fig. S12A). Testes were collected at postnatal day 14, before completion of the first wave of spermatogenesis and the appearance of mature spermatozoa. Clear EGFP fluorescence was detected in transgenic testes but not in wild-type littermates (Supplementary Fig. S12B). Immunohistochemistry further showed EGFP expression within seminiferous tubules, including STRA8-positive germ cells, confirming expression in the premeiotic germ-cell compartment relevant to the present study (Supplementary Fig. S12C). GFP ChIL-seq revealed pronounced H3mm15-EGFP signal accumulation around transcription start sites in transgenic testes relative to wild-type controls, together with a smaller peak around transcription end sites (Supplementary Fig. S12D). Thus, the promoter-proximal distribution observed in NIH3T3 cells was reproduced in developing testicular chromatin, indicating that this feature was not restricted to the fibroblast model. Comparison of genes showing H3mm15-EGFP ChIL-seq enrichment identified 1213 genes shared between testis and NIH3T3 cells, together with 612 testis-restricted and 6659 NIH3T3-restricted genes. The shared genes were enriched for broadly expressed cellular processes, including RNA splicing, intracellular transport, and cell-cycle regulation. In contrast, the testis-restricted gene set was enriched for double-strand break repair and regulation of DNA repair, processes closely associated with meiotic recombination (Supplementary Fig. S12E). H3mm15-EGFP-associated genes showed significantly higher expression than the background of all expressed genes in both testis and NIH3T3 cells (Supplementary Fig. S12F). These findings indicate that H3mm15-EGFP preferentially associates with the transcriptionally active fraction of the genome in both systems, while also occupying testis-specific genes linked to meiotic chromosome regulation.

We then examined whether H3mm15-EGFP expression was associated with changes in H3K4me3 and H3K27me3. We focused on these two modifications because H3K4me3 and H3K27me3 represent promoter-associated states linked to transcriptionally permissive and Polycomb-repressed chromatin, respectively, and because their associations with H3.3 incorporation have previously been demonstrated [17]. ChIL-seq showed broadly similar genome-wide H3K4me3 and H3K27me3 profiles in H3mm15-EGFP- and H3.3-EGFP-expressing cells (Supplementary Fig. S13). Thus, no marked differences in these two histone-modification profiles were detected between the two cell lines. These analyses were limited to H3K4me3 and H3K27me3, and whether H3mm15 affects other histone modifications remains to be determined.

We also focused on H3mm15 incorporation and the histone modification pattern for the serum starvation-response genes detected in Fig. 3 (Fig. 4I and Supplementary Fig. S14). The results showed that, in all clusters of serum starvation-response genes shown in Fig. 3G, a greater proportion of the H3mm15-EGFP signal was assigned to the TSS-proximal region than that of H3.3-EGFP. In addition, the levels of H3K4me3 and H3K27me3 on these genes did not change significantly between H3.3- and H3mm15-overexpressing cells, consistent with our genome-wide results. This suggests that H3mm15 is not incorporated preferentially into specific gene sets, but is broadly enriched at promoter regions, suggesting that H3mm15 potentially contributes to gene expression changes.

### H3mm15-EGFP is associated with moderate chromatin-accessibility changes in response to stimuli

To investigate whether the chromatin accessibility contributes to the relatively modest transcriptional effects associated with H3mm15, we performed an assay for transposase-accessible chromatin using sequencing (ATAC-seq). Because H3mm15-EGFP showed promoter-proximal enrichment and was associated with modest transcriptional changes, we examined how its expression affected chromatin accessibility under steady-state and stimulation conditions. Under steady-state conditions, H3.3-EGFP expression caused significant changes in chromatin accessibility, whereas H3mm15-EGFP expression produced a qualitatively similar but quantitatively smaller effect (Supplementary Fig. S15). This pattern was consistent with the lower nucleosomal incorporation of H3mm15-EGFP indicated by the HAP-IP and ChIL-seq analyses.

To further explore how H3mm15 incorporation affects chromatin accessibility under stimulation conditions, we subjected cells to serum starvation using the same conditions as in Fig. 3. We applied a partial least squares discriminant analysis (PLS-DA) to identify the major variance components distinguishing control and starvation conditions. This was followed by a LDA to establish a classification boundary between these states (Fig. 4J). Based on this model, we defined a “degree of starvation” metric, where negative values correspond to WT-like chromatin accessibility states and positive values indicate starvation-induced changes. To validate the biological relevance of this classification, we performed gene set enrichment analysis (GSEA) on WT serum-starved cells with the highest starvation scores. This revealed a significant decrease in the Normalized Enrichment Score (NES) for cell cycle-related genes (Supplementary Fig. S16), confirming that serum starvation impairs cell cycle progression. Consistently, WT cells exhibited the most pronounced chromatin accessibility changes upon starvation, with the degree of starvation metric shifting from −9.02 (control) to 12.91 (starvation) (Fig. 4K). Furthermore, GO analysis of the differentially accessible genes in WT cells showed that cell cycle-related genes, such as *Cdk4*, showed significant reductions in chromatin accessibility after starvation. This suggests that starvation-induced chromatin condensation contributes to cell cycle arrest (Fig. 4L; Supplementary Figs S17 and S18).

H3.3-EGFP-expressing cells showed an altered basal accessibility state relative to WT cells, with a control degree-of-starvation score of −2.81. H3mm15-EGFP-expressing cells showed a more modest alteration, with a control score of −7.64. Following starvation, the score changed from −2.81 to 3.67 in H3.3-EGFP-expressing cells and from −7.64 to 0.06 in H3mm15-EGFP-expressing cells, compared with the shift from −9.02 to 12.91 in WT cells (Fig. 4K). Thus, expression of either tagged variant attenuated the starvation-associated shift in chromatin accessibility, although the basal effect of H3mm15-EGFP was smaller than that of H3.3-EGFP. This attenuated response was observed among regions associated with both upregulated and downregulated genes. At the Cdk4 locus, for example, the starvation-associated reduction in accessibility was partially mitigated in both H3.3-EGFP- and H3mm15-EGFP-expressing cells (Fig. 4L and Supplementary Fig. S18).

These results indicate that H3mm15-EGFP, like H3.3-EGFP, plays a role in modulating chromatin accessibility dynamics under environmental stimuli such as serum starvation. However, the effect of H3mm15-EGFP on chromatin accessibility and gene expression is more limited, likely because of its lower incorporation efficiency. This suggests that H3mm15 may function to fine-tune chromatin accessibility rather than as a major determinant of chromatin remodeling.

### H3mm15 forms an unstable nucleosome core particle

To investigate the molecular basis of the lower incorporation of H3mm15, we hypothesized that the structural properties of H3mm15-containing nucleosomes may contribute to their functional differences from H3.3-containing nucleosomes. Therefore, we analyzed the structure and stability of H3mm15-containing nucleosomes.

Compared with H3.3, H3mm15 shares serine 31 and the chaperone recognition motif with H3.3, but contains three amino acid differences, at positions 100, 112, and 128 (Fig. 5A). H3mm15 contains glutamine 100 (Q100), valine 112 (V112), and cysteine 128 (C128), corresponding to leucine 100 (L100), isoleucine 112 (I112), and arginine 128 (R128) residues in H3.3. It is still unknown how minor differences in the amino acid composition affect the structure and function of the nucleosome. Therefore, we reconstituted H3mm15-containing nucleosomes *in vitro* and investigated the structure by cryo-electron microscopy (cryo-EM) (Fig. 5B). The workflow for single-particle cryo-EM image processing is summarized in Supplementary Fig. S19. The final cryo-EM reconstruction reached an overall resolution of 2.39 Å, as assessed by Fourier shell correlation, and the local resolution and angular distribution of the particles are shown in Supplementary Fig. S20. The overall structure of the nucleosome core particle (NCP)—consisting of the H2A, H2B, H3mm15, and H4 histones wrapped by 145 bp of DNA—was preserved in the H3mm15 variant. A close-up view of the nucleosome core revealed that the substitutions at positions V112, Q100, and C128 are located at regions critical for histone–histone and histone–DNA interactions. The substitution of Q100 for L100 introduces a polar residue into the hydrophobic core of the histone fold domain. This replacement is likely to disrupt the hydrophobic interactions that stabilize the core of the NCP. The isoleucine-to-valine substitution at position 112 in H3mm15 introduces a shortened side chain, which may affect the stability of the hydrophobic core near the histone–DNA binding site around the nucleosomal dyad. Although valine is still hydrophobic, the shorter side chain is predicted to slightly reduce the hydrophobic interaction in the H3mm15-containing nucleosome. This change may contribute to a more flexible histone–DNA interface around the nucleosomal dyad, potentially facilitating chromatin remodeling processes by increasing DNA accessibility. Additionally, the substitution of arginine to cysteine at position 128 in H3mm15 is particularly notable because this substitutes a basic residue in H3.3 (arginine) with a non-polar residue in H3mm15 (cysteine). This substitution may affect the stability of the hydrophobic interaction near the nucleosomal dyad. Cysteine also contains a sulfhydryl (-SH) group that can participate in a disulfide bond, affecting the NCP structure. Overall, the structural differences introduced by the three amino acid substitutions suggest that H3mm15-containing nucleosomes may be less stable than those containing H3.3.

**Figure 5.**
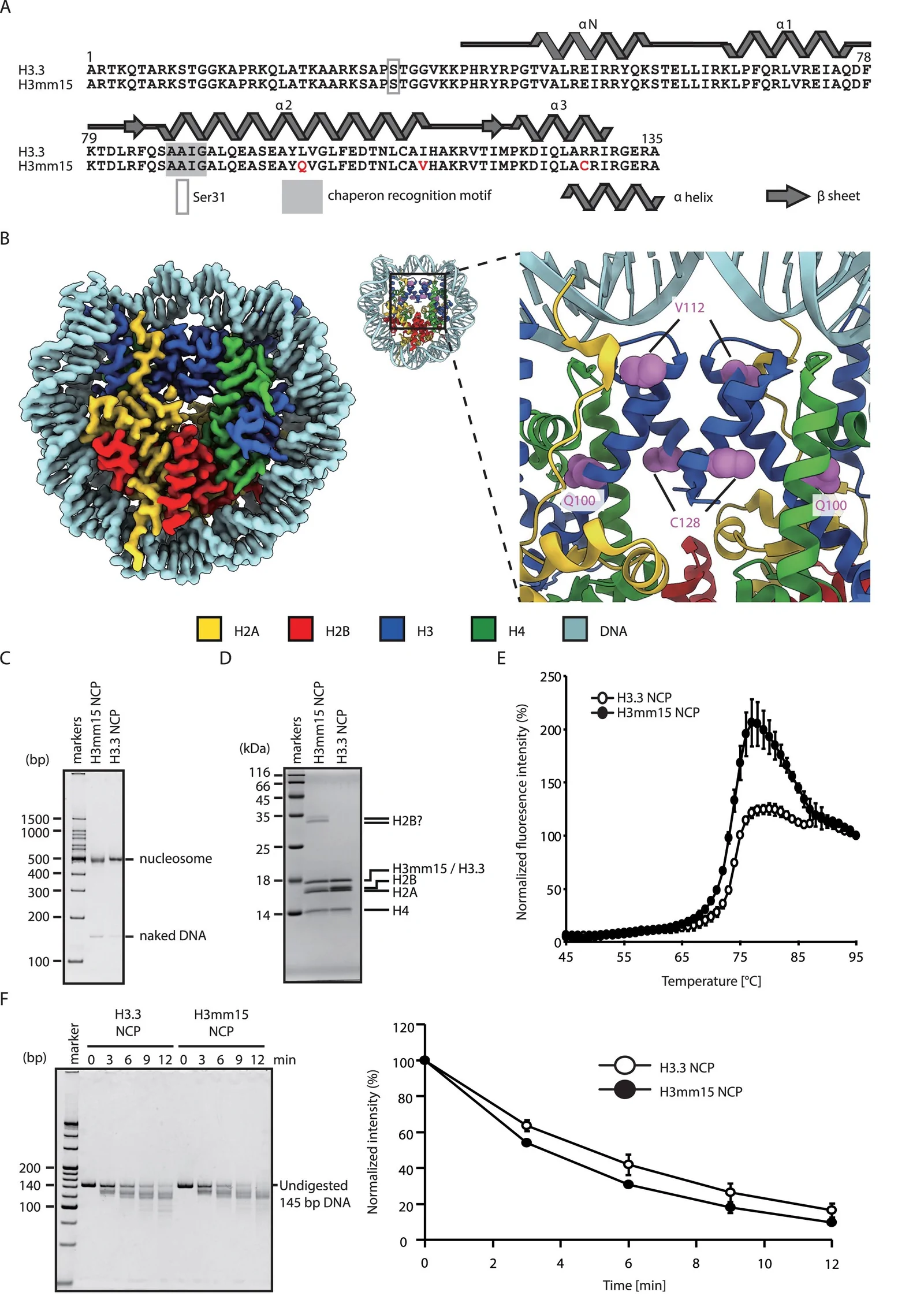
H3mm15 forms a nucleosome that is less stable than the H3.3 nucleosome. (**A**) The amino acid sequence and secondary structure of H3.3 and H3mm15. (**B**) Cryo-EM structure of the NCP containing H3mm15. Cryo-EM density (left); corresponding atomic model (middle); zoomed structure showing the three different amino acids in H3mm15 (Q100, V112, and C128) highlighted. (**C**) H3mm15 and H3.3 NCPs were reconstructed *in vitro* and analyzed by non-denaturing polyacrylamide gel electrophoresis (PAGE). DNA was visualized by ethidium bromide (EtBr), showing that the two NCPs were highly purified, with little naked DNA remaining. (**D**) The NCPs in C were also analyzed by SDS–PAGE, with proteins visualized by Coomassie Brilliant Blue (CBB). The highly purified NCPs contain equimolar quantities of histones. (**E**) Thermal stability assay of NCPs containing H3mm15 or H3.3. Thermal stability assays were performed in three independent experiments. The graph shows the mean ± S.D. of three independent experiments. (**F**) MNase treatment assay of NCPs containing H3mm15 or H3.3.

To reveal the biochemical properties of H3mm15, we purified H3mm15, H3.3, H2A, H2B, and H4 and then reconstituted nucleosomes containing each of the mouse H3 variants (Fig. 5C). Both H3mm15 and H3.3 formed nucleosomes with the core histone ratio of 1:1:1:1 (Fig. 5D). We then tested the stability of the H3mm15 nucleosome using a thermal stability assay (TSA), as previously reported [9, 10] (Fig. 5E). For the H3.3 NCP, the first peak of the fluorescence intensity curve reflected the dissociation of H2A–H2B, and the second peak reflected the dissociation of H3–H4 [65]. However, for the H3mm15 NCP, the boundary between the first and second peaks disappeared, suggesting that the H2A–H2B dimer and the H3mm15–H4 tetramer dissociate simultaneously, at a lower temperature than the H3.3 NCP.

We also performed a micrococcal nuclease (MNase) assay, as previously reported [9, 10] (Fig. 5F). MNase preferentially digests DNA end regions, but does not interact with DNA that is tightly bound to histones. This showed that, compared with the H3.3 NCP, the DNA ends of the H3mm15 NCP were more susceptible to MNase. This difference was consistently observed in independent replicate experiments (Supplementary Fig. S21). This indicates that the DNA–histone interactions at the entry/exit regions of the H3mm15 nucleosome are weaker than in the H3.3 nucleosome, and thus that H3mm15 forms a less stable NCP than H3.3.

### Q100 is a major determinant of the dynamic chromatin association of H3mm15 in cells

To determine whether the three H3mm15-specific amino acid residues contribute to its chromatin properties in cells, we generated NIH3T3 cell lines expressing EGFP-tagged H3mm15 single-reversion mutants, in which each H3mm15-specific residue was replaced with the corresponding residue in H3.3. These mutants were designated Q100L, V112I, and C128R. All three mutant proteins were predominantly localized in the nucleus (Fig. 6A). Among them, Q100L showed more clearly defined nuclear enrichment than V112I and C128R. We next evaluated their chromatin dynamics by FRAP. H3mm15-EGFP showed rapid and extensive FRAP, whereas H3.3-EGFP showed only limited recovery, consistent with the more stable chromatin association of H3.3 (Fig. 6B). Notably, Q100L-EGFP showed markedly reduced fluorescence recovery, with a recovery profile resembling that of H3.3-EGFP. By contrast, V112I-EGFP and C128R-EGFP exhibited more extensive fluorescence recovery, resembling H3mm15-EGFP. The FRAP analysis included H3.3-EGFP (*n* = 59 cells), H3mm15-EGFP (*n* = 60 cells), Q100L-EGFP (*n* = 90 cells), V112I-EGFP (*n* = 30 cells), and C128R-EGFP (*n* = 30 cells). These results identify Q100 as a major determinant of the dynamic chromatin association of H3mm15 in NIH3T3 cells.

**Figure 6.**
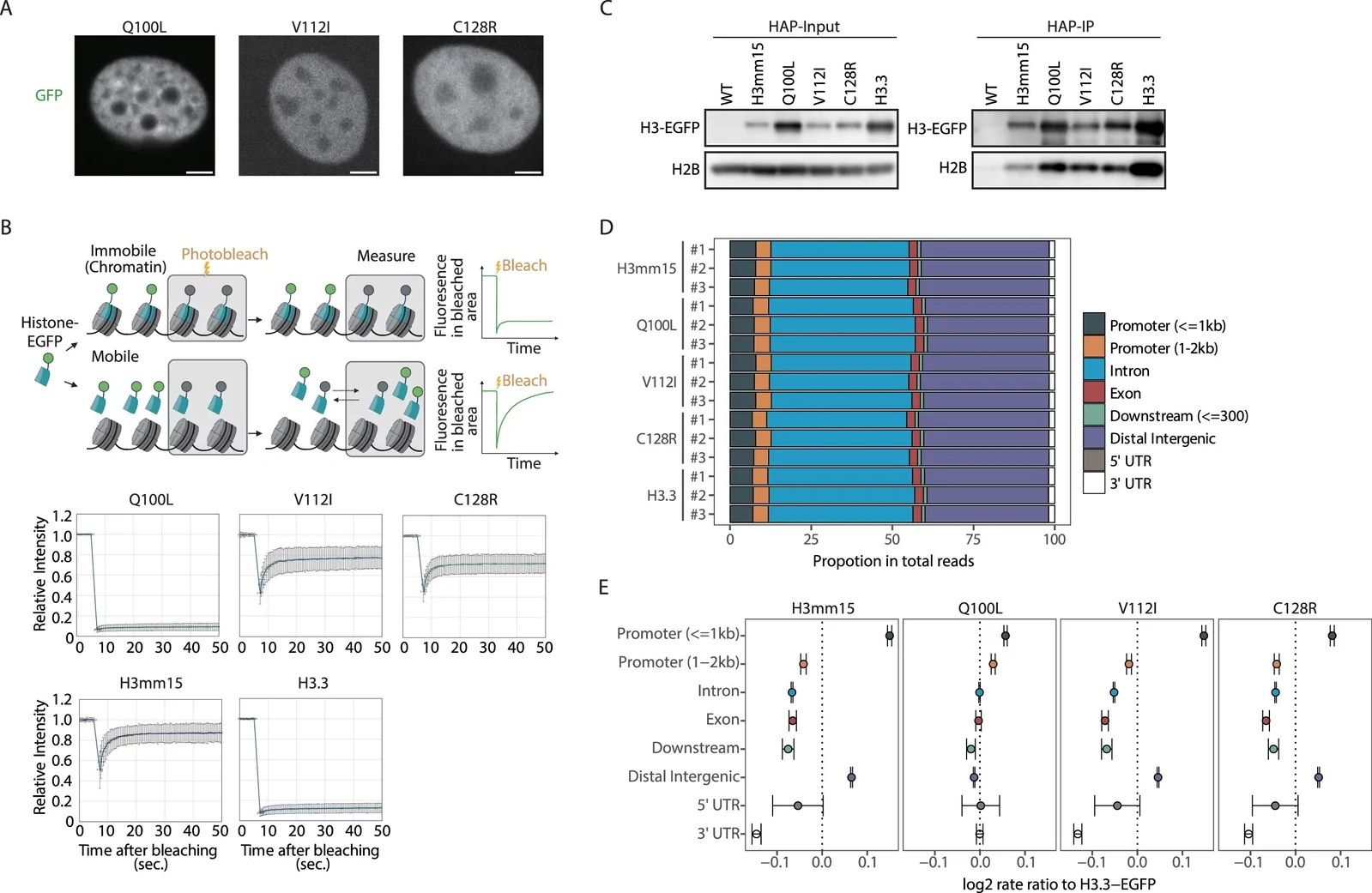
Q100 is a major determinant of the dynamic chromatin association of H3mm15 in cells. (**A**) Fluorescence images of NIH3T3 cells expressing EGFP-tagged H3mm15 single-reversion mutants Q100L, V112I, and C128R. Each H3mm15-specific residue was replaced with the corresponding residue in H3.3. All three mutants were predominantly localized in the nucleus, and Q100L showed particularly clear nuclear enrichment. Representative images from three independent cell preparations are shown; scale bars: 5 µm. (**B**) FRAP analysis of H3.3-EGFP, H3mm15-EGFP, and the indicated H3mm15 single-reversion mutants. A 2-µm-diameter region within the nucleoplasm was photobleached, and fluorescence recovery was measured over time. Curves represent the mean, and shaded regions indicate ± S.D. H3.3-EGFP, *n* = 59 cells; H3mm15-EGFP, *n* = 60 cells; Q100L-EGFP, *n* = 90 cells; V112I-EGFP, *n* = 30 cells; and C128R-EGFP, *n* = 30 cells. Created in BioRender. Harada, A. (2026) https://BioRender.com/m38kad9. (**C**) Immunoblotting of hydroxyapatite-purified nucleosomes before immunoprecipitation (HAP-input) and after immunoprecipitation with an anti-GFP antibody (HAP-IP). EGFP-tagged histone variants and H2B were detected using anti-GFP and anti-H2B antibodies, respectively. Representative immunoblots from two independent HAP/HAP-IP experiments are shown. (**D**) Genomic distributions of H3mm15-EGFP, H3.3-EGFP, and the indicated H3mm15 mutants determined by ChIL-seq. The proportions of reads assigned to promoters, introns, exons, downstream regions, untranslated regions, and distal intergenic regions are shown. Three replicates are presented for each histone variant. (**E**) Log2 ratios of the proportions of reads assigned to each genomic feature for H3mm15-EGFP and the indicated mutants relative to H3.3-EGFP. Points represent estimated log2 rate ratios, and error bars indicate 95% confidence intervals.

We next examined the association of the mutant proteins with nucleosomal chromatin by hydroxyapatite purification followed by immunoprecipitation with an anti-GFP antibody. Q100L-EGFP showed a stronger signal in the HAP-IP fraction than H3mm15-EGFP, together with the recovery of H2B, consistent with its reduced mobility in the FRAP analysis (Fig. 6C). The effects of the V112I and C128R substitutions were less pronounced. Because the abundance of H3-EGFP differed among the HAP-input fractions, however, these immunoblotting results were interpreted qualitatively rather than as a quantitative comparison of nucleosomal incorporation efficiencies.

ChIL-seq analysis showed that the overall genomic-feature distributions of H3mm15, Q100L, V112I, and C128R were broadly similar across biological replicates (Fig. 6D). Comparison with H3.3-EGFP revealed modest differences in the relative representation of individual genomic features, including promoter regions, but none of the single-reversion mutants showed a substantial redistribution among broad genomic features (Fig. 6E). Thus, the amino acid substitutions primarily affected chromatin retention rather than the overall genomic-feature distribution of H3mm15.

We also examined whether the EGFP-tagged H3 proteins incorporated into chromatin could acquire histone modifications. Immunoblotting of HAP-purified nucleosomal fractions detected H3K4me3-reactive signals at the expected molecular weight of H3-EGFP, most clearly for H3.3-EGFP and Q100L-EGFP (Supplementary Fig. S22). The H3K4me3 signals broadly paralleled the abundance of the corresponding H3-EGFP proteins in the HAP fractions. These results indicate that chromatin-associated H3-EGFP proteins can acquire H3K4me3 but do not establish variant-specific differences in modification efficiency.

Together, these findings indicate that Q100 strongly influences the chromatin retention and nucleosomal association of H3mm15, while having a limited effect on its overall genomic-feature distribution.

### Reduced stability of the H3mm15 nucleosome does not facilitate RNAPII passage *in vitro*

To directly test whether the reduced stability of the H3mm15 nucleosome facilitates transcription through chromatin, we performed a purified *in vitro* nucleosomal transcription elongation assay. H3.3- or H3mm15-containing nucleosomes were reconstituted on a 153-bp DNA fragment containing a modified Widom 601 sequence. The nucleosomal DNA was subsequently ligated to a 45-bp DNA fragment containing a 9-bp mismatch bubble, yielding a 198-bp transcription template (Fig. 7A). The integrity of the reconstituted nucleosomes and ligated transcription templates was confirmed by SDS–PAGE and native PAGE (Supplementary Fig. S23A).

**Figure 7.**
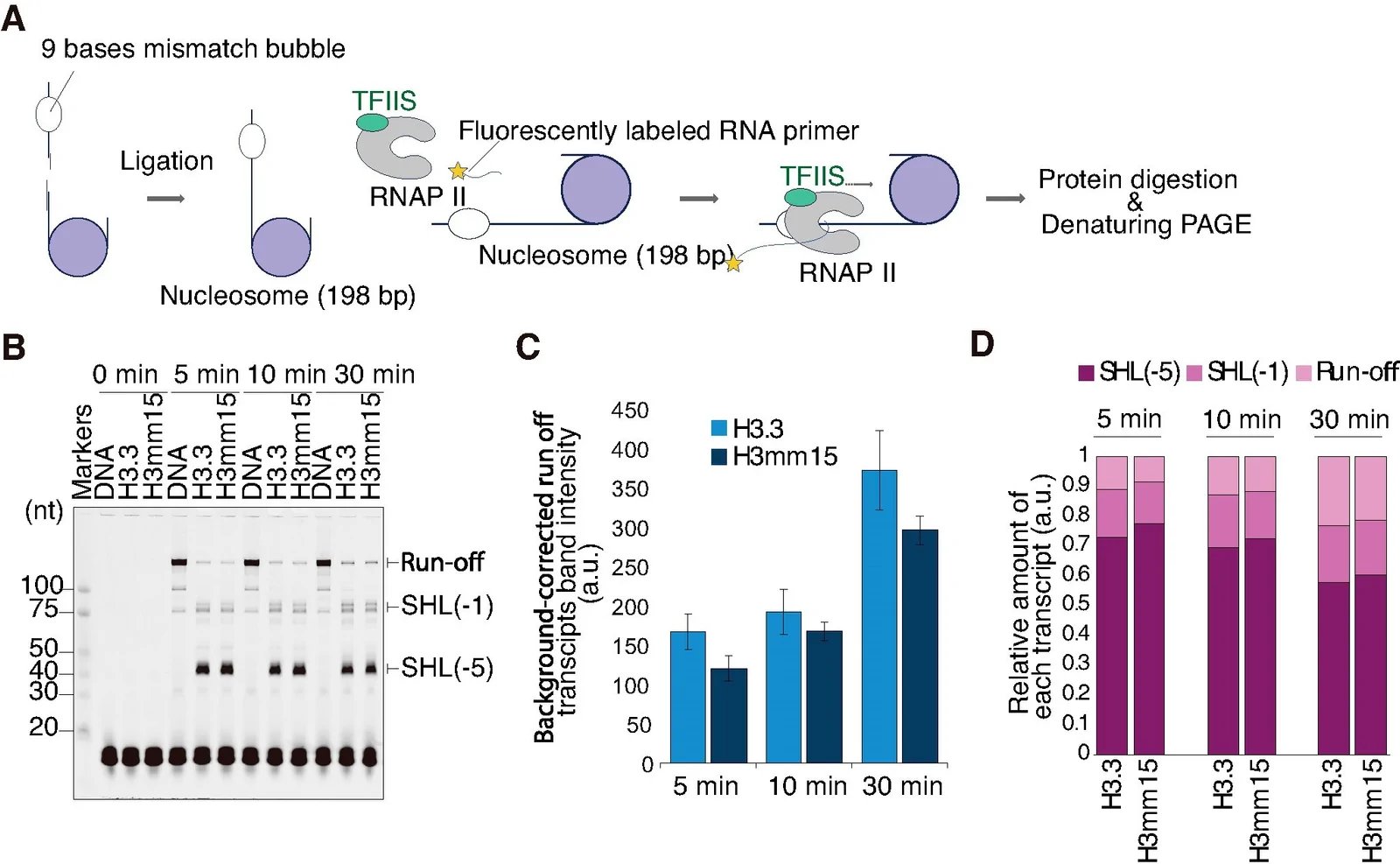
*In vitro* transcription through H3.3- and H3mm15-containing nucleosomes. (**A**) Schematic representation of the *in vitro* nucleosomal transcription assay. H3.3- or H3mm15-containing nucleosomes reconstituted on a modified Widom 601 DNA fragment were ligated to a 45-bp DNA fragment containing a 9-bp mismatch bubble. Transcription was performed using *Komagataella pastoris* RNAPII and TFIIS. After protein digestion, DY647-labeled RNA products were analyzed by denaturing PAGE. (**B**) Representative *in vitro* transcription assay using naked DNA and nucleosomes containing either H3.3 or H3mm15. Reactions were performed for 0, 5, 10, or 30 min. The positions of the SHL(−5)-stalled, SHL(−1)-stalled, and run-off transcripts are indicated. The results of all three independent experiments are shown in Supplementary Fig. S23B. (**C**) Quantification of run-off RNA transcripts. Background-subtracted run-off band intensities are shown in arbitrary units. Data represent the mean ± s.d. of three independent transcription assays. Statistical significance was assessed using a two-sided unpaired Student’s *t*-test. *P* = 0.043, 0.240, and 0.071 for 5, 10, and 30 min, respectively. (**D**) Relative proportions of the SHL(−5)-stalled, SHL(−1)-stalled, and run-off transcripts. The background-subtracted intensity of each RNA product was divided by the combined intensity of the three products at each time point. Bars represent the mean proportions obtained from three independent transcription assays.

Transcription reactions were performed using purified *Komagataella pastoris* RNAPII and TFIIS, and the RNA products were analyzed after 0, 5, 10, and 30 min. Three independent transcription experiments were performed (Supplementary Fig. S23B). Run-off transcripts accumulated over time on both the H3.3 and H3mm15 nucleosomal templates (Fig. 7B). However, the amount of run-off transcript was consistently lower on the H3mm15 template than on the H3.3 template. This difference reached statistical significance at 5 min but not at 10 or 30 min (Fig. 7C). The relative proportions of the SHL(−5)-stalled, SHL(−1)-stalled, and run-off transcripts showed no clear difference between the H3.3 and H3mm15 nucleosomal templates (Fig. 7D). These results indicate that the reduced stability of the H3mm15 nucleosome does not facilitate RNAPII passage under these defined *in vitro* conditions. Instead, H3mm15 modestly reduced run-off transcript production at the early time point without substantially altering the major RNAPII-stalled product profile. Thus, these findings argue against a simple model in which the lower intrinsic stability of the H3mm15 nucleosome directly enhances transcription through chromatin.

## Discussion

Histone variants are key players in the epigenetic regulation of gene expression and often confer specific functional characteristics to nucleosomes, thereby affecting chromatin dynamics and cellular processes. The histone variant H3mm15 is predominantly expressed in the testis and has unique characteristics distinguishing it from canonical histones and other H3 variants such as H3.3. Loss of H3mm15 results in disordered gene expression during male meiosis, suggesting its potential roles in chromatin structural regulation and gene expression, which are important for normal spermatogenesis. The relatively mild phenotype of H3mm15-deficient mice may reflect partial functional redundancy with H3.3 or other H3.3 subvariants. However, the transcriptional regulation of H3mm15 itself remains unclear. While our expression analyses suggest testis-enriched transcription, it is still undetermined whether this is mediated by testis-specific promoters or by tissue-restricted transcription factors. Dissecting the upstream regulatory logic of H3mm15 will be essential to understanding its developmental specificity and functional timing during spermatogenesis.

Previous studies on histone variants such as H3.3 have demonstrated their critical roles in maintaining active chromatin regions and regulating gene expression during development. H3.3 is enriched in transcriptionally active regions and is essential for the proper execution of developmental programs across various tissues [66]. However, our results show that H3.3 overexpression buffers the chromatin accessibility changes resulting from serum starvation treatment. This is consistent with previous reports, which demonstrated that overexpressed H3.3 inhibited chromatin accessibility changes in B cells and prevented differentiation into plasmacytes [67]. This study shows that H3mm15 is relatively enriched at promoter-proximal regions compared with H3.3, suggesting its potential role in gene expression changes. While the ChIL-seq results suggest promoter-preferential localization of H3mm15, the overall genomic distribution patterns are broadly similar to H3.3, including signals in distal intergenic regions. The promoter-proximal distribution of H3mm15-EGFP was also reproduced in transgenic mouse testes, indicating that this feature is not restricted to NIH3T3 fibroblasts. However, because the transgene was driven by the CAG promoter and whole testes were used for ChIL-seq, these results do not establish the genomic distribution of endogenous H3mm15 in specific spermatogenic cell populations. Nevertheless, the increased density of H3mm15 signals near transcription start sites under our controlled overexpression system supports its enrichment in regulatory regions. While H3.3 and H3mm15 overexpression both exhibited a buffering effect on chromatin accessibility changes under stimulation conditions, such as serum starvation, H3mm15-overexpressing cells had less of an impact on these gene expression changes than H3.3-overexpressing cells. We propose that this difference arises from the lower incorporation level of H3mm15, which may limit its ability to effectively regulate chromatin structure and gene expression compared with H3.3. H3mm15-EGFP-associated genes were biased toward higher expression in both NIH3T3 cells and transgenic testes, suggesting an association with transcriptionally active chromatin. However, it remains unresolved whether this reflects selective targeting of H3mm15 or its preferential incorporation into active loci with rapid histone turnover.

While NIH3T3 fibroblasts do not recapitulate the chromatin environment of germ cells, endogenous H3mm15 transcripts were detectable in these cells, although at a lower level than in whole testis. Thus, the NIH3T3 model serves as a useful system to reveal intrinsic functional distinctions between H3.3 subvariants in a controlled perturbation context, rather than to replicate testicular chromatin states. Combined with RNA-seq data showing more modest gene expression responses than H3.3 under serum starvation, these results suggest that H3mm15 has inherently limited effects on transcriptional responses, despite occupying similar promoter regions. This apparent paradox may reflect the limited chromatin incorporation of H3mm15 or its preferential association with actively transcribed, high-turnover loci. Alternatively, H3mm15 incorporation may contribute to context-dependent modulation of gene expression in a stimulus-specific or stage-specific manner rather than directly enhancing basal transcriptional output.

There are two hypotheses for the low incorporation of H3mm15: it may either be difficult to incorporate, or it may be quickly removed from chromatin after incorporation. To investigate this, we examined the structure of H3mm15-containing NCPs. While H3mm15 could form NCPs *in vitro*, these structures had reduced thermal stability and increased sensitivity to nuclease digestion, indicating that H3mm15 forms a biochemically less stable nucleosome than H3.3. However, the *in vitro* transcription assay showed that this instability did not result in enhanced RNAPII transcription. Run-off transcript production and the distributions of the major RNAPII-stalled products were broadly comparable between H3mm15- and H3.3-containing nucleosomes. Thus, reduced nucleosome stability and increased accessibility of the DNA entry/exit regions do not necessarily correspond to a lower transcriptional barrier for RNAPII. Because the assay measured RNA synthesis rather than histone retention, whether H3mm15-containing nucleosomes are physically removed more rapidly during transcription remains unresolved.

Cell-based analysis of the H3mm15 single-reversion mutants further showed that the three H3mm15-specific amino acid residues do not contribute equally to its chromatin properties. Among the Q100L, V112I, and C128R mutants, Q100L showed the most pronounced reduction in FRAP recovery and increased abundance in the nucleosomal fraction. These findings indicate that Q100 is a major determinant of the dynamic chromatin association and limited chromatin retention of H3mm15. The substitution of leucine in H3.3 with the polar glutamine residue in H3mm15 is likely to weaken hydrophobic packing within the histone-fold domain, consistent with the structural and biochemical properties of the H3mm15 nucleosome.

Our chaperone depletion experiments also provide insight into the limited incorporation of H3mm15. Whereas H3.3-EGFP incorporation was sensitive to HIRA depletion, H3mm15-EGFP incorporation was more strongly affected by DAXX depletion. DAXX depletion reduced H3mm15 signals mainly in distal and gene-body regions, whereas its relative promoter-proximal enrichment was maintained. These findings suggest that DAXX contributes to a portion of H3mm15 deposition but is not solely responsible for its promoter-proximal incorporation. The identity of the factor or pathway mediating promoter-proximal H3mm15 deposition remains unclear.

The instability of H3mm15-containing nucleosomes is characteristic of several testis associated histone variants, including H3t, Th2a, Th2b, H2A.Z, H3T, and H3.5 [13, 68–71], which facilitate dynamic chromatin remodeling during male germ cell development. The possibility that H3mm15 is involved in such dynamic processes in the testis warrants further studies, particularly in the context of its interactions with other chromatin-associated factors. Nevertheless, the transcription assay and the relatively modest effects observed in NIH3T3 cells indicate that H3mm15 is not a generally transcription-permissive histone variant. Instead, its limited incorporation and distinct nucleosomal properties may contribute to context-dependent modulation of transcriptional responses.

One limitation of this study is the lack of an H3mm15-specific antibody, preventing direct localization in testis tissue. In addition, although whole-testis mRNA-seq detected no transcriptional upregulation of other H3.3-type variants in *H3mm15*^−/−^ testes, this analysis cannot exclude compensatory changes restricted to particular spermatogenic cell populations, or compensation occurring through constitutive functional redundancy, competition for histone chaperones, or altered protein stability and chromatin deposition without accompanying changes in mRNA abundance. Although NIH3T3 overexpression models and transgenic testes have provided insights, spermatogenesis is highly specialized, and the present systems do not resolve the genomic distribution of endogenous H3mm15 in individual spermatogenic stages. In addition, because EGFP fusion may affect histone interactions or genomic localization [72], the chromatin-profiling results should be interpreted as relative comparisons between similarly tagged H3.3 and H3mm15 rather than as direct measurements of endogenous H3mm15 localization. The present HAP-IP analysis also did not distinguish homotypic tagged nucleosomes from heterotypic nucleosomes containing endogenous H3. Future studies employing endogenous tagging or H3mm15-EGFP knock-in mice will therefore be essential to validate its *in vivo* localization and chromatin targets. Furthermore, generation of a knock-in model in which the H3mm15 CDS is replaced by that of H3f3a would directly determine the physiological importance of the H3mm15-specific amino acid substitutions.

In conclusion, H3mm15 represents a distinct histone variant that contributes to chromatin dynamics in spermatogenesis. Its limited chromatin incorporation, differential chaperone sensitivity, and structurally unstable nucleosome properties are consistent with a context-dependent effect on transcriptional responses rather than a general effect on transcriptional output. Its role in maintaining proper gene expression underscores the functional diversity of histone variants and their impact on tissue-specific gene regulation. Future studies will be essential to reveal the precise molecular mechanisms by which H3mm15 and other histone variants affect chromatin dynamics and gene expression in spermatogenic cells.

## Supplementary Material

gkag994_Supplemental_File

## Data Availability

Plasmids generated from this study are available from the corresponding author upon request. Next-generation sequencing datasets generated during this study have been deposited to the Gene Expression Omnibus under accession number GSE290629, GSE290630, GSE290631, GSE290632, GSE290633, GSE290634, GSE341161, GSE341158, GSE341157, and GSE341156. Atomic coordinates have been deposited in the PDB at https://doi.org/10.2210/pdb9mb2/pdb, and cryo-EM maps have been deposited in the EMDB under accession number EMD-63765. Uncropped blots (Supplementary Figs S24–S26) and replicates of the MNase assay (Supplementary Fig. S21) are included in the Supplementary data.
