# Supplementary material for: Histone H3.3 subvariant H3mm15 is required for normal spermatogenesis by regulating gene expression
Source: Nucleic Acids Res. 2026 Nov 9;54(20):gkag994. doi: 10.1093/nar/gkag994 (PMC13744785; doi:10.1093/nar/gkag994)
Supplement: Supplementary file 1 [file gkag994_supplemental_file.pdf]

Supplementary Materials for

Histone H3.3 subvariant H3mm15 is required for normal spermatogenesis by regulating gene expression

Qianmei Wu *et al.*

This PDF file includes:

Supplementary Figures S1 to S26

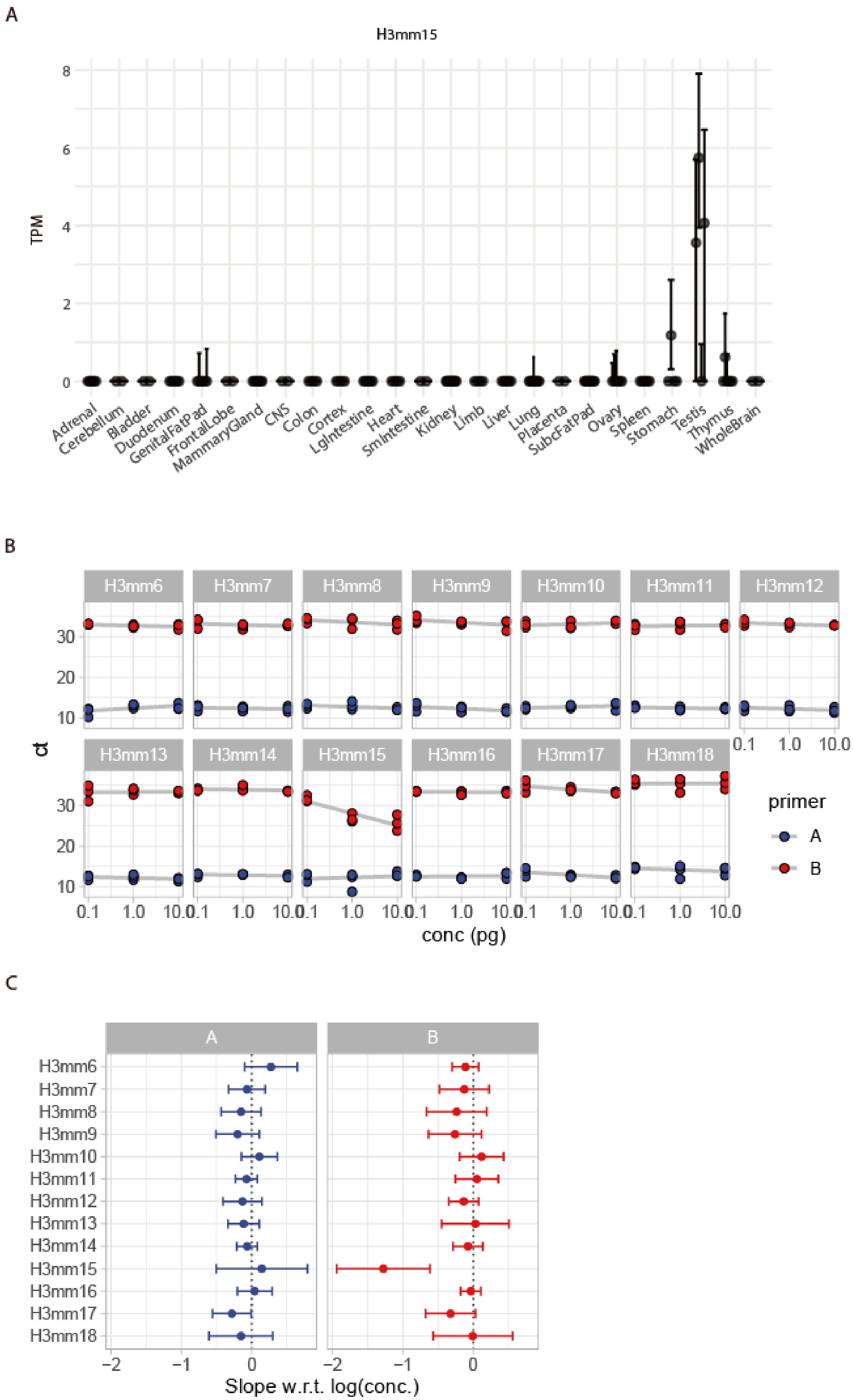
A
H3mm15
TPM
0
2
4
6
8
Adrenal
Cerebellum
Bladder
Duodenum
GenitalFatPad
FrontalLobe
MammaryGland
CNS
Colon
Cortex
LgIntestine
Heart
SmIntestine
Kidney
Limb
Liver
Lung
Placenta
SubcFatPad
Ovary
Spleen
Stomach
Testis
Thymus
WholeBrain
B
H3mm6
H3mm7
H3mm8
H3mm9
H3mm10
H3mm11
H3mm12
H3mm13
H3mm14
H3mm15
H3mm16
H3mm17
H3mm18
ct
10
20
30
0.1
1.0
10.0
conc (pg)
primer
A
B
C
A
B
H3mm6
H3mm7
H3mm8
H3mm9
H3mm10
H3mm11
H3mm12
H3mm13
H3mm14
H3mm15
H3mm16
H3mm17
H3mm18
−2
−1
0
Slope w.r.t. log(conc.)

Supplementary Figure S1.

Analysis and assay validation of mouse *H3mm15* expression. (A) Expression levels of the mouse *H3mm15* gene across 25 tissues based on Cold Spring Harbor Laboratory (CSHL) RNA sequencing data. The horizontal axis represents the tissues, and the vertical axis represents transcripts per million (TPM). **(B)** To evaluate the variant specificity and quantitative capacity of the variant-specific RT-qPCR assay for H3mm15, a constant amount of H3f3a plasmid (10 ng) was mixed with varying concentrations (0.1, 1.0, and 10.0 pg) of plasmids encoding each of the 13 mouse H3.3 subvariants (H3mm6–H3mm18). Real-time PCR was performed using two primer conditions: primer set A (blue), designed to amplify all H3f3a-type variants, and primer set B (red), which includes an H3mm15-specific forward primer and a blocking oligonucleotide that inhibits amplification of other variants. Ct values were plotted against input plasmid concentrations for each variant. Each dot represents an individual measurement, and gray lines represent regression curves based on the model $Ct = a + b \cdot \log_{10}[\text{concentration}]$. A clear dose-dependent signal was observed for H3mm15 only using primer set B, confirming the quantitative and variant-specific nature of the variant-specific RT-qPCR assay. Each point represents one technical replicate. Three technical replicates were analyzed for each plasmid concentration in one representative experiment. **(C)** Plot showing slope coefficients from the linear regression for each histone variant. Error bars represent 95% confidence intervals. Dashed line indicates slope = 0 (no concentration dependence).

A

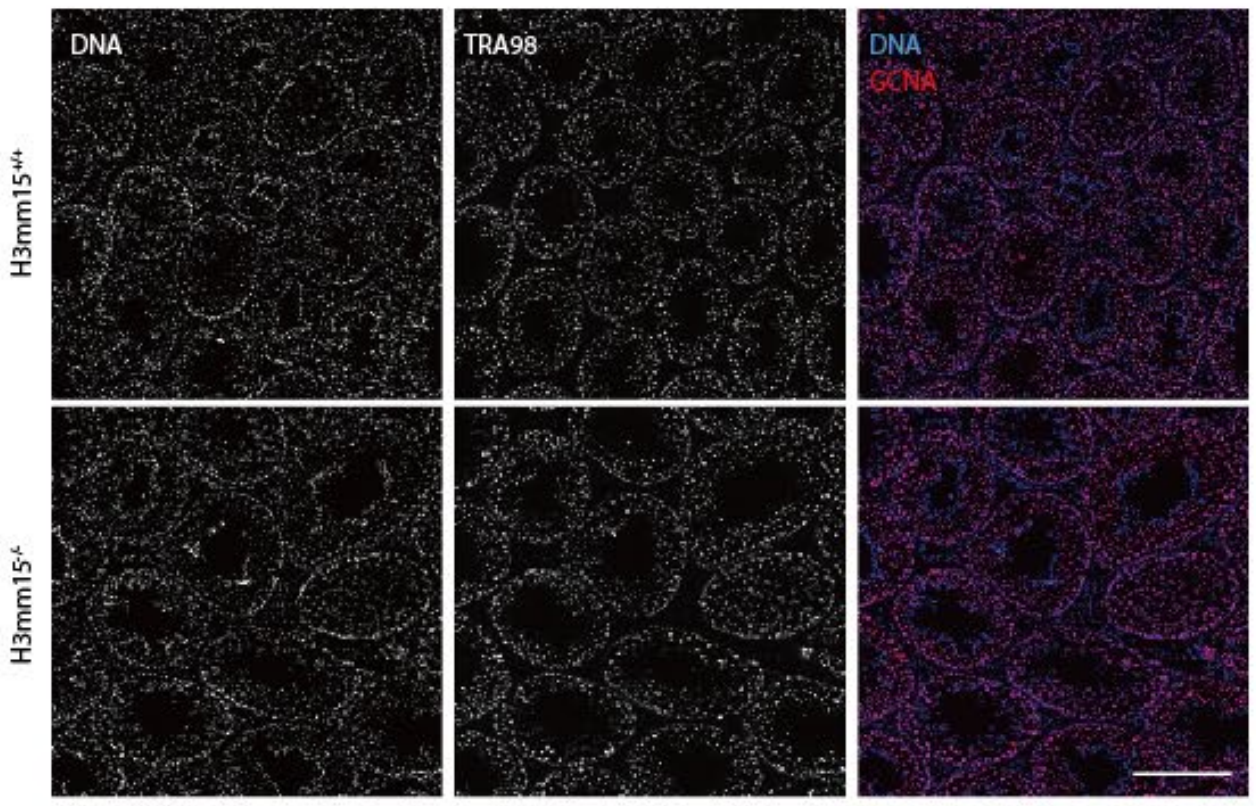
DNA
TRA98
DNA
GCNA
H3mm15+/+
H3mm15-/-

B

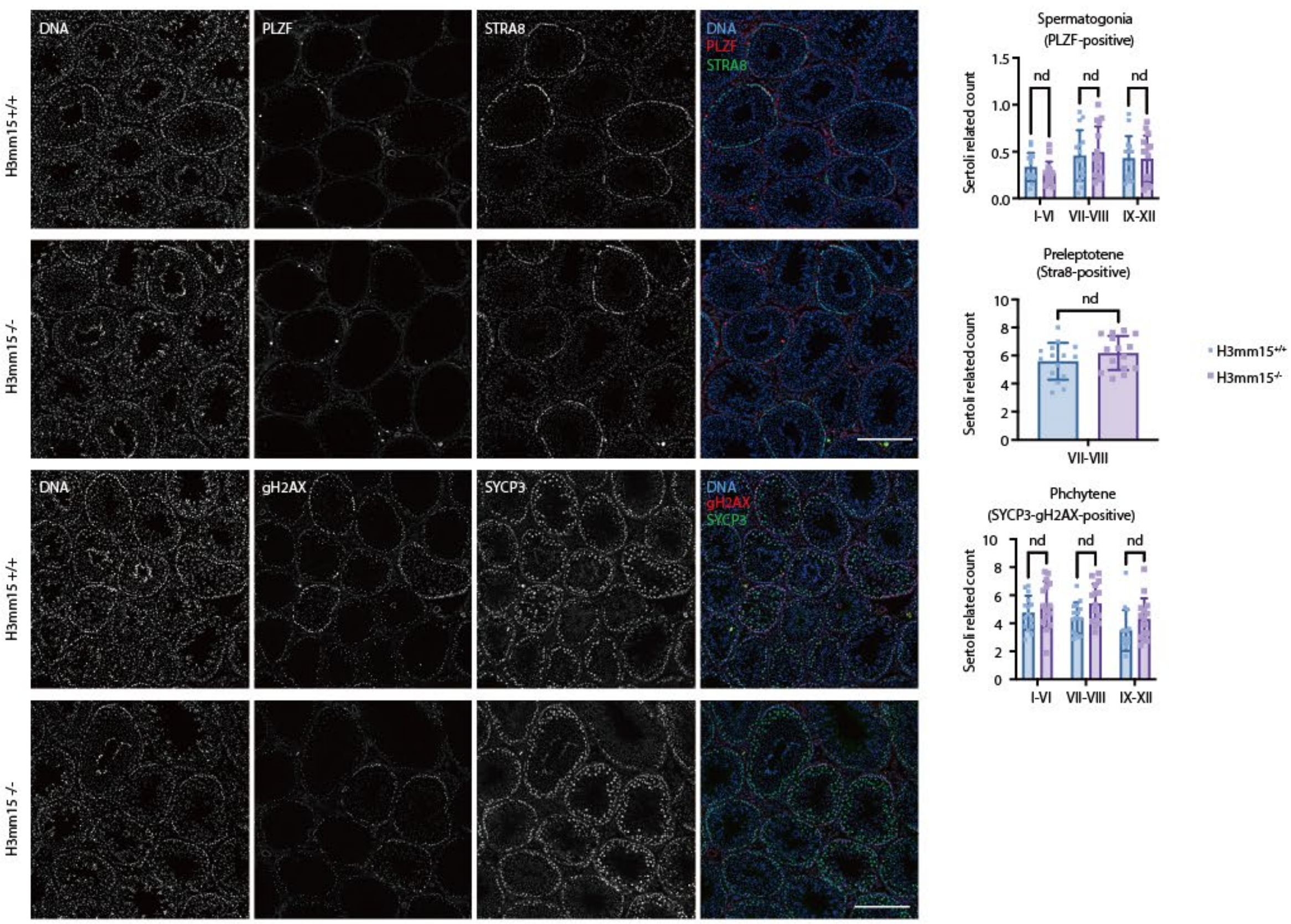
DNA
PLZF
STRA8
DNA
PLZF
STRA8
H3mm15+/+
H3mm15-/-
DNA
gH2AX
SYCP3
DNA
gH2AX
SYCP3
H3mm15+/+
H3mm15-/-
Spermatogonia
(PLZF-positive)
Sertoli related count
nd
I-VI
VII-VIII
IX-XII
Preleptotene
(Stra8-positive)
Sertoli related count
nd
VII-VIII
H3mm15+/+
H3mm15-/-
Phchytene
(SYCP3-gH2AX-positive)
Sertoli related count
nd
I-VI
VII-VIII
IX-XII

C

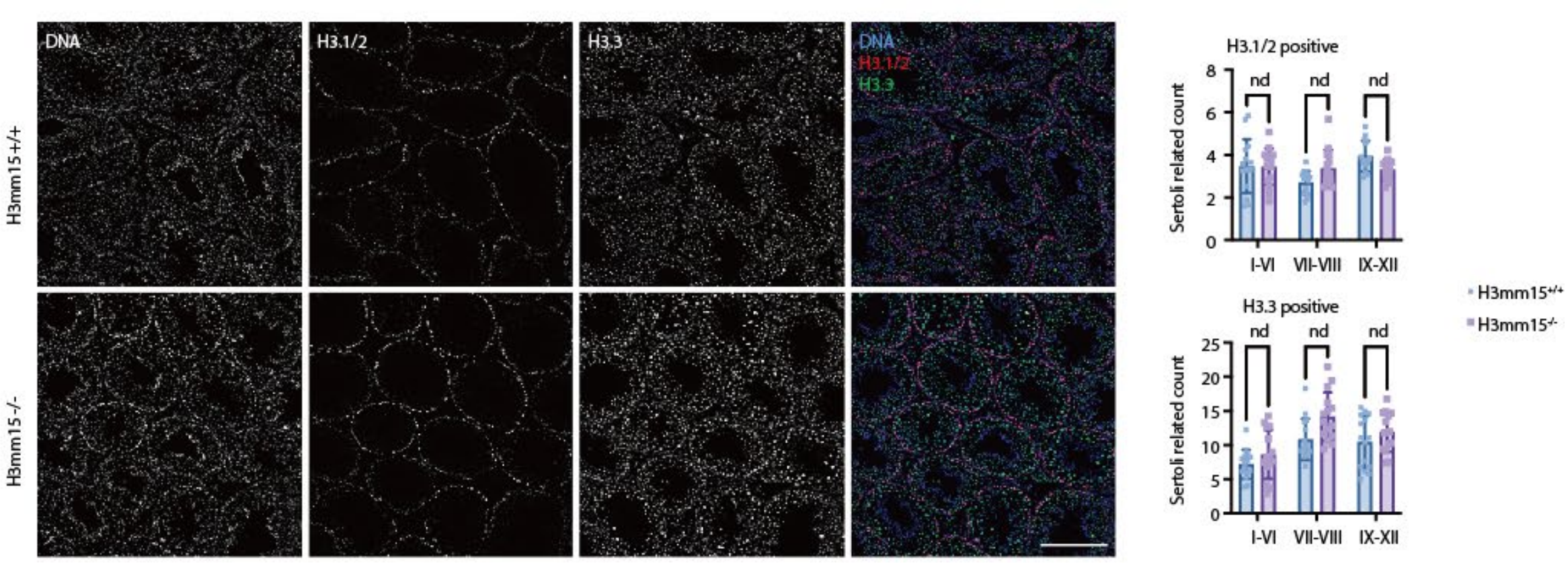
DNA
H3.1/2
H3.3
DNA
H3.1/2
H3.3
H3mm15+/+
H3mm15-/-
H3.1/2 positive
Sertoli related count
nd
I-VI
VII-VIII
IX-XII
H3mm15+/+
H3mm15-/-
H3.3 positive
Sertoli related count
nd
I-VI
VII-VIII
IX-XII

Supplementary Figure S2.

Spermatogenic cell marker and histone immunostaining of *H3mm15*$^{+/+}$ and *H3mm15*$^{-/-}$ testis sections. **(A)** Immunostaining of *H3mm15*$^{+/+}$ and *H3mm15*$^{-/-}$ testis sections with the germ cell marker GCNA. **(B)** Immunostaining of different cell types with specific markers in *H3mm15*$^{+/+}$ and *H3mm15*$^{-/-}$ testis sections. PLZF, spermatogonia specific marker; STRA8, preleptotene specific marker; gH2AX and SYCP3, spermatocyte markers. To ensure better targeting of spermatogenic cells at different phases, we divided the seminiferous tubules into three groups (I–VI, VII–VIII, and IX–XII) according to their stages, and quantified the cells in different phases. The numbers of Sertoli cells and each type of spermatogenic cell were counted. "Sertoli-related counts" were calculated by target cell number/Sertoli cell number. The vertical axis represents the Sertoli-related counts and the horizontal axis represents the stages of the seminiferous tubules. Multiple pairwise comparisons were performed using two-sample t-tests to evaluate the differences between groups. Given the potential for type I errors because of multiple testing, the false discovery rate (FDR) was controlled using the two-stage step-up method of Benjamini, Krieger, and Yekutieli, specifically designed to maintain control at a strict FDR of 1%. nd, non-detectable. **(C)** H3.1/2 and H3.3 immunostaining of *H3mm15*$^{+/+}$ and *H3mm15*$^{-/-}$ testis sections. The same cell count analysis was performed as in Supplementary Figure S2B. Scale bar, 200 µm.

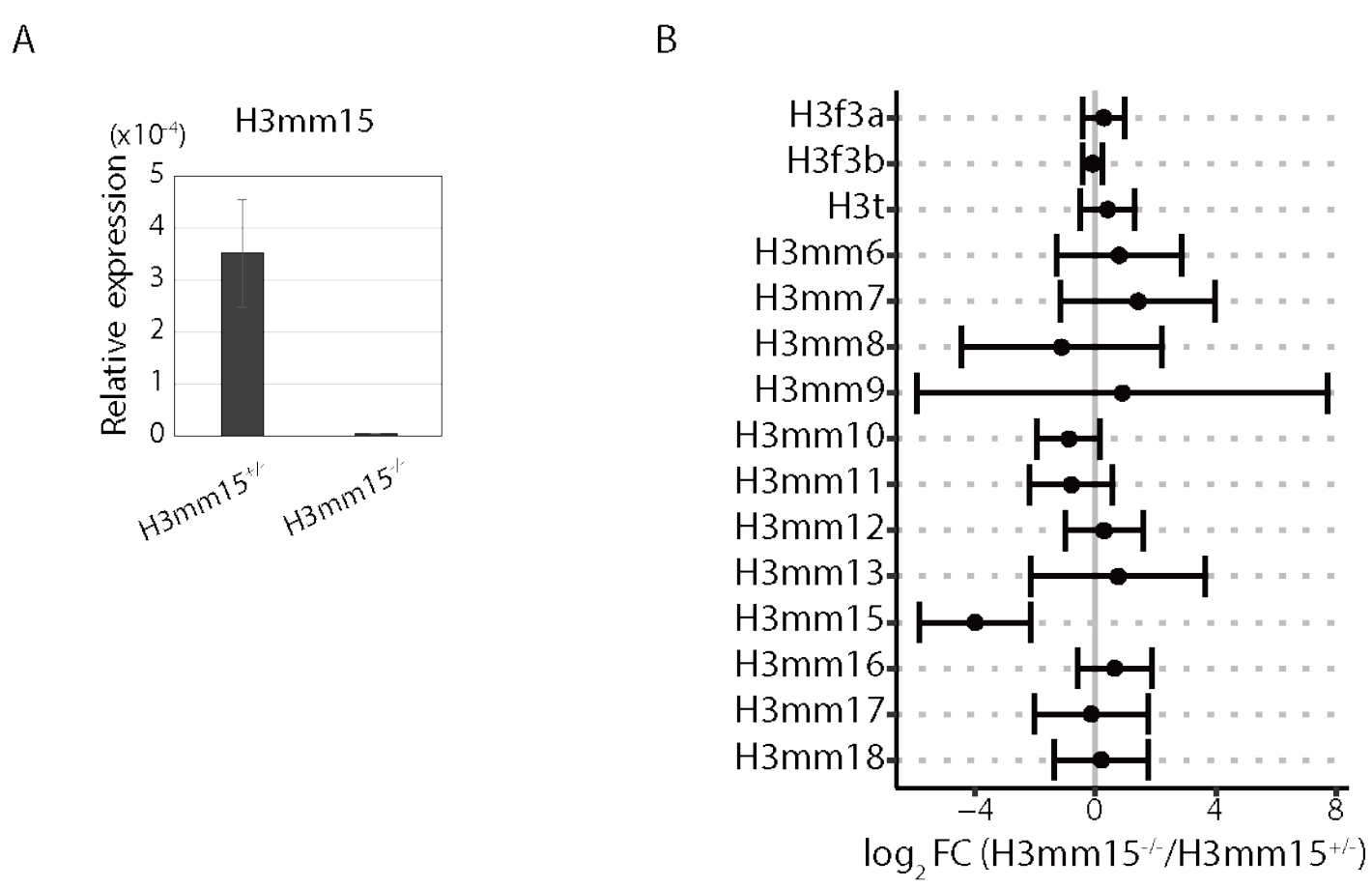

Supplementary Figure S3.

Expression of H3.3-type histone variants in H3mm15-deficient testes. **(A)** Variant-specific RT-qPCR analysis of H3mm15 expression in testes from *H3mm15*$^{+/-}$ littermate controls (n = 4) and *H3mm15*$^{-/-}$ mice (n = 3). Data points represent individual mice. Data represent the mean ± S.D. **(B)** Differential expression of H3.3-type histone variants determined by whole-testis RNA-seq. Points indicate estimated log2 fold changes for *H3mm15*$^{-/-}$ relative to *H3mm15*$^{+/-}$ testes, and horizontal error bars indicate 95% confidence intervals.

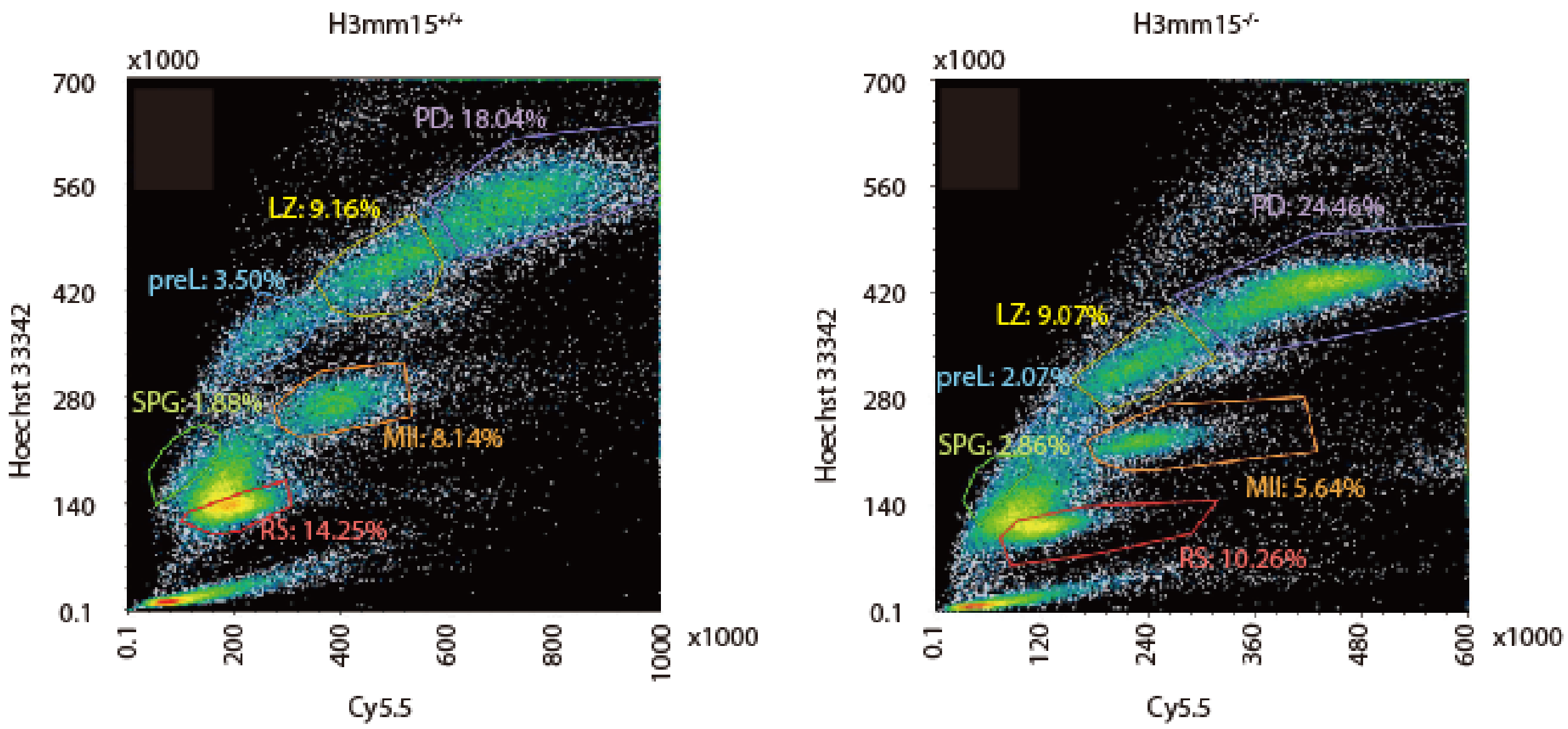

Supplementary Figure S4.
Fluorescence-Activated Cell Sorter (FACS) results of *H3mm15*$^{+/+}$ and *H3mm15*$^{-/-}$ testis. Spermatogenic cells from *H3mm15*$^{+/+}$ or *H3mm15*$^{-/-}$ were divided into six groups (SG, spermatogonia; preL, preleptotene; LZ, leptotene/zygotene; PD, pachytene/diplotene; MII, meiosis II; RS, round spermatid).

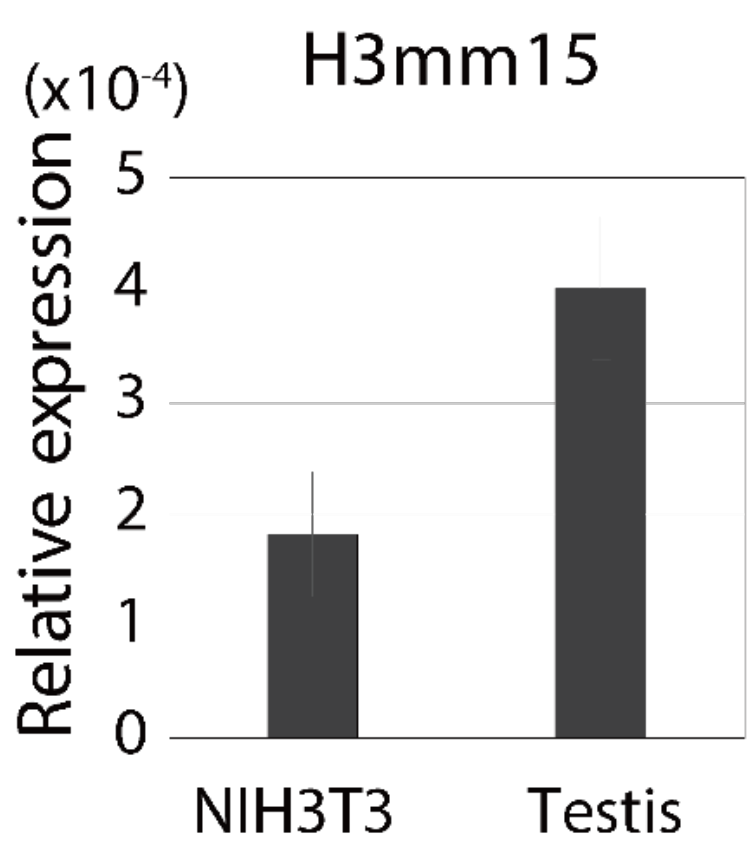

Supplementary Figure S5.
Endogenous expression of H3mm15 in NIH3T3 cells.
Variant-specific RT-qPCR analysis of endogenous H3mm15 expression in NIH3T3 cells and mouse whole testis. Relative H3mm15 expression was determined using the same assay conditions for both sample types. Bars represent the mean, and error bars indicate ± S.D. from three independent samples. NIH3T3 data were obtained from three independent cell preparations, and testis data were obtained from three individual mice.

Differential expression genes

	UP	DOWN
H3.3 vs WT	42	16
H3mm15 vs WT	3	2

Supplementary Figure S6.

Differentially expressed genes upon overexpression of histone variants in NIH3T3 cells. No significant changes in gene expression were observed following H3mm15 overexpression in NIH3T3 cells.

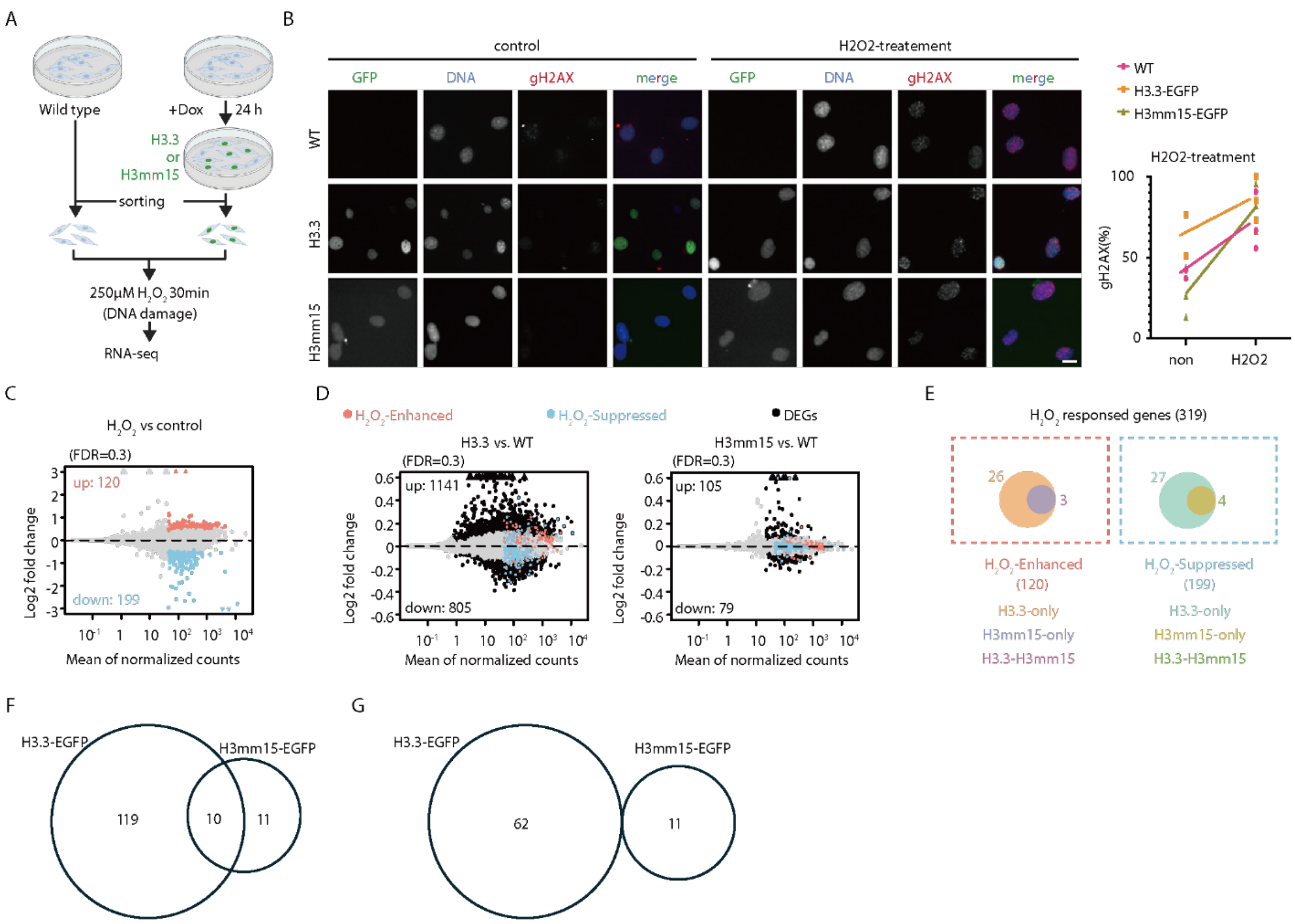

Supplementary Figure S7.

Transcriptional responses to H_2O_2 treatment and cross-condition overlap of H3.3- and H3mm15-associated genes. (A) Overview of the H_2O_2-treatment experiment with WT, H3.3-EGFP-overexpressing, and H3mm15-EGFP-overexpressing cells. Created in BioRender. Harada, A. (2026) https://BioRender.com/8ly1274 **(B)** H_2O_2 treatment resulted in DNA damage in all three cell types. Immunostaining is shown for a DNA damage marker (gH2AX, red), rescued histone variants (GFP, green), and the nucleus (DRAQ5, blue). gH2AX-positive cells were counted before and after H_2O_2 treatment and the results are shown on the right. Representative images from three

independent experiments are shown. gH2AX-positive cells were quantified in three independent experiments, and each point represents one independent experiment. Scale bar, 20 µm. **(C)** MA plot showing the transcriptome profiles based on H_2O_2 treatment. **(D)** MA plot showing the transcriptome profiles based on H3.3 and H3mm15 expression in H_2O_2-treated and untreated samples. **(E)** Venn charts showing the genes induced by histone expression among the H_2O_2-related genes. **(F, G)** Venn diagrams showing genes consistently affected by H3.3-EGFP or H3mm15-EGFP across the two stress conditions. Genes were defined as consistently upregulated (F) or downregulated (G) when they met the differential-expression criteria and showed the same direction of change relative to WT cells under both serum-starvation and H_2O_2-treatment conditions.

A

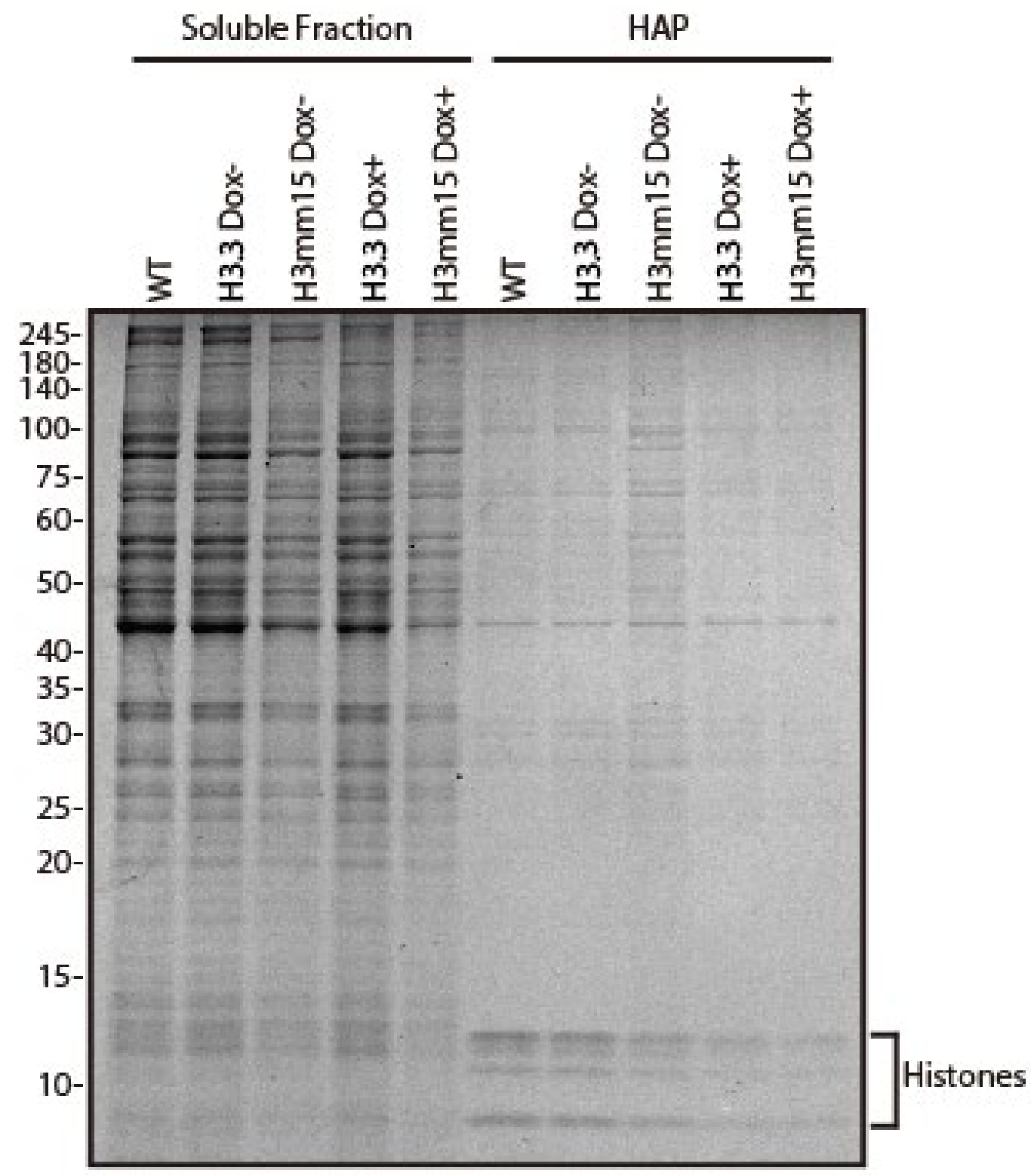
Soluble Fraction
HAP
WT
H3.3 Dox-
H3mm15 Dox-
H3.3 Dox+
H3mm15 Dox+
WT
H3.3 Dox-
H3mm15 Dox-
H3.3 Dox+
H3mm15 Dox+
245-
180-
140-
100-
75-
60-
50-
40-
35-
30-
25-
20-
15-
10-
Histones

B

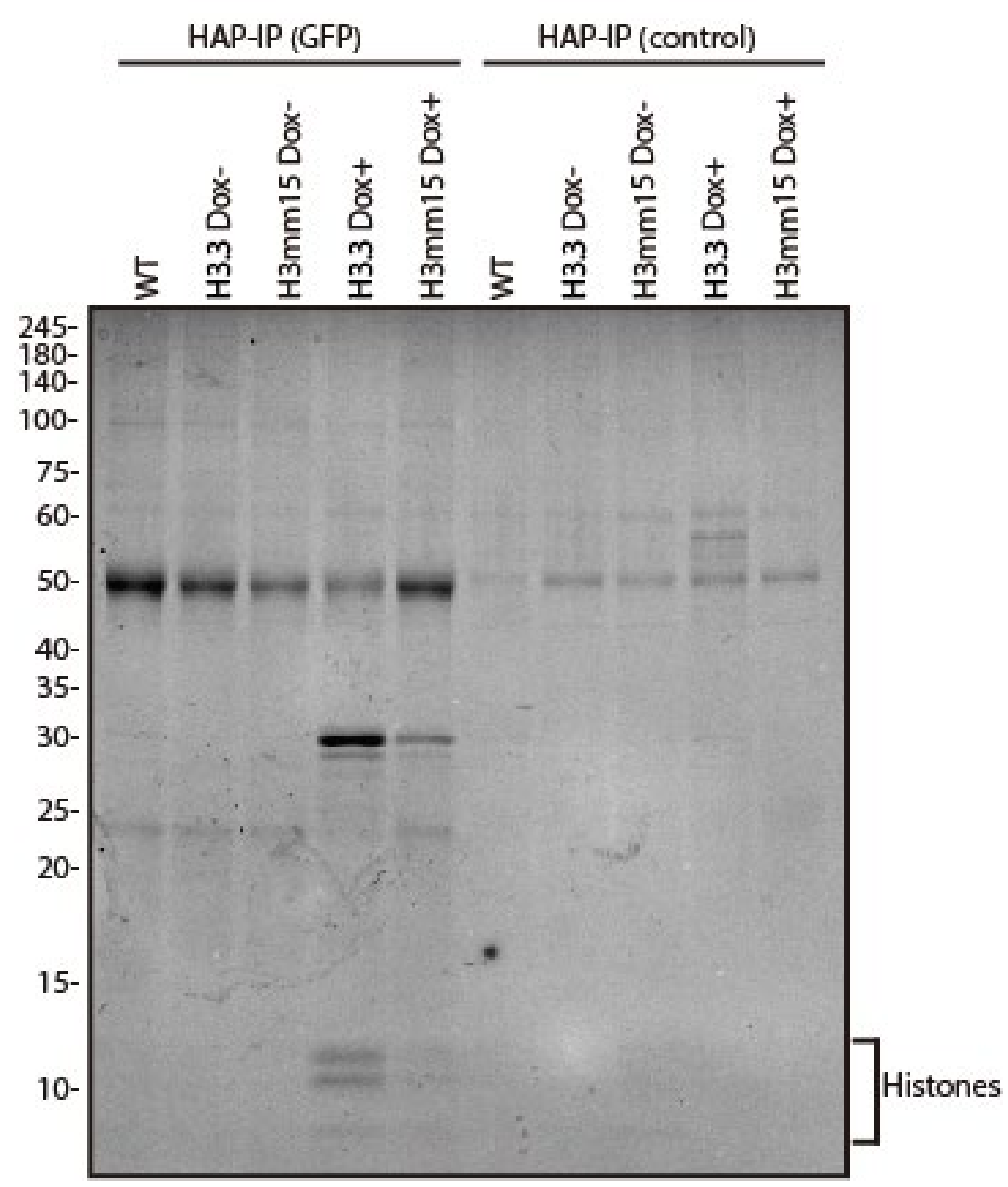
HAP-IP (GFP)
HAP-IP (control)
WT
H3.3 Dox-
H3mm15 Dox-
H3.3 Dox+
H3mm15 Dox+
WT
H3.3 Dox-
H3mm15 Dox-
H3.3 Dox+
H3mm15 Dox+
245-
180-
140-
100-
75-
60-
50-
40-
35-
30-
25-
20-
15-
10-
Histones

Supplementary Figure S8.
Coomassie Brilliant Blue (CBB) staining of H3.3- and H3mm15-overexpressing cells. (A) CBB staining of soluble fraction and hydroxyapatite chromatography (HAP) samples for WT NIH3T3 cells, H3.3-EGFP untreated cells, H3.3-EGFP doxycycline-induced cells, H3mm15-EGFP untreated cells, and H3mm15-EGFP doxycycline-induced cells. Histone signals are marked. **(B)** CBB staining of hydroxyapatite chromatography and immunopurified (HAP-IP) samples for WT NIH3T3 cells, H3f3a-EGFP untreated cells, H3f3a-EGFP doxycycline-induced cells, H3mm15-EGFP untreated cells, and H3mm15-EGFP doxycycline-induced cells. HAP-IP (GFP) samples immunopurified by anti-GFP-conjugated magnetic beads; HAP-IP (control) samples immunopurified by magnetic beads without anti-GFP. Histone signals are marked.

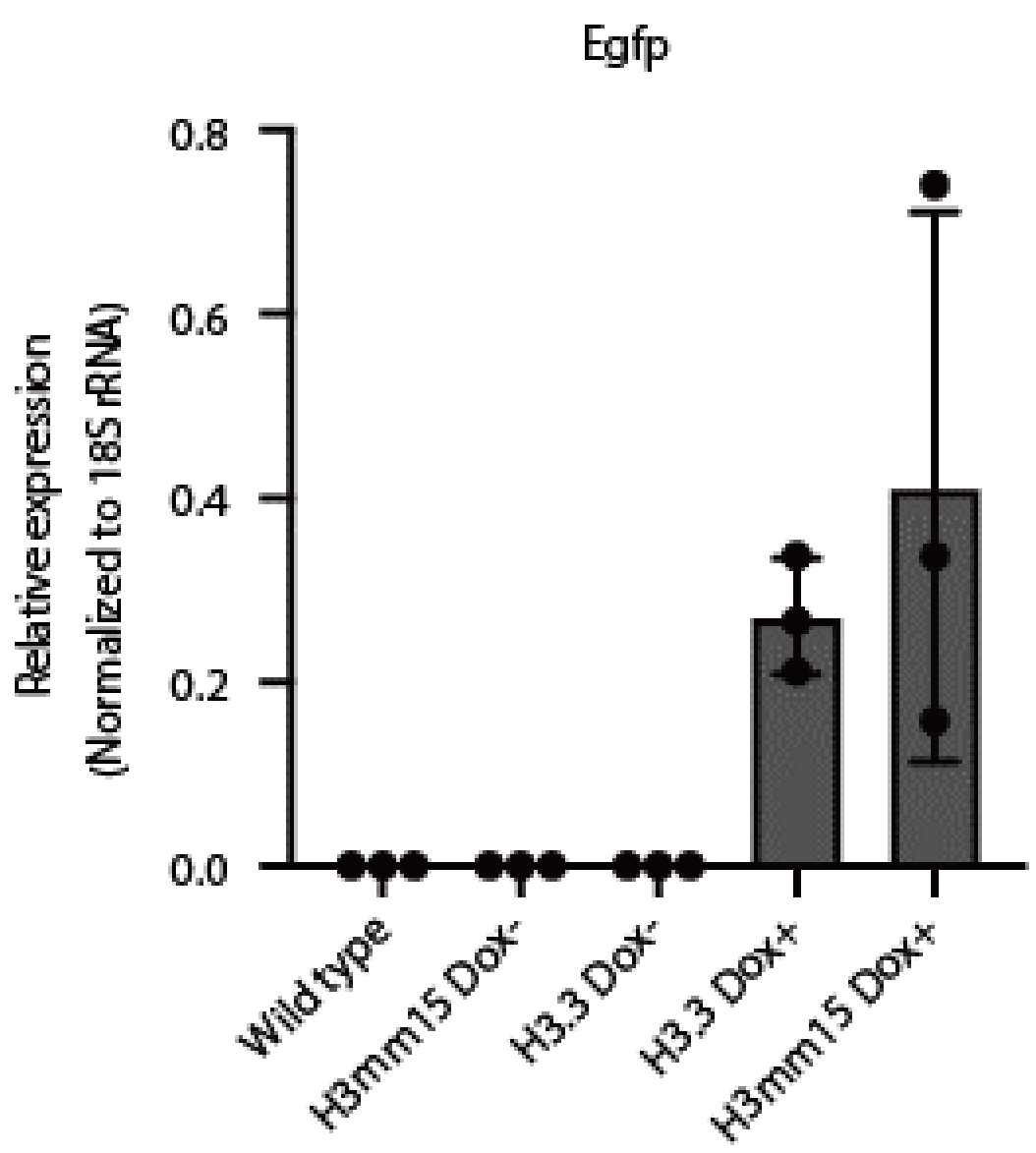

Supplementary Figure S9.
qPCR results for *Egfp* in EGFP-tagged histone variant-expressing or non-expressing NIH3T3 cells. *Egfp* expression was normalized to 18S rRNA expression (n = 3). The error bars indicate ± S.D.

Feature	H3.3 #1	H3.3 #2	H3mm15 #1	H3mm15 #2
Promoter (1-2kb)	4.85	4.58	4.93	4.64
Promoter (<=1kb)	7.83	7.18	9.54	8.30
5' UTR	0.06	0.06	0.05	0.06
3' UTR	1.89	1.79	1.72	1.69
1st Exon	0.84	0.80	0.81	0.80
Other Exon	1.85	1.83	1.80	1.82
1st Intron	14.56	14.27	14.13	13.98
Other Intron	30.01	29.65	28.02	28.42
Downstream (<=300)	0.14	0.13	0.13	0.12
Distal Intergenic	37.96	39.71	38.87	40.16

Supplementary Figure S10.
The distribution of expressed H3mm15-EGFP and H3.3-EGFP in the mouse genome. 5′-UTR, 5′-untranslated region in Figure 4F; 3′-UTR, 3′-untranslated region.

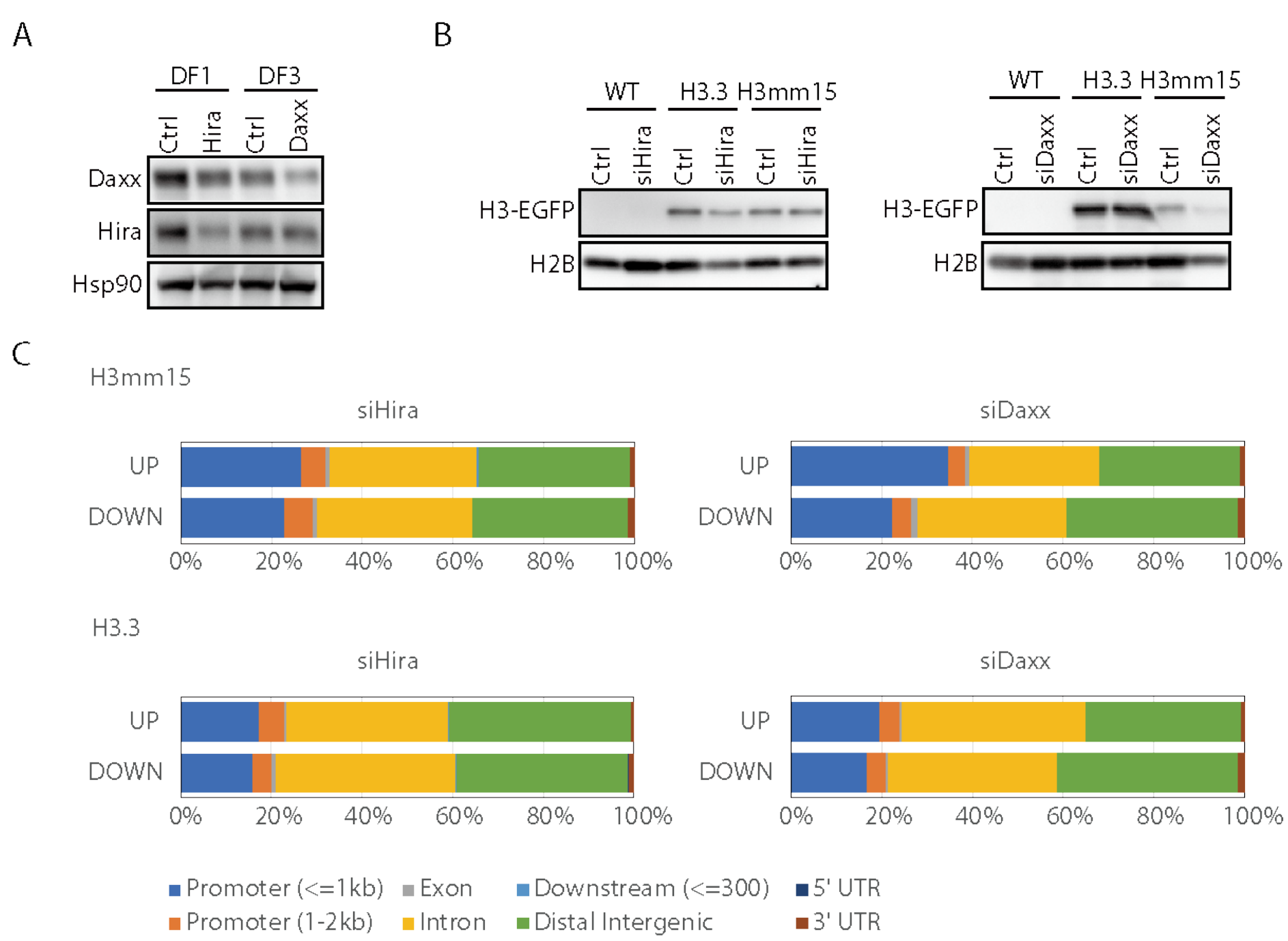

Supplementary Figure S11.
Effects of HIRA and DAXX depletion on the chromatin incorporation and genomic distribution of H3.3-EGFP and H3mm15-EGFP. (A) Immunoblot analysis confirming siRNA-mediated depletion of HIRA and DAXX in whole-cell lysates. HSP90 was used as a loading control. HIRA and DAXX protein levels were reduced by approximately 57% and 65%,

respectively. Because different transfection reagents were used to optimize *Hira* and *Daxx* knockdown, each siRNA treatment was compared with its respective matched control. Representative immunoblots from two independent siRNA transfection experiments are shown. **(B)** Immunoblot analysis of hydroxyapatite-purified nucleosome fractions from WT, H3.3-EGFP-expressing, and H3mm15-EGFP-expressing NIH3T3 cells following *Hira* or *Daxx* knockdown. EGFP-tagged histones were detected using an anti-GFP antibody, and H2B was used as a nucleosomal loading control. Representative immunoblots from two independent siRNA transfection experiments are shown. **(C)** Genomic-feature distributions of regions with relatively increased or decreased GFP ChIL-seq signals following Hira or Daxx knockdown in H3mm15-EGFP- and H3.3-EGFP-expressing cells. Peaks were called from pooled biological replicates and ranked according to the log2 fold change in GFP ChIL-seq signal between the siRNA-treated samples and their respective matched controls. The top 1,000 and bottom 1,000 regions were classified as the relatively increased and relatively decreased groups, respectively. Bars indicate the percentages of regions assigned to promoters within 1 kb of a transcription start site, promoters 1–2 kb from a transcription start site, 5′ untranslated regions, exons, introns, 3′ untranslated regions, downstream regions within 300 bp, or distal intergenic regions. Genomic annotations were assigned using ChIPseeker. Following Daxx knockdown in H3mm15-EGFP-expressing cells, promoter-proximal annotations were enriched in the relatively increased group compared with the relatively decreased group (Fisher's exact test, odds ratio ≈ 1.85, $P \approx 1 \times 10^{-9}$), whereas distal intergenic annotations were depleted (odds ratio ≈ 0.74, $P \approx 2 \times 10^{-3}$). Two biologically independent experiments were performed for each condition, and the replicates were pooled before peak calling.

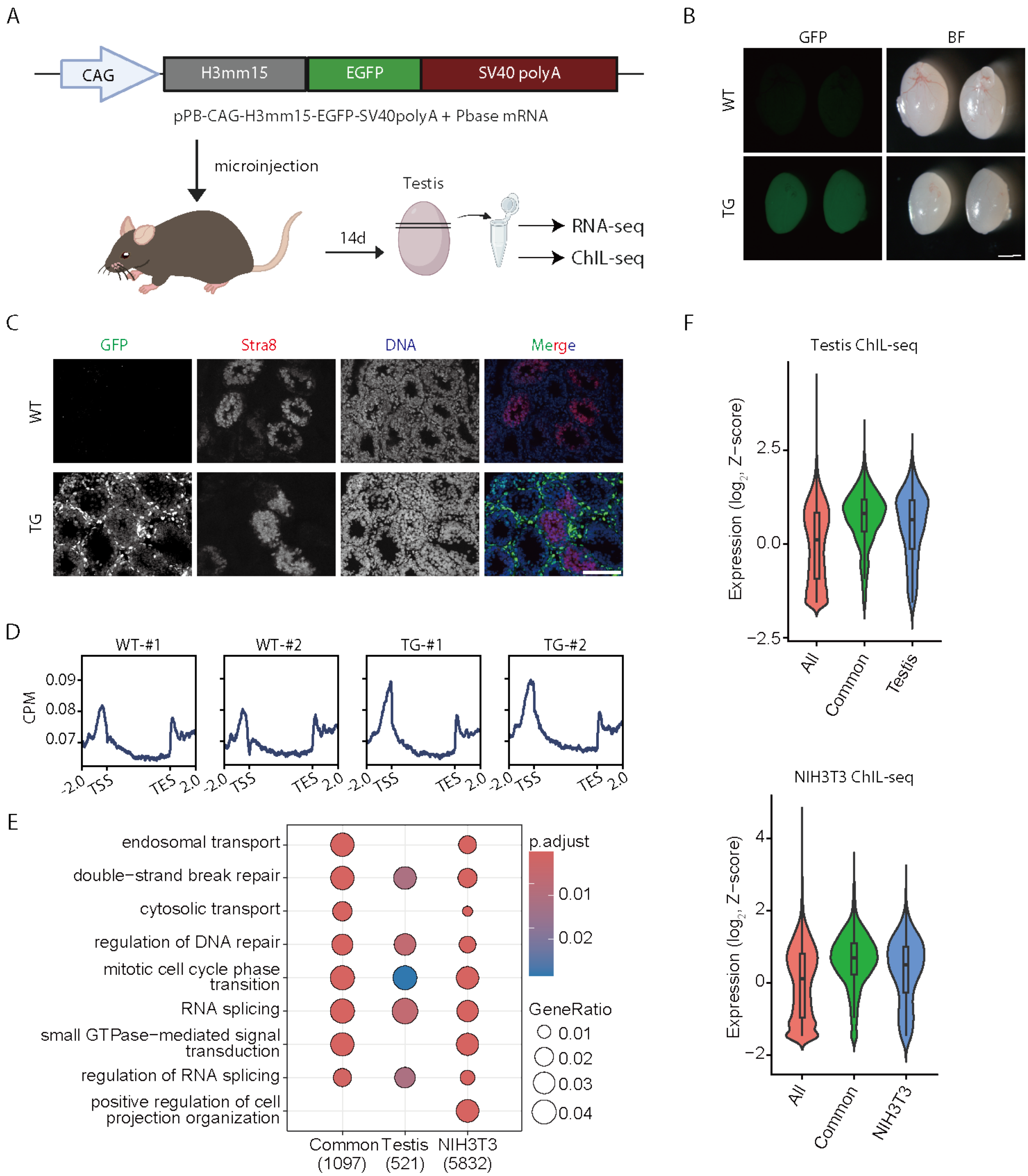

Supplementary Figure S12.

H3mm15-EGFP shows promoter-proximal enrichment in developing transgenic testis. (A) Schematic of the generation and analysis of H3mm15-EGFP transgenic mice. A CAG-driven H3mm15-EGFP expression cassette containing an SV40 polyadenylation signal was introduced into mouse zygotes using the piggyBac transposon system. Testes were collected from wild-type (WT) and transgenic (TG) mice at postnatal day 14 (P14) for fluorescence imaging, immunohistochemistry, RNA-seq, and GFP ChIL-seq analyses. **(B)** Representative bright-field

and EGFP fluorescence images of testes from P14 WT and H3mm15-EGFP TG mice. Clear EGFP fluorescence was detected in TG testes but not in WT littermates. Scale bar, 1 mm. **(C)** Representative immunofluorescence images of P14 TG testis sections stained for GFP and STRA8. DNA was counterstained with Nuclear Violet DCS1 dye. H3mm15-EGFP was detected within seminiferous tubules, including STRA8-positive germ cells. Scale bar, 100 μm. **(D)** Metagene profiles of GFP ChIL-seq signals across gene bodies in testes from WT and H3mm15-EGFP TG mice. Profiles are shown from transcription start sites (TSSs) to transcription end sites (TESs), including the indicated upstream and downstream regions. Lines represent individual biological replicates from WT and TG testes. H3mm15-EGFP TG testes showed pronounced signal enrichment around TSSs and a smaller signal around TESs relative to WT controls. **(E)** Gene Ontology biological process enrichment analysis of genes associated with H3mm15-EGFP in both testis and NIH3T3 cells (Common), only in testis (Testis-restricted), or only in NIH3T3 cells (NIH3T3-restricted). Dot size represents the number of genes assigned to each term, and color indicates the adjusted P value. The original gene sets contained 1,213 common, 612 testis-restricted, and 6,659 NIH3T3-restricted genes. Numbers in parentheses indicate the genes successfully mapped for Gene Ontology analysis. **(F)** Expression distributions of all expressed genes and H3mm15-EGFP-associated gene sets in P14 testis and NIH3T3 cells. Gene-expression values are shown as standardized Z scores. The Common group comprises genes associated with H3mm15-EGFP in both systems, whereas Testis-restricted and NIH3T3-restricted indicate genes associated with H3mm15-EGFP only in the respective system. Center lines indicate medians, boxes indicate interquartile ranges, and whiskers extend to 1.5 times the interquartile range.

A

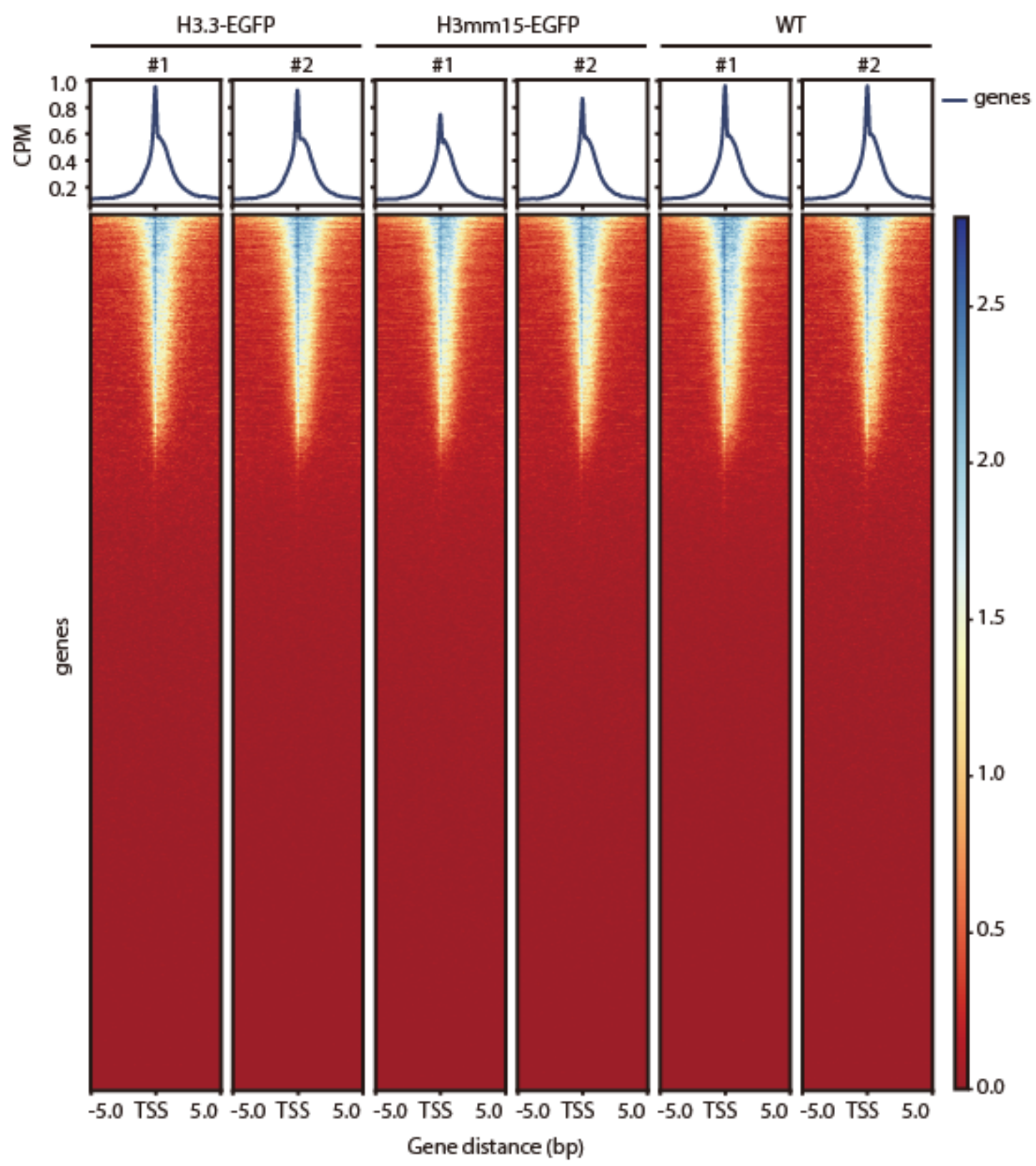

H3.3-EGFP
H3mm15-EGFP
WT
#1
#2
CPM
1.0
0.8
0.6
0.4
0.2
genes
2.5
2.0
1.5
1.0
0.5
0.0
-5.0 TSS 5.0
Gene distance (bp)

B

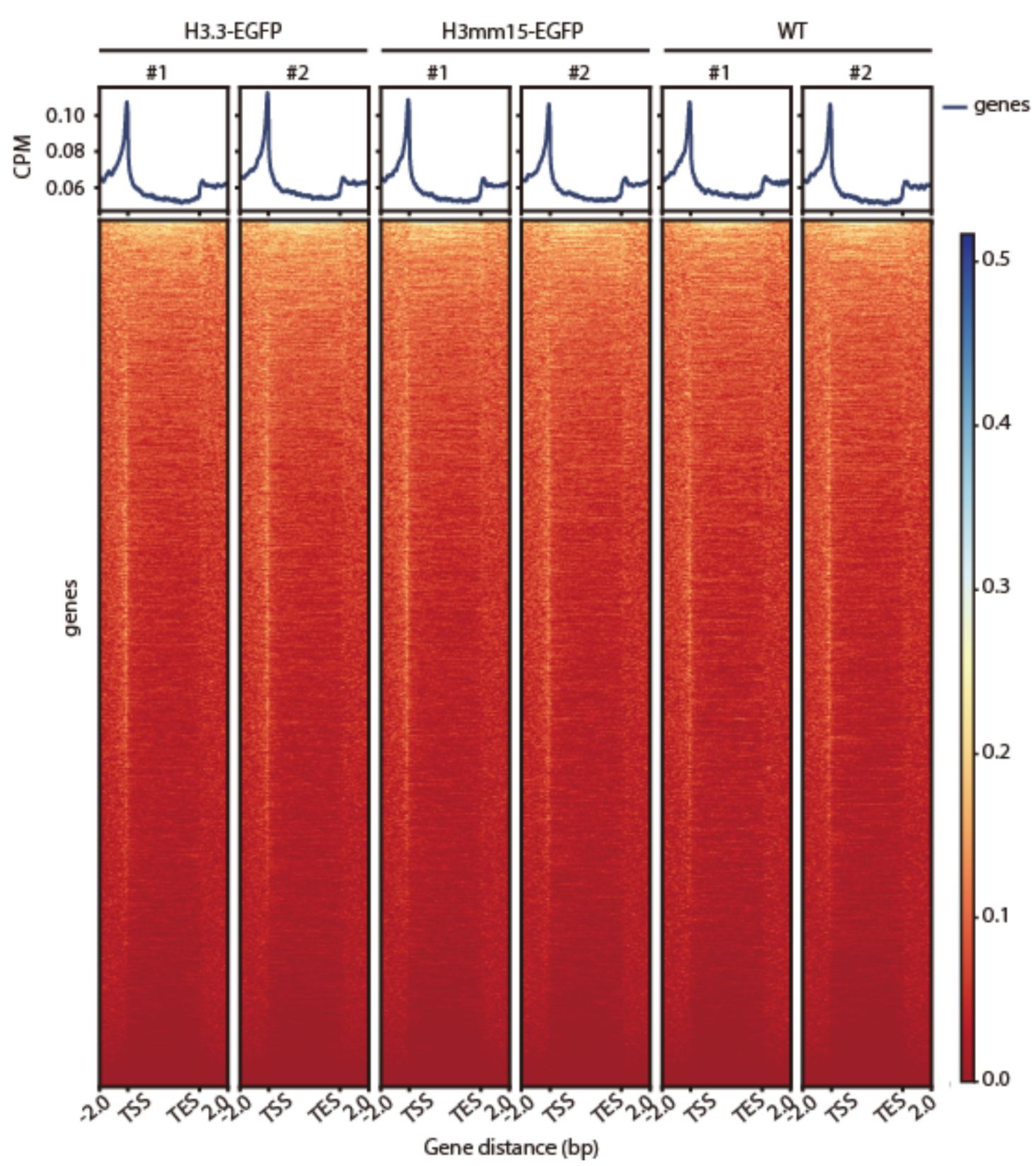

H3.3-EGFP
H3mm15-EGFP
WT
#1
#2
CPM
0.10
0.08
0.06
genes
0.5
0.4
0.3
0.2
0.1
0.0
-2.0 TSS TES 2.0
Gene distance (bp)

Supplementary Figure S13.
Global histone modification levels of H3.3-EGFP- and H3mm15-EGFP-expressing cells and WT NIH3T3 cells. (A) Global H3K4me3 levels of H3.3-EGFP- and H3mm15-EGFP-expressing cells and WT NIH3T3 cells. The line plot and heatmap show the H3K4me3-ChIL signal levels (count per million, CPM) on gene promoters (TSS ± 5 kb). The genes (rows) are ordered by H3K4me3 signal levels. **(B)** Global H3K27me3 levels of H3.3-EGFP- and H3mm15-EGFP-expressing cells and WT NIH3T3 cells. The line plot and heatmap show the H3K27me3-ChIL signal levels (CPM) on the gene body (TSS −2 kb to TES +2 kb). The genes (rows) are ordered by H3K27me3 signal levels.

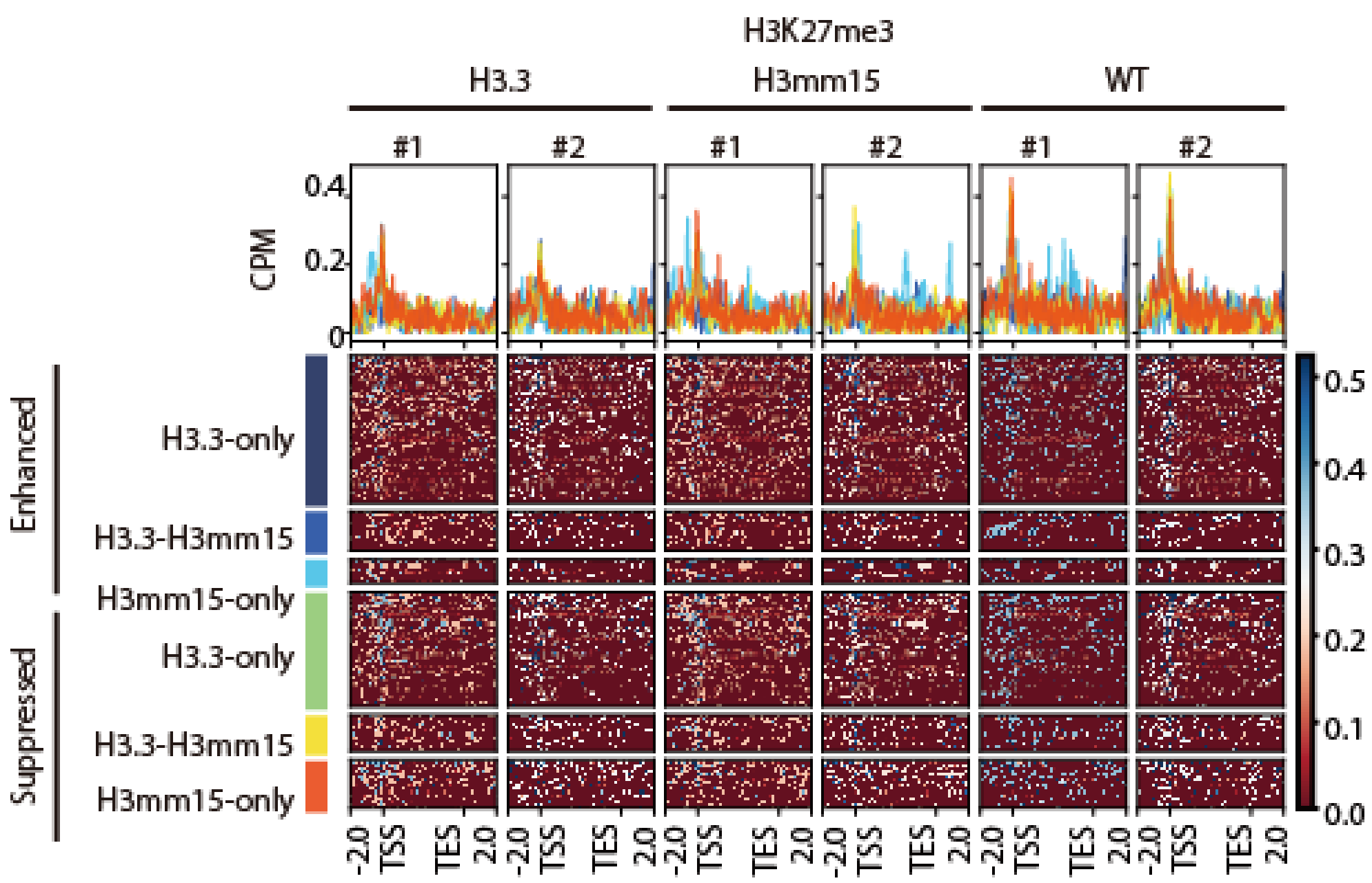

Supplementary Figure S14.
Comparison of the incorporation level of EGFP-tagged histone variants and the H3K27me3 levels of the genes shown in Figure 3G.

A

Differential accessibility genes

	UP	DOWN
H3.3 vs WT	205	111
H3mm15 vs WT	29	0

B

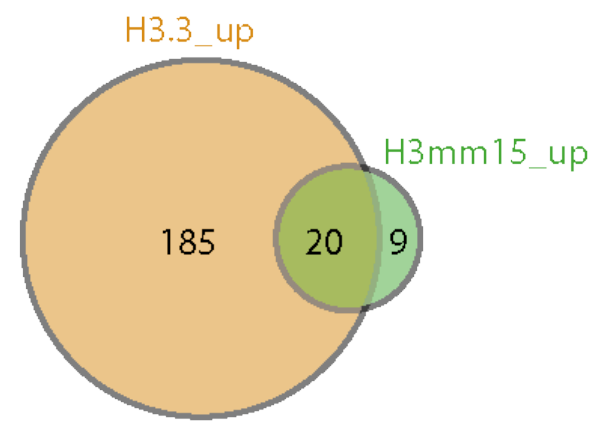

Supplementary Figure S15.

H3mm15 regulates chromatin accessibility more moderately than H3.3 under stable conditions. (A) Numbers of differentially accessible genes due to H3.3 or H3mm15 overexpression compared with WT NIH3T3 cells. **(B)** Overlap of the upregulated differentially accessible genes due to H3.3-EGFP or H3mm15-EGFP expression in Supplementary Figure S15A.

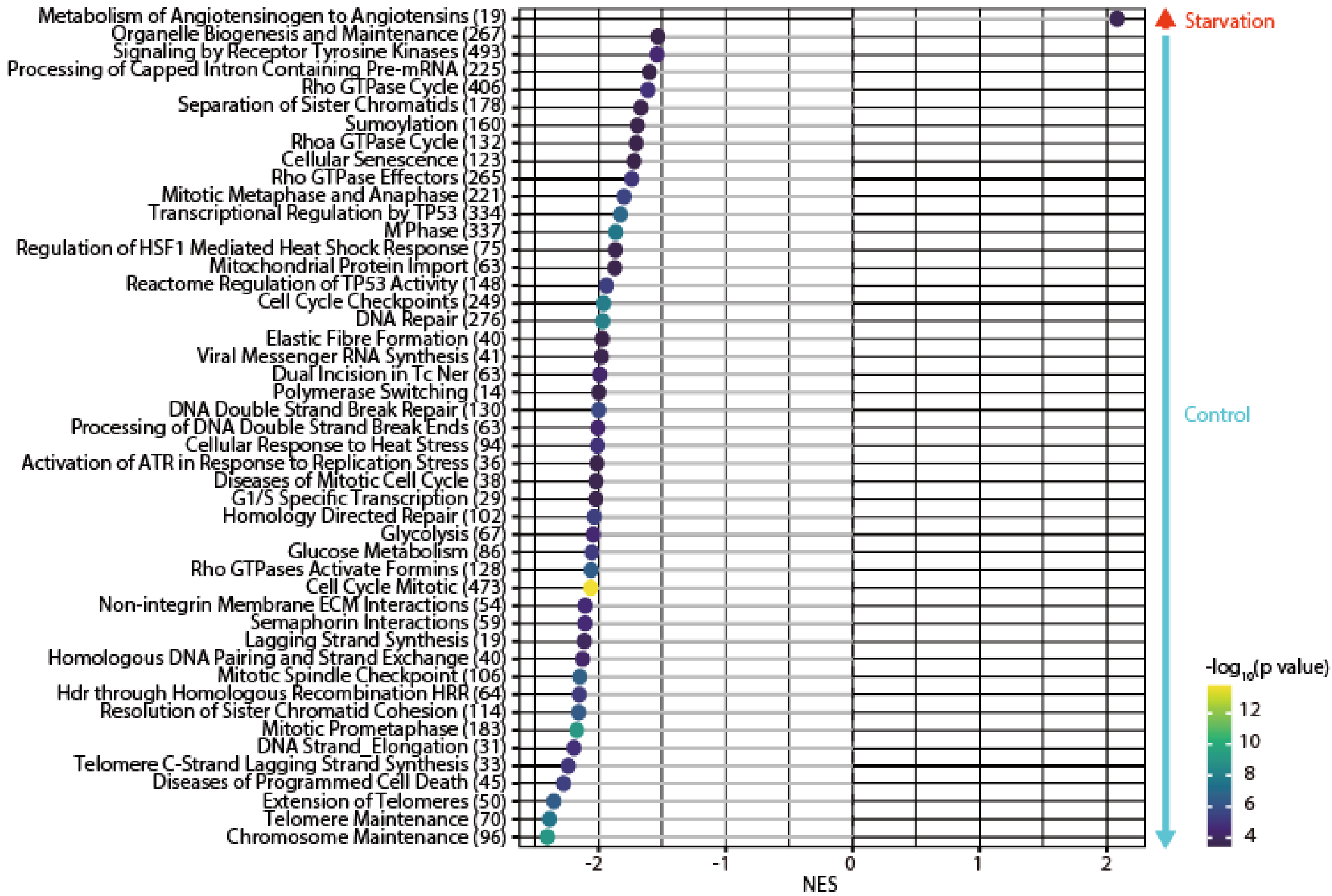

Supplementary Figure S16.

Bar graph of the NES of the hallmark gene sets in the GSEA of the differentially accessible genes in WT cells upon serum-starvation treatment. The enriched gene sets of the upregulated and downregulated genes in serum-starved NIH3T3 cells are indicated.

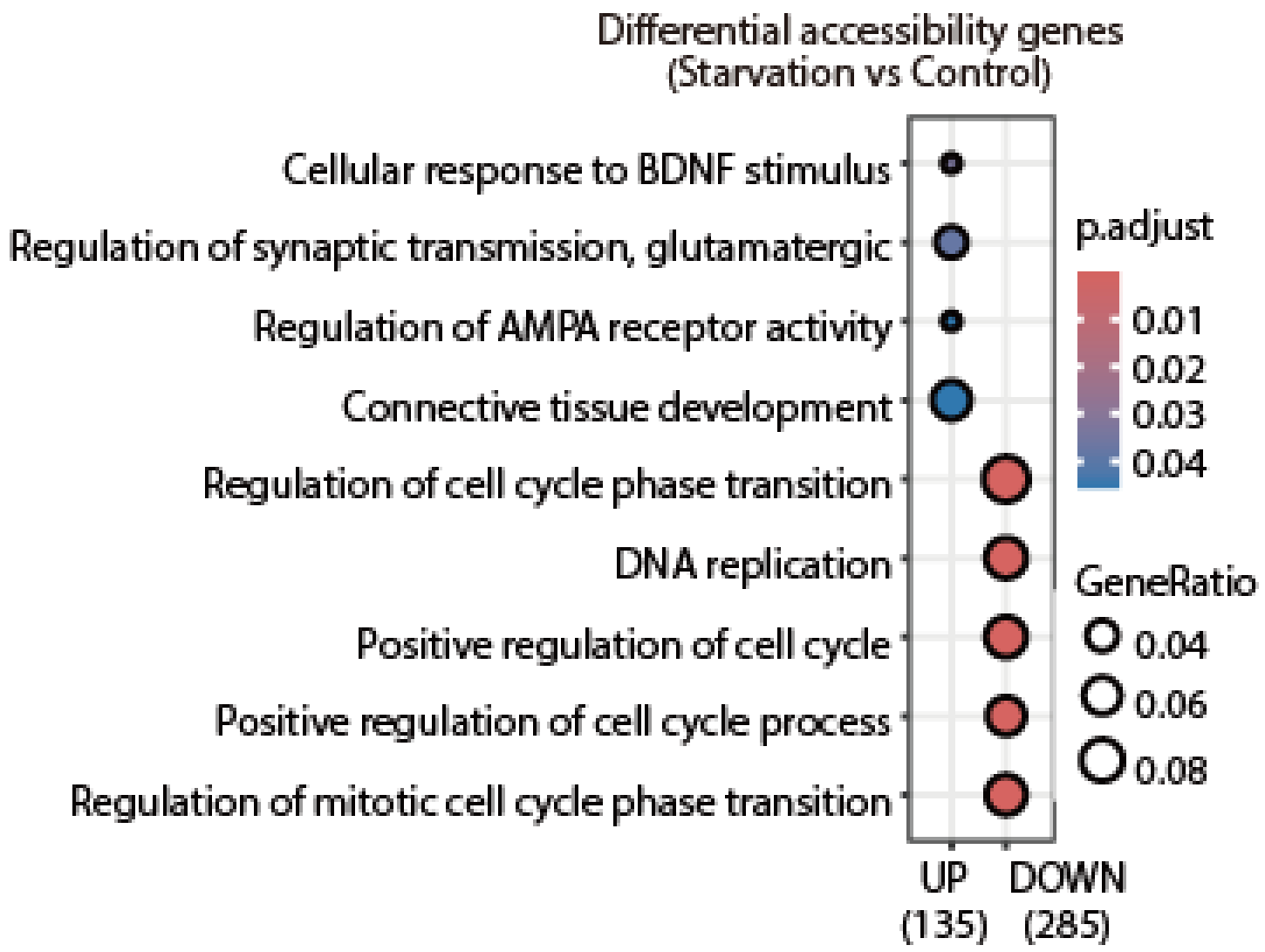

Supplementary Figure S17.
Gene Ontology (GO) analysis of the differentially accessible genes in NIH3T3 WT cells upon serum-starvation treatment.

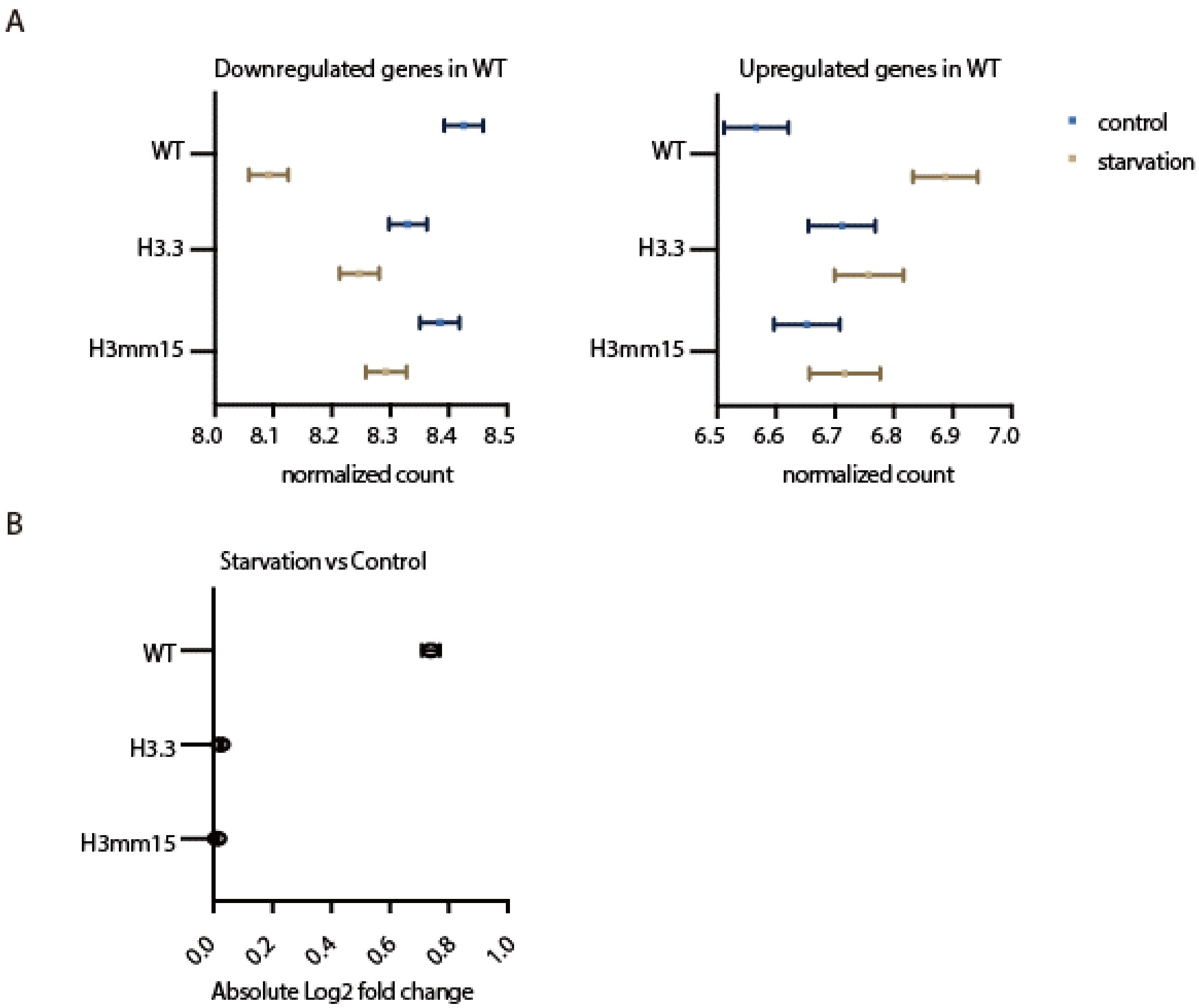

Supplementary Figure S18.
H3mm15 modulates chromatin accessibility dynamics during serum starvation. (A) Average normalized counts of downregulated or upregulated differentially accessible genes under control or serum-starvation conditions in WT, H3.3-EGFP-expressing, and H3mm15-EGFP-expressing NIH3T3 cells. The error bars indicate ± standard error of the mean (S.E.M.). **(B)** Absolute $\log_2$(fold change) of the differentially accessible genes under starvation conditions in WT, H3.3-EGFP-expressing, and H3mm15-EGFP-expressing NIH3T3 cells. Both H3.3-EGFP- and H3mm15-EGFP-expressing cells show low absolute $\log_2$(fold change) values compared with WT, indicating attenuated starvation-induced accessibility changes. The error bars indicate ± S.E.M.

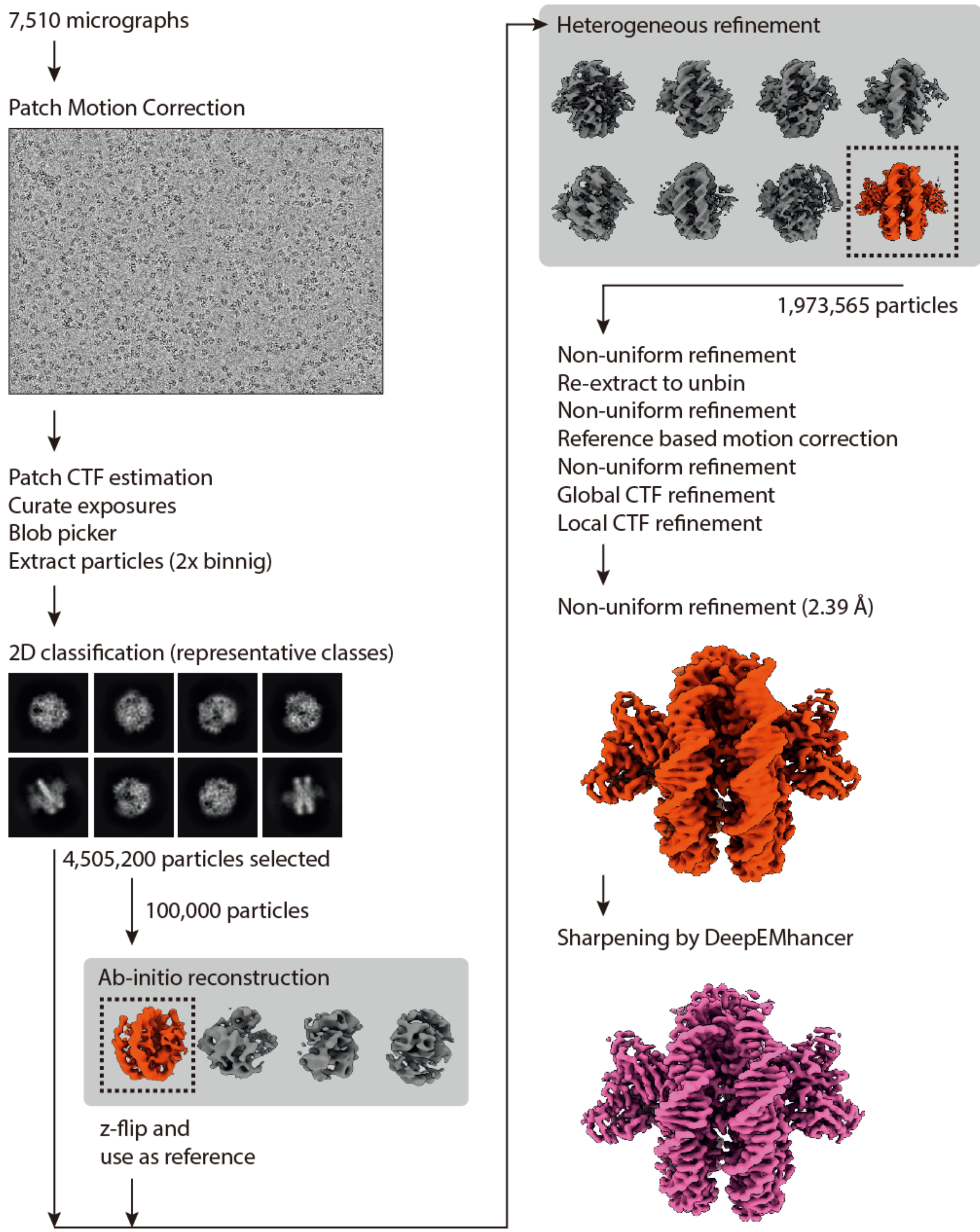

Supplementary Figure S19.
Workflow for cryo-EM single particle analysis of the H3mm15 nucleosome with PL2-6 scFv.

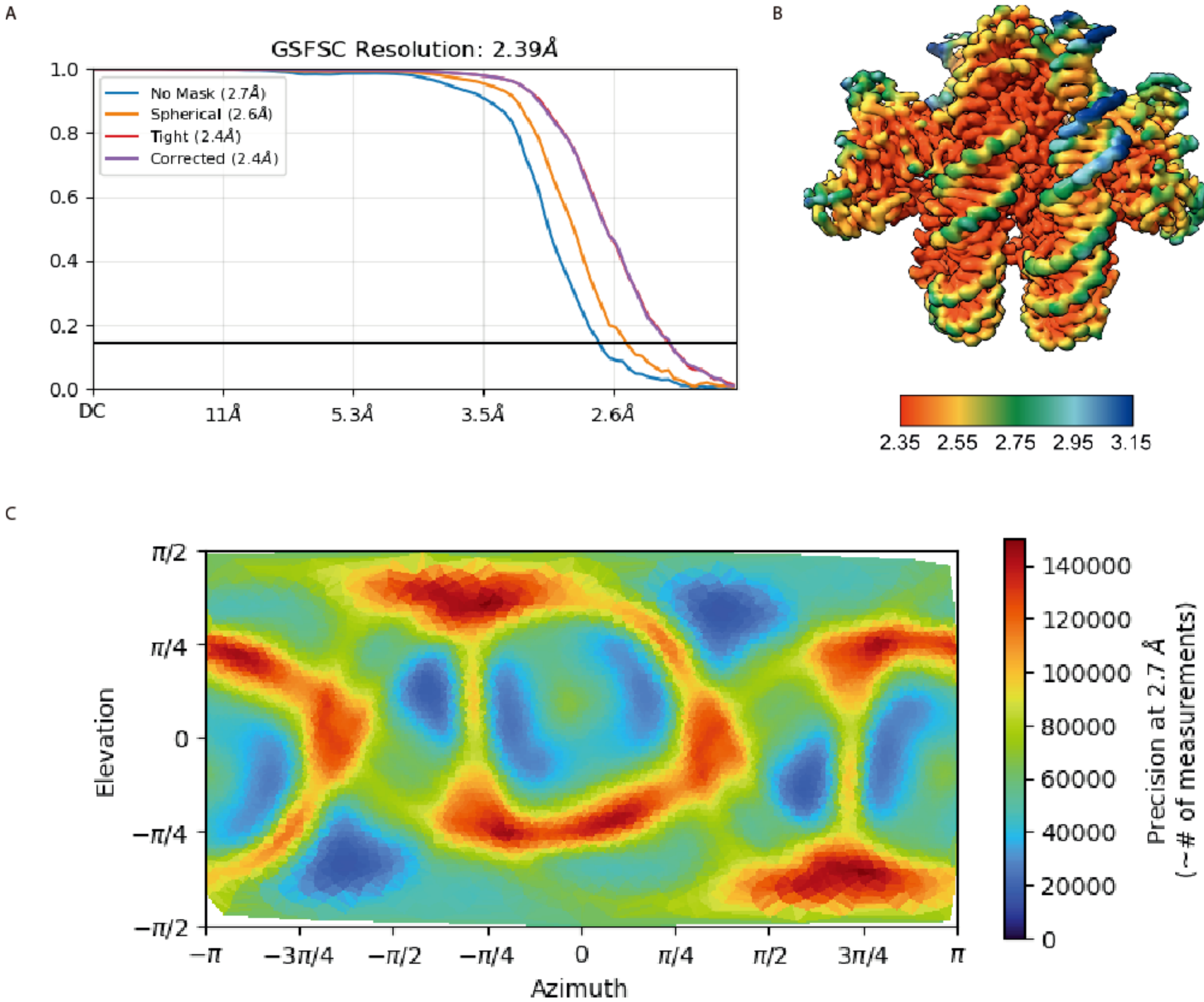

Supplementary Figure S20.

Cryo-EM analysis of the H3mm15 nucleosome with PL2-6 scFv. (A) Fourier Shell Correlation (FSC) curve. The resolution is estimated to be 2.39 Å by an FSC = 0.143. **(B)** Local resolution map. **(C)** Angular distribution.

A

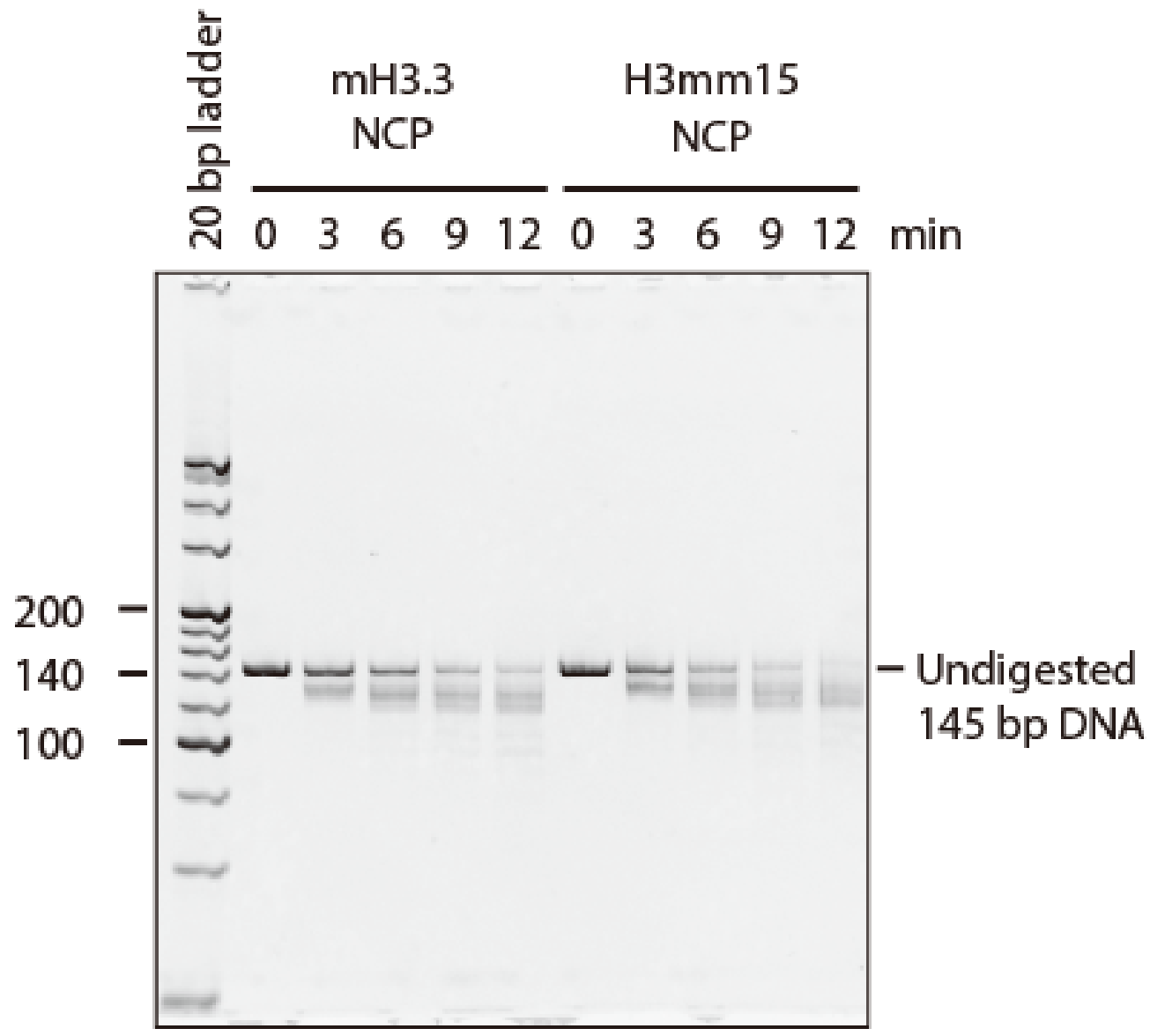

B

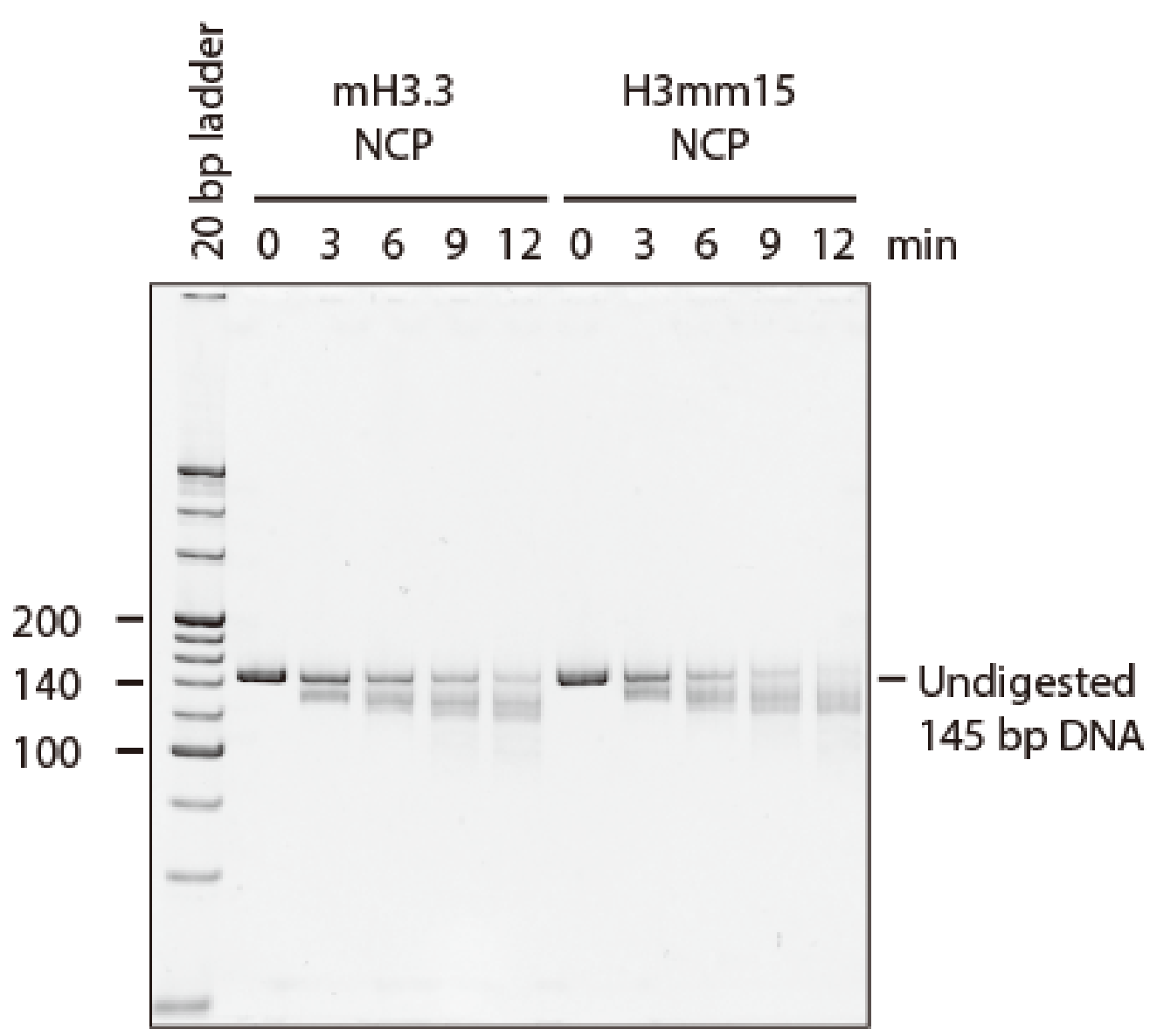

Supplementary Figure S21.
Replicates of the MNase assay in Figure 5F. **(A)** First replicate. **(B)** Second replicate.

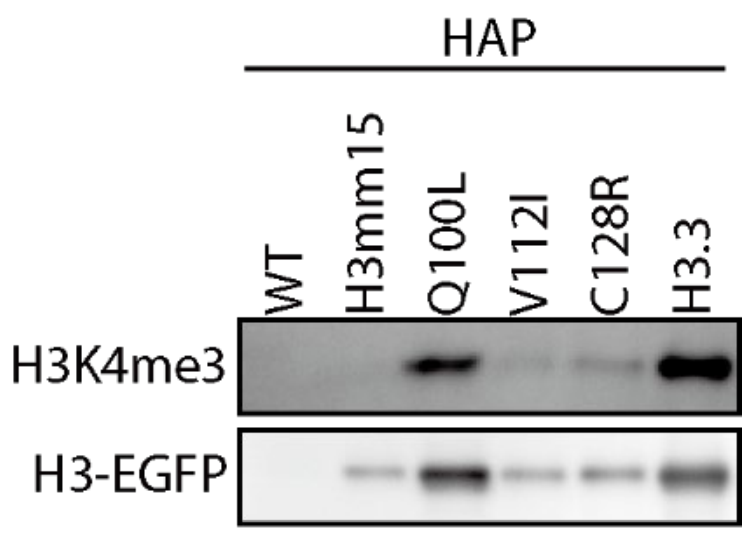

Supplementary Figure S22.
H3K4me3 modification of chromatin-incorporated EGFP-tagged H3 variants. HAP-purified nucleosomal fractions from WT NIH3T3 cells and cells expressing H3mm15-EGFP, H3mm15-Q100L-EGFP, H3mm15-V112I-EGFP, H3mm15-C128R-EGFP, or H3.3-EGFP were analyzed by immunoblotting with anti-H3K4me3 and anti-GFP antibodies (Figure 6). H3K4me3-positive signals were detected at the expected molecular weight of the H3-EGFP fusion proteins. WT cells were included as a negative control for the high-molecular-weight signal. The intensity of the H3K4me3 signals broadly paralleled the amount of H3-EGFP recovered in the HAP fraction. Representative immunoblots from two independent HAP purification experiments are shown.

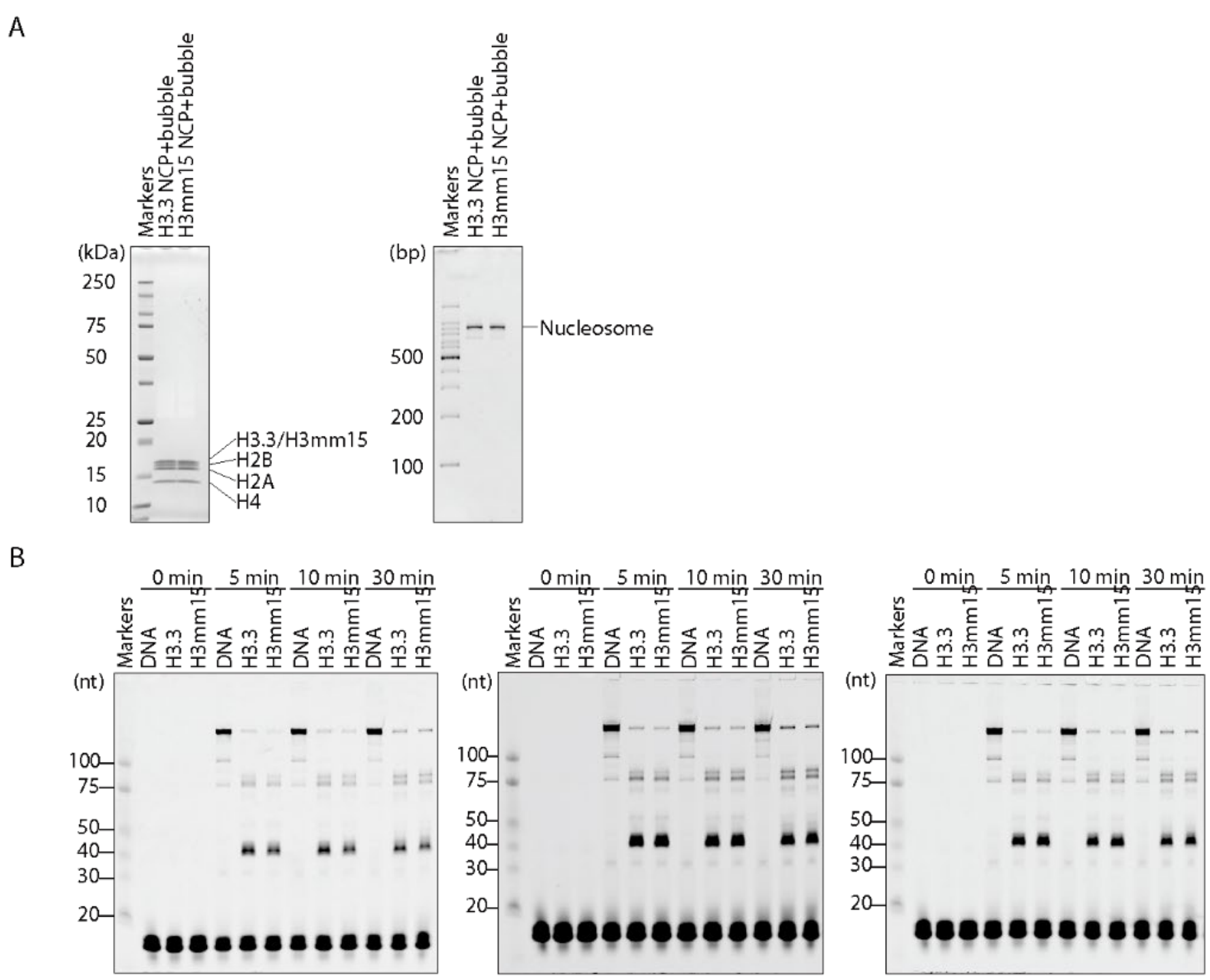

Supplementary Figure S23.
Validation of nucleosomal templates and reproducibility of the in vitro transcription assay. **(A)** Validation of the H3.3- and H3mm15-containing nucleosomes used for the transcription assay. Nucleosomal proteins were analyzed by SDS–PAGE and visualized by Coomassie Brilliant Blue staining, confirming the presence of H2A, H2B, H3.3 or H3mm15, and H4. Reconstituted nucleosomes containing the ligated mismatch-bubble DNA were analyzed by native PAGE, and DNA was visualized by ethidium bromide staining. **(B)** Reproducibility of the in vitro transcription assay. Transcription reactions were performed using naked DNA and nucleosomes containing either H3.3 or H3mm15 for 0, 5, 10, or 30 min. DY647-labeled RNA products were separated by

denaturing PAGE. The results of all three independent transcription assays, including the representative experiment shown in Figure 7B, are presented.

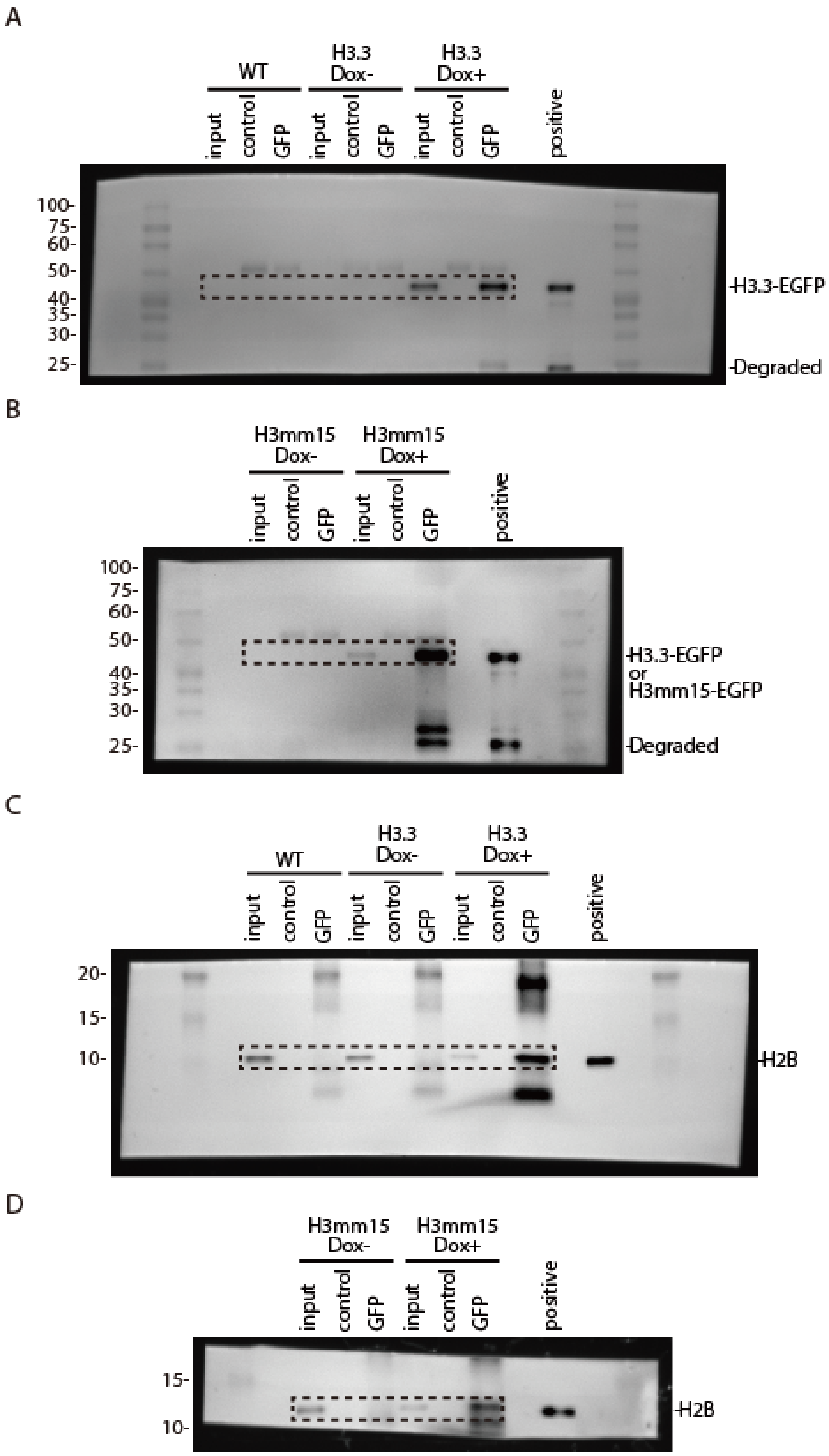

Supplementary Figure S24.
Uncropped immunoblotting images. The dotted boxes indicate the cropped regions presented in Figure 4B.

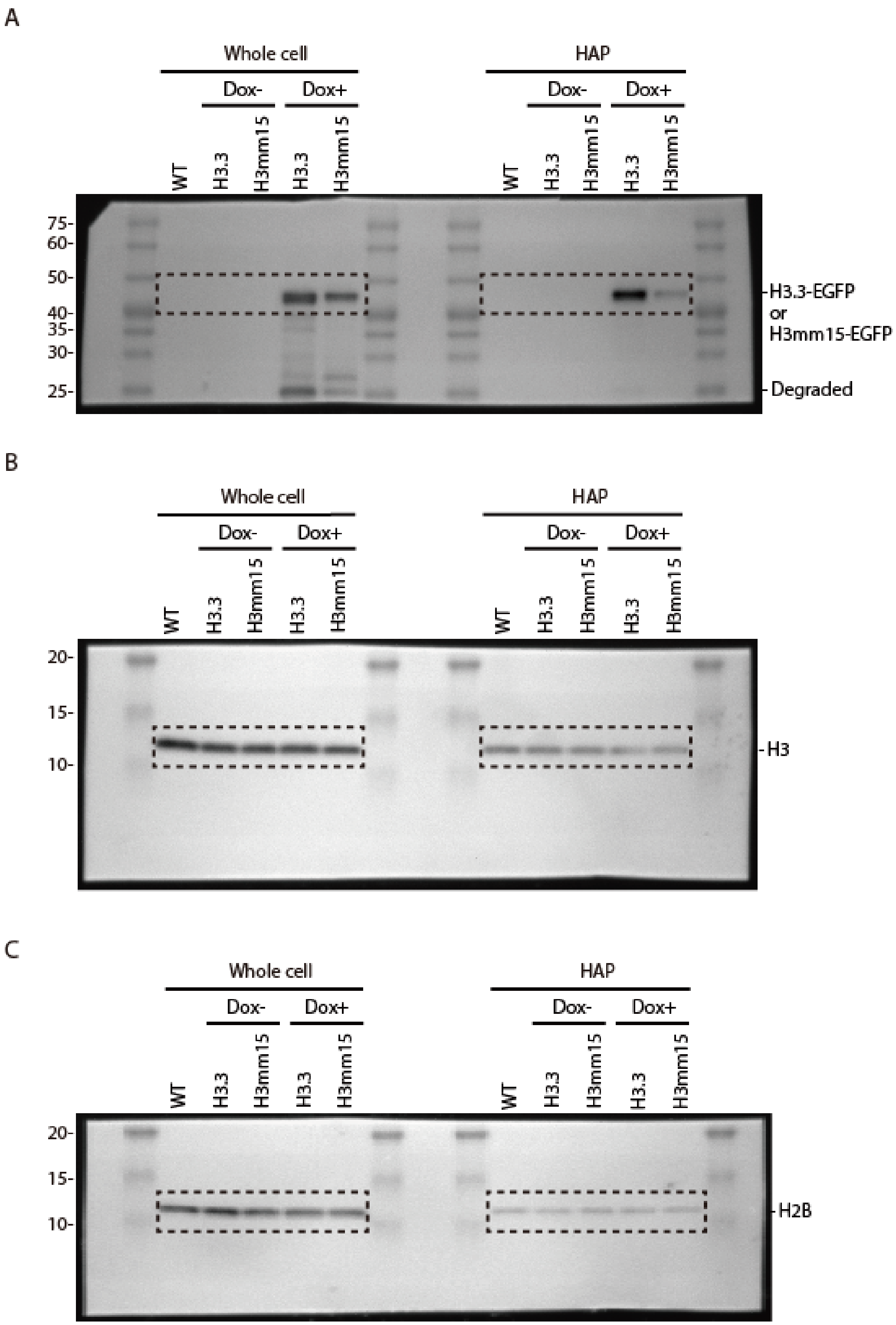

Supplementary Figure S25.
Uncropped immunoblotting images. The dotted boxes indicate the cropped regions presented in Figure 4C and D.

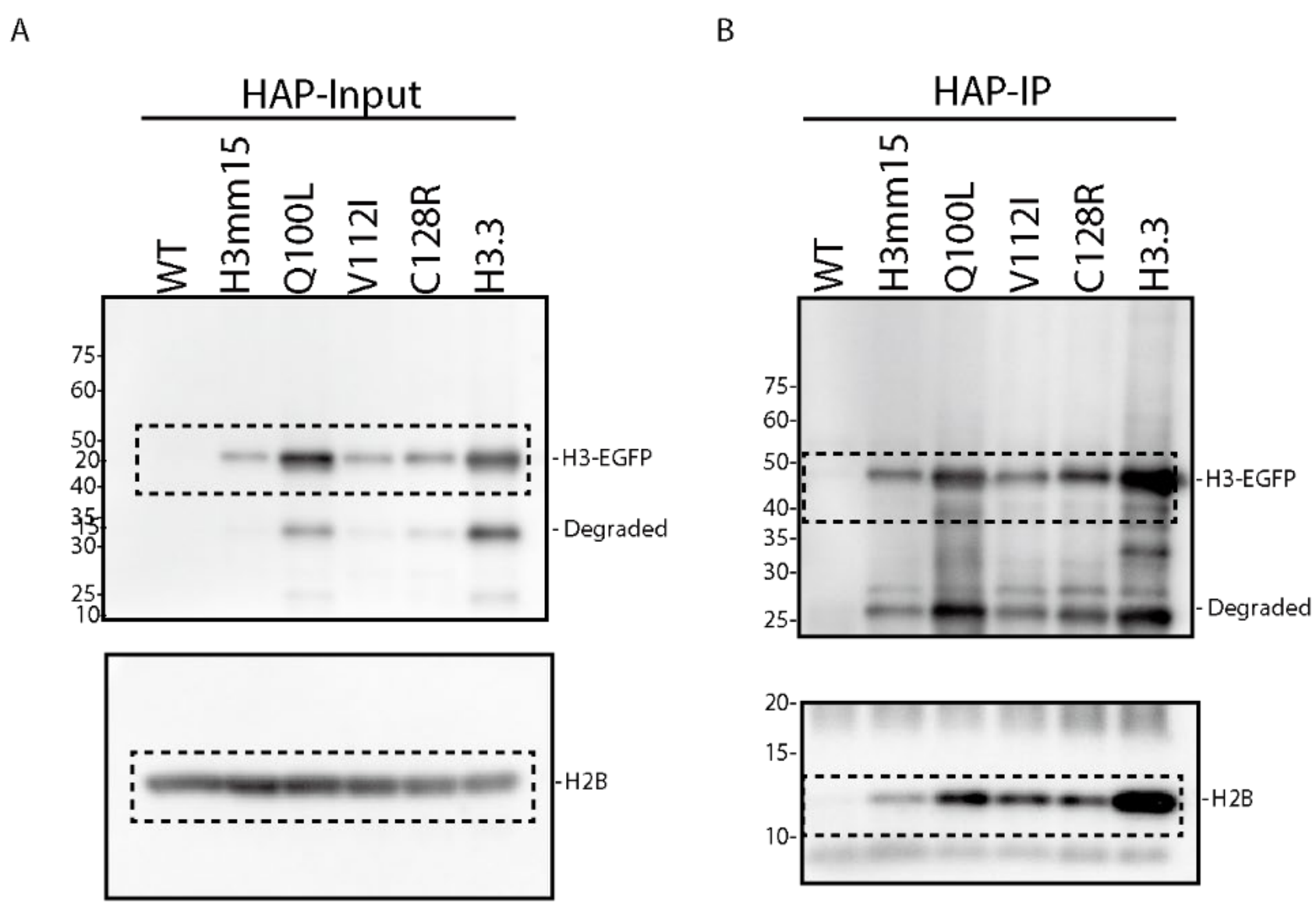

Supplementary Figure S26.
Uncropped immunoblotting images. The dotted boxes indicate the cropped regions presented in Figure 6C.
